# Rational design of dinitroxide biradicals for efficient cross-effect dynamic nuclear polarization†
†Electronic supplementary information (ESI) available: Additional experimental details, sample compositions and synthetic routes of radicals. See DOI: 10.1039/c5sc02921j
‡These authors contributed equally to this work.


**DOI:** 10.1039/c5sc02921j

**Published:** 2015-10-13

**Authors:** Dominik J. Kubicki, Gilles Casano, Martin Schwarzwälder, Sébastien Abel, Claire Sauvée, Karthikeyan Ganesan, Maxim Yulikov, Aaron J. Rossini, Gunnar Jeschke, Christophe Copéret, Anne Lesage, Paul Tordo, Olivier Ouari, Lyndon Emsley

**Affiliations:** a Institut des Sciences et Ingénierie Chimiques , Ecole Polytechnique Fédérale de Lausanne (EPFL) , CH-1015 Lausanne , Switzerland . Email: lyndon.emsley@ens-lyon.fr; b Université de Lyon , Institut de Sciences Analytiques (CNRS / ENS de Lyon / UCB-Lyon 1) , Centre de RMN à Très Hauts Champs , 69100 Villeurbanne , France; c Aix-Marseille Université , CNRS , ICR UMR 7273 , 13397 Marseille , France . Email: paul.tordo@univ-amu.fr ; Email: olivier.ouari@univ-amu.fr; d ETH Zurich , Department of Chemistry, Laboratory of Inorganic Chemistry , 8093 Zurich , Switzerland

## Abstract

A series of 37 dinitroxide biradicals have been prepared and their performance studied as polarizing agents in cross-effect DNP NMR experiments at 9.4 T and 100 K in 1,1,2,2-tetrachloroethane (TCE). We observe that in this regime the DNP performance is strongly correlated with the substituents on the polarizing agents, and electron and nuclear spin relaxation times, with longer relaxation times leading to better enhancements. We also observe that deuteration of the radicals generally leads to better DNP enhancement but with longer build-up time. One of the new radicals introduced here provides the best performance obtained so far under these conditions.

## Abstract 2

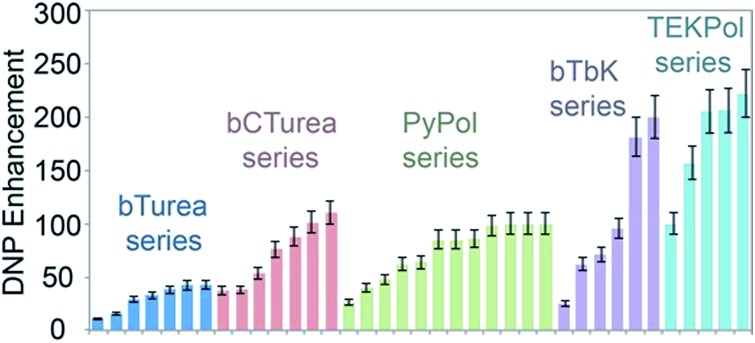
A series of 37 dinitroxide biradicals have been prepared and their performance studied as polarizing agents in cross-effect DNP NMR experiments at 9.4 T and 100 K in 1,1,2,2-tetrachloroethane (TCE).

## Introduction

Dynamic nuclear polarization (DNP)^1^–^3^ currently attracts considerable attention as one of the most efficient methods to increase the sensitivity of NMR experiments.^4^–^9^ One can increase the intrinsically low polarization of nuclear spins by coupling them to unpaired electrons through means of microwave (MW) irradiation. The theoretical limit of the signal enhancement in that process (*ε*_max_) equals *γ*_e_/*γ*_n_, where *γ*_e_ and *γ*_n_ are the gyromagnetic ratios of the electron and the nucleus, respectively (for instance, *ε*_max_ is 660 for proton, and 2618 for carbon-13). For *in situ* high-field solid-state NMR, the unpaired electrons are usually added to the sample in the form of a mono- or biradical, usually derived from tetrathiatriarylmethyl or TEMPO radicals, and the experiment is performed with magic angle spinning (MAS) at temperatures of about 100 K.^6^,^10^–^12^ At these temperatures, currently achievable proton DNP enhancements reach up to around 200 in frozen bulk solutions, and up to 500 in mixtures with dielectric solid particles, in magnetic fields of between 5 and 9.4 T.^13^–^17^ These significant enhancements have allowed investigation of a range of systems such as functionalized porous materials,^9^,^18^–^21^ structural materials,^22^ polymers,^23^,^24^ nanoparticles,^9^,^21^,^25^,^26^ pharmaceuticals,^27^–^29^ and biomolecular structures,^30^–^41^ that were otherwise out of reach.

Under these conditions there are several mechanisms that might lead to polarization transfer,^12^,^42^ but currently the most efficient at 100 K is the cross effect (CE). The cross effect requires two dipolar coupled unpaired electrons to fulfill a condition where the difference in Larmor frequencies of the two electrons matches the Larmor frequency of the nucleus. There is currently much interest in improving the existing radicals to make cross-effect transfer more efficient. There have been a series of key steps to this end. The idea of using stable bi-radicals with limited flexibility fixes the inter-electron distance and leads to a large dipolar coupling, and was first realized in 2004 with the introduction of the BTnE^43^ biradicals and later with TOTAPOL.^44^ For nitroxide biradicals, the relative orientation of the two radicals is crucial since it defines the probability of matching the cross-effect condition between the two radical centers due to the anisotropy of the *g* tensor.^45^,^46^ As a result the bTbK biradical was introduced, in which the framework is rigid and the two TEMPO moieties, and therefore the corresponding *g* tensors are nearly orthogonal, the *g*_*xx*_ (or *g*_*yy*_) component of one TEMPO being nearly parallel to the *g*_*yy*_ (*g*_*zz*_) component of the other, which is the optimal orientation.^47^ The electron relaxation time is a further key property for DNP efficiency, and recently our group showed how dinitroxide biradicals with increased electron relaxation times give much higher DNP efficiency, and remained active at temperatures up to 200 K. Heavier, more bulky, radicals have longer electronic relaxation times, and we showed that this leads directly to better DNP with the introduction of bCTbK and TEKPol.^13^,^14^

Most of these bTbK-based radicals are not soluble in water. It was shown that surfactant-based micellar systems could be efficiently designed and used to solubilise these radicals in aqueous solvents.^48^,^49^ In 2013 Sauvée *et al.* introduced the inherently water-soluble urea-based PyPol and AMUPol bi-radicals, which also incorporate the concept of increased bulkiness.^17^ TEKPol and AMUPol, yielded previously unprecedented proton enhancements of over 200 at 9.4 T and 100 K in bulk solution.^14^,^15^

Herein we study a large series of bTurea, PyPol and bTbK derivatives designed specifically to establish the fine relationship between structural changes and DNP performance. We find that structural modifications of the radicals based on well-defined backbones can significantly modulate their DNP efficiency, and lead to sometimes significant increases in performance. One of the new radicals, TEKPol2 yields slightly higher enhancements than TEKPol, and as such is the best system to date. The present study suggests that in the bTbK series the limit on enhancement at 100 K and 9.4 T may now be primarily associated with other factors than the polarizing agents, such as microwave propagation in the sample.^16^

## Experimental

### NMR spectroscopy

All DNP experiments were carried out on a commercial Bruker Avance III 400 MHz NMR spectrometer equipped with a 263 GHz gyrotron microwave source using a 3.2 mm triple resonance MAS probe at sample temperatures around 100 K with spinning at 8 kHz.^50^ The sample temperature as a function of the microwave power was determined by measuring ^79^Br longitudinal relaxation times of crystalline KBr added in small amount to a 16 mM TEKPol/TCE : methanol-*d*_4_ (94 : 6 v/v) solution, and was assumed to be a good estimation of sample heating under microwave irradiation for all the other radical solutions.^16^,^51^ No correction was made to sample temperatures between microwave on and microwave off experiments. The microwave off temperature was around 105 K, and the microwave on temperature is expected to be between 110 and 115 K.^16^ The microwave power was optimized for each sample between 9 and 12 W to obtain the largest DNP enhancements. The magnet sweep coil was used to set the magnetic field so that microwave irradiation occurred at the maximum positive enhancement for a sample containing TOTAPOL. The ^1^H–^13^C cross-polarization^52^,^53^ (CP) DNP enhancements (*ε*_C CP_) were measured with a standard ramped CP pulse sequence.^54^ Since the ^1^H–^13^C CP signal is observed, *ε*_C CP_ corresponds to the proton enhancements of the frozen solution. In most cases *ε* was measured by comparing the intensity of the peaks acquired with microwave irradiation to that acquired without. In some cases integrated intensities were compared to determine *ε*_C CP_ in order to account for line narrowing arising from microwave induced sample heating. More details on the NMR parameters and spectra used for DNP enhancement measurements are given in the ESI.†

### NMR sample preparation

The biradicals were dissolved in 1,1,2,2-tetrachloroethane (TCE) to obtain bulk solutions of concentration around 16 mM. The samples were prepared by placing 24 μL of bulk biradical solution into a sapphire MAS rotor. Samples were topped with a silicone plug to prevent solution leakage from the rotors. In certain cases it was difficult to obtain a good glass with pure TCE, probably due to an interaction with the dissolved radical. Since 95/5 (v/v) solutions of dichloromethane and methanol have been reported to be good glass forming solutions,^55^ in some cases a small amount of fully deuterated methanol-*d*_4_ (*ca.* 4–6% by volume) was added to improve glass formation in the TCE solutions (see the ESI†).

### EPR spectroscopy

Experiments were conducted at W band (95 GHz) on a Bruker Elexsys E680 EPR spectrometer at 100 K on 16 mM flash-frozen solutions of radicals in 1,1,2,2-tetrachloroethane (TCE). The inversion-recovery times (*T*_ir_) were measured using an inversion-recovery sequence, and the data were fitted using a stretched exponential function, the reported value being the first moment of the distribution. The phase memory times (*T*_m_) were measured using a variable-delay Hahn-echo pulse sequence traced with a monoexponential function. Further details are given in the ESI.†

To match the DNP conditions the EPR spectra were recorded at high radical concentration (16 mM). In such conditions spin exchange and dipolar coupling strongly affect the measured values of *T*_ir_ and *T*_m_ which, as a result, do not correspond to the electronic longitudinal and transverse relaxation times *T*_1e_ and *T*_2e_, respectively. The measured values, however, are the most relevant to the present discussion because they match the DNP experimental conditions.

### Sample degassing and glass formation

DNP enhancements are significantly improved when samples are deoxygenated by a series of freeze–thaw (*i.e.* insert–eject) cycles performed inside the low temperature DNP probe.^16^ Longer nuclear *T*_1_ of the solvent leads to better DNP performance. This was also modelled theoretically by Hovav *et al.* for the static SE case.^56^ Each sample was cycled 8–12 times in this way. The samples were then inserted into the probe and *T*_1_ and ^1^H–^13^C CP DNP enhancement measurements as a function of microwave power were carried out.

### Synthesis of the radicals

Experimental details of synthetic procedures and characterization of all the new radicals are given in the ESI.† The urea-based dinitroxides **1–25** were prepared using the previously described procedure^17^ starting from either 4-amino tetramethyl or spirocyclohexyl or spirotetrahydropyranyl piperidinyloxy free radicals (see ESI†). Symmetrical and unsymmetrical urea-based dinitroxides were obtained by condensing the corresponding amines with triphosgene in dichloromethane. The TEKPol derivatives **28–38** were prepared according the procedure previously described for the synthesis of bTbK.^47^ Spirocyclohexyl or spirotetrahydropyrane piperidinyloxy precursors were obtained in moderate yields by crossed aldol condensation and Grob-type fragmentation of 1,2,2,6,6-pentamethylpiperidin-4-one, different ketones and ammonium chloride.^57^ Compounds **29** and **30** were obtained starting from the corresponding spirotetrahydrothiopyrane derivatives *via* desulfurization using RANEY® Ni.^58^

## Results and discussion

### General description of the investigated radicals

**Fig. 1:**
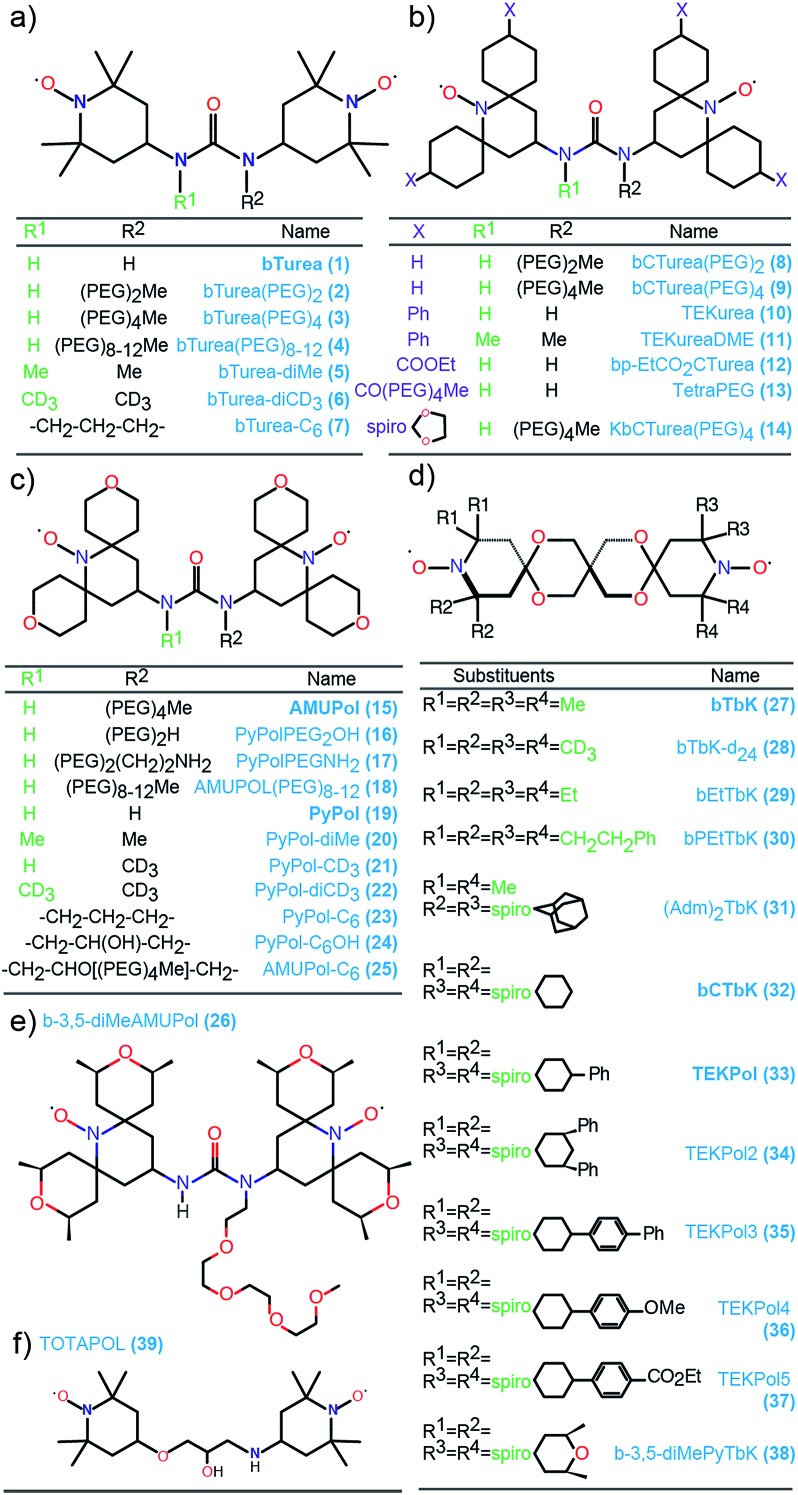
Structures and names of the radicals investigated in this study: (a) the bTurea series, (b) the bCTurea series, (c and e) the PyPol series, (d) the bTbK series. The TOTAPOL (f) and bCTbK (d) structures given for completeness. The prefix spiro indicates junction through just one carbon atom. PEG indicates a –CH_2_CH_2_O– unit. Molecular weights and synthetic routes are given in the ESI.†

Fig. 1 shows the molecular structures of the compounds investigated in this work. The structures are arranged according to their backbone. There are five different types of backbones which form the corresponding series, designated by the names of the simplest radical in which they are present: bTurea, bCTurea (only as derivatives), PyPol, and bTbK. For the purpose of clarity an additional TEKPol series was distinguished, which is based on the bTbK backbone with an additional spirocyclohexyl junction. In the urea-based radicals the main object of modifications was the substituents at the nitrogen atoms of the urea moiety (in the bTurea, bCTurea and PyPol series). The strategy involved *N*-methylation, introduction of PEG chains of different length or with different terminal groups, and constraining the geometry of the tether by using a *N*,*N*′-trimethyleneurea moiety. Furthermore, the substituents in α position to the nitroxide moiety were altered in all the series.

**Fig. 2:**
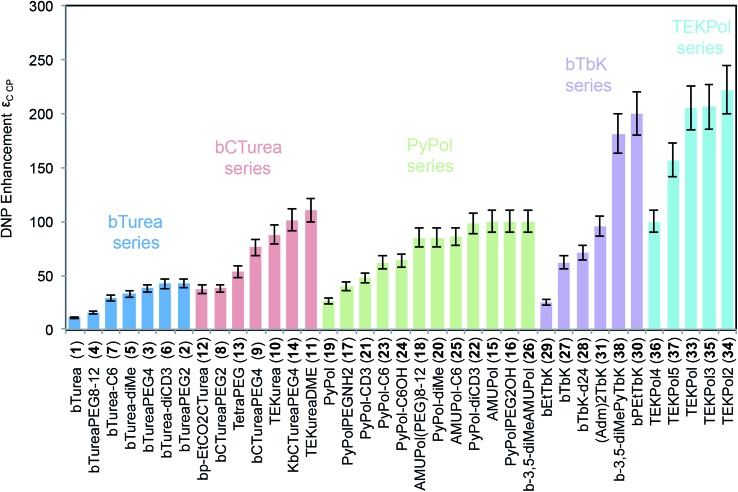
^1^H–^13^C CP DNP enhancement (*ε*_C CP_) for bulk solutions of TCE with 16 mM radical.

Fig. 2 shows the DNP enhancements factors (*ε*_C CP_) obtained for the NMR signal of TCE in frozen bulk 16 mM solutions of all 37 radicals studied. Each series is color-coded for convenience. Several main trends are immediately visible. For each series, modification of the substituents produces significant changes in enhancement, and for each group this spans at least a factor two. All the bTurea derivatives outperform bTurea. The radicals from the bCTurea series perform, on average, better than the bTurea derivatives. The PyPol series has on average similar performance to the bCTurea series. In the bTbK series two new radicals (**30** and **38**) yield markedly better enhancement than the other derivatives, due to longer relaxation times. The TEKPol series contains radicals yielding consistently the highest DNP enhancements among all the tested compounds.

### Correlation between electron and nuclear relaxation times, and enhancement

**Fig. 3:**
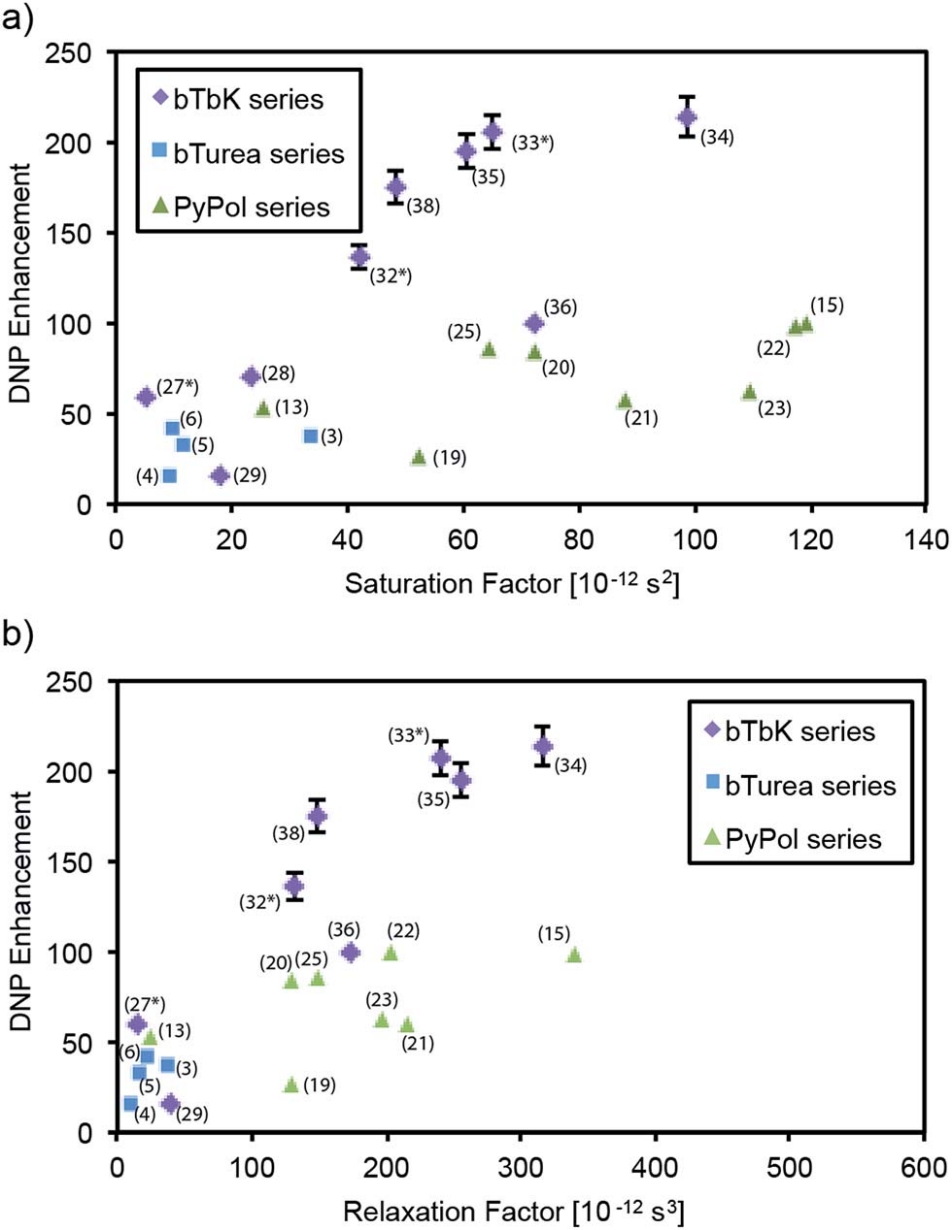
DNP enhancement (*ε*_C CP_) as a function of saturation factor (*T*_1e_·*T*_2e_) and relaxation factor (*T*_1e_·*T*_2e_·*T*_1n_) for selected compounds from (a and b) the bTurea series (TCE, 16 mM), (c and d) the PyPol series (TCE, 16 mM), (e and f) the bTbK series (TCE, 16 mM). The data points marked with an asterisk are taken from the previous study by Zagdoun *et al.*^14^ Error bars are shown where larger than the symbols.

Fig. 3 shows the DNP enhancement factors (*ε*_C CP_) of selected radicals as a function of their electron and nuclear spin relaxation parameters. The electron saturation factor *T*_1e_·*T*_2e_ governs the efficiency of the continuous wave (CW) saturation, and in the regime here we expect that the higher it is the more efficient saturation will be.^59^ We also introduce a factor, dubbed the “relaxation factor,” defined as *T*_1e_·*T*_2e_·*T*_1n_ which is a phenomenological parameter partially characterizing the efficiency of the cross effect by combining the saturation efficiency with the nuclear relaxation. Overall we see a clear correlation between both saturation and relaxation factors, and the enhancement. Looking within each series, in the bTurea series there is only a slight correlation between the observed enhancement and the measured saturation factor. The correlation with the relaxation factor is clearer. The PyPol series exhibits a correlation between high saturation factors and high enhancement. For the bTbK series there exists a clear correlation: the higher the saturation and relaxation factors, the higher the DNP enhancement.

For clarity, the discussion will be divided into two parts as a function of the core linker: (I) containing the bTurea, bCTurea and PyPol series and (II) the TEKPol series.

#### I. bTurea, bCTurea and PyPol urea-based series

The X-band EPR (9 GHz) spectra of the urea-based dinitroxides **1–26** in TCE solution at room temperature (ESI†) are characteristic of dinitroxides exhibiting spin exchange and nitrogen hyperfine coupling of the same order of magnitude (*A*_N_ ≈ *J* ≈ 45 MHz). At W-band (95 GHz) in frozen solution (100 K), the EPR spectra of **1–26** are mainly dominated by the large anisotropy of the *g* and ^14^N hyperfine tensors. The EPR data show that the molecular geometry of the dinitroxides in this series is similar, with an average distance between the two unpaired electrons of 11.5 Å, a calculated electron–electron dipolar coupling of 35 MHz and a spin exchange coupling of 30–55 MHz.

The PyPol derivatives are soluble both in organic solvents, such as TCE, and in water-based mixtures, such as glycerol/water. For the sake of comparison their DNP properties were studied here only in TCE. In glycerol/water PyPol and AMUPol exhibit higher *ε*_C CP_ (207 and 235, respectively, at 10 mM, 9.4 T and 100 K)^17^ than in TCE (*ε*_C CP_ of 26 and 100, respectively, at 16 mM, 9.4 T and 100 K). This observation can be explained by invoking slightly different conformations adopted by the radicals in the different solvents, changing the electron–electron dipolar coupling. We have calculated by molecular dynamics at 278 K (200 ns trajectories, using Gromacs 5.0.4 package) the average e–e distance *R*_ee_ and the average angle *θ* characterizing the relative orientation of the *g* tensors for a few bTurea derivatives (where *θ* is the angle between the COC planes of the two C–N(O)C moieties). For example for AMUPol we showed that these parameters are identical in water and in TCE. Another possibility to account for the difference in performance might be the formation of dinitroxide aggregates in TCE due to the tendency of urea moieties to interact through complementary hydrogen bonds.

##### Substitution on the Cα of the radical in the urea-based series (bTurea, bCTurea and PyPol)

Our previous study of the bTbK derivatives has shown that modification of the α position of the nitroxide moiety yields higher *ε*_C CP_ due to slower relaxation, with *T*_1e_ and *T*_2e_ electron relaxation times increased by a factor of 3 and 2, in bTbK and bPyTbK, respectively.^13^ Here, the same structural change has a similar effect on the *T*_1e_, which is 3 times longer in AMUPol (**15**) than in bTurea(PEG)_4_ (**3**). The *T*_2e_ is similar in both cases. This leads to an increase in *ε*_C CP_ from 38 to 100. An additional phenyl ring on the spirocyclohexyl moieties (TEKurea, **10**) causes an increase of *ε*_C CP_ from 11 to 88, as compared to bTurea (**1**). This observation is in line with higher *ε*_C CP_ obtained for longer electron relaxation times. The bCTurea series (**9**, **10**, **12** and **14**) illustrates this trend for the increasing size and rigidity of the group in the 4-position of the spirocyclohexyl moiety (–CO_2_Et < PEG chain < phenyl < dioxolane).

##### Modification of the linker in the urea-based series

The *N*-substituents of the urea moiety considerably affect the geometry of the molecule and can be used to modulate the urea-based intermolecular hydrogen-bonding network. This was the motivation to synthesize the *N*,*N*′-di, tri- and tetra-substituted urea dinitroxides. *N*,*N*′-Disubstituted ureas are commonly used in supramolecular chemistry as strong hydrogen-bond units in apolar solvents.^60^ The presence of H-donor and acceptor groups in bTurea (**1**) might possibly lead to the formation of clusters or aggregates. If this were to occur in the frozen samples, it would be detrimental to the DNP enhancement (a symptom would be poor glass formation).^61^ We find that replacing the hydrogen atoms of the ureide linker in bTurea (**1**) by two methyl groups (**5**) or a *N*,*N*′-trimethylene junction (**7**), leads to a factor 3 times higher enhancement. In the PyPol series, *ε*_C CP_ are 1.8, 2 and 3 times higher for PyPol-CD3 (**21**), PyPol-C6 (**23**) and PyPol-diMe (**20**), respectively, as compared to PyPol (**19**), in which two hydrogen atoms can act as H-bond donors. However, there is no clear correlation between the relaxation parameters of **19**, **20**, **21**, **23** and their DNP performance (Fig. 3), which indicates that other parameters, such as the orientation of the two TEMPO rings and/or the electron–electron dipole interactions, might be dominant. DFT calculations performed on PyPol (**19**) and PyPoldiMe (**20**) predict a significant difference in the structural parameters of their preferred conformations between TCE and water as solvent. In TCE, for PyPol (**19**): *R*_ee_ = 11.53 Å, dipolar coupling *d* = 34 MHz, *θ* = 56°; for PyPoldiMe (**20**): *R*_ee_ = 11.22 Å, dipolar coupling *d* = 37 MHz, *θ* = 89°. The MD calculations in water at 278 K gave the following results: for (**19**): *R*_ee_ = 11.6 ± 0.1 Å, *θ* = 26.9 ± 15°; for PyPoldiMe (**20**): *R*_ee_ = 11.4 ± 0.1 Å, *θ* = 71.7 ± 13.7°.

##### Effect of the PEG chain length

We introduced PEG chains on the nitrogen atom of the ureide linker in order to improve the solubility and to reduce the likelihood of the eventual formation of small aggregates. In both bTurea and PyPol series, the length of the PEG chain appears to have an effect on the DNP properties, with higher enhancements obtained for PEG chains containing 2 or 4 ethyleneoxy units (**2**, **3**, **8**, **9**, **15**) as compared to longer chains (8 to 10 units, **4**). The relaxation measurements indicate that long PEG chains actually decrease the electron relaxation times, probably by inducing local motions or local softening of the glass. We observed a similar effect for TetraPEG (**13**).

##### Effect of deuteration

It has been shown both theoretically and experimentally that electron spin *T*_m_ depends on proton concentration of the matrix below 70 K through the dipolar coupling between electrons and the bath of protons, and can be increased by deuteration of the solvent.^62^–^64^ The dipolar coupling is proportional to the magnetic moment, and so we expect that deuterium should provide less efficient relaxation than protons. Thus, even though the Raman relaxation is the dominating relaxation mechanism in nitroxides above 70 K, the deuteration of radicals is expected to slow down the electron relaxation times, yielding an increase in the DNP enhancement. Moreover, it may also affect the first steps of the polarization transfer from the electron to the nearby nuclei by changing the spin diffusion rate. It was shown that in the case of a non-spinning sample, protons in close proximity to the electron do not contribute to spin diffusion.^56^ This might not be the case under MAS since an adiabatic passage through the matching conditions might establish contact between the nearby protons and the proton bath, effectively allowing the spread of polarization even through the closest protons. Others have observed that deuteration of the solvent^65^,^66^ can affect DNP enhancements, whereas deuteration of the radical can slightly affect its electron relaxation properties.^67^ In particular, methyl groups are known to act as relaxation sinks and we expect them to have a strong influence on electron relaxation at 100 K.^63^ A comparison of the DNP and relaxation properties of protonated and deuterated bTurea-diMe (**5**, **6**) and PyPol-diMe (**20**, **22**) confirms this hypothesis. We observed a moderate increase in *ε*_C CP_ of around 27% and 15%, respectively. The *T*_1e_ and *T*_m_ of **22** were around 50% and 26% longer, respectively, than in **20**. However, this effect is accompanied by an increase in the DNP build-up time, which makes the deuteration approach unpractical.

#### II. bTbK and TEKPol series

The X-band EPR (9 GHz) spectra of the bTbK and TEKPol-based dinitroxides **27–38** in TCE solution at room temperature (ESI†) resemble that of a typical monomeric nitroxide-based radical with 3 lines, exhibiting a nitrogen hyperfine coupling *A*_N_ of 45 MHz. At W-band (95 GHz) in frozen solution (100 K), the EPR spectra of **27–38** are dominated by large anisotropy of the *g* and ^14^N hyperfine tensors, since the electron dipolar and exchange couplings are relatively small. The single crystal XRD measurements (where available) show that the distance and the relative orientation of the *g*-tensor of the two unpaired electrons is very similar throughout the series, mainly due to the rigidity of the tether that locks the two piperidinyl moieties in a nearly orthogonal orientation. We estimate the molecular geometry of the nitroxides **27–38** is similar, with an average distance between the two unpaired electrons of 11.9 Å, a calculated electron–electron dipolar coupling of 30 MHz and a very weak spin exchange coupling (<5 MHz).

In our previous work we established that the saturation factor (*T*_1e_·*T*_2e_) of the electronic transition is highly correlated with the DNP performance in a series of dinitroxides exhibiting similar *g* and hyperfine tensors, and electron–electron (or dipole and spin exchange) couplings. This result led to the introduction of TEKPol (**33**) which yields proton DNP enhancements of over 200 at 9.4 T and 100 K, and has been the most efficient polarizing agent in organic solvents (TCE) so far. In order to improve our understanding of the parameters driving the CE polarization in dinitroxides, we synthesized and investigated new TEKPol derivatives. We introduced additional groups onto the spirocyclohexyl rings intended to suppress the librational modes (**35**, **36**, **37**), and changed the substituents in the α position of the nitroxide (**28**, **29**, **30**, **31**, **34**, **38**) in order to target an optimum saturation factor and to obtain information on the shielding effect on the solvent molecules by sterically hindering the unpaired electron. Table 1 summarizes the quenching factors, enhancement and overall sensitivity enhancements for selected radicals investigated in this study.

**Table 1:** Quenching factors, ^1^H *T*_DNP_, DNP enhancements (*ε*_C CP_), and overall sensitivity enhancements *Σ*_C CP_ and *Σ*†C CP for selected biradicals in bulk TCE solutions^*e*^

Sample	Quenching (1 – *θ*)^*a*^	*ε*_C CP_	^1^H *T*_DNP_	*Σ*_C CP_ ^*b*^	*Σ* † C CP ^*c*^
PyPol (**19**)	0.30 ± 0.03	26 ± 3	3.2	72 ± 13	201 ± 36
bTbK (**27**)	0.48 ± 0.05	62 ± 6	2.6	141 ± 24	395 ± 67
bCTbK (**32**)	0.55 ± 0.06	93 ± 9	3.0	170 ± 28	477 ± 78
TEKPol (**33**)	0.65 ± 0.07	205 ± 21	3.0	290 ± 49	812 ± 137
TEKPol2 (**34**)^*d*^	0.49 ± 0.05	155 ± 16	3.4	310 ± 54	868 ± 151

^*a*^ *θ* is the fraction of NMR signal observed in the sample doped with radicals compared to pure TCE without radicals.^68^

^*b*^ *Σ*_C CP_ accounts for quenching and the change of relaxation rates between regular low temperature NMR and in the presence of a radical.

^*c*^ *Σ* † C CP additionally accounts for the Boltzmann temperature factor.

^*d*^Measurement performed on a different DNP spectrometer, where the radical was not fully saturated by microwaves even at the highest available MW power, leading to reduced *ε*_C CP_.

^*e*^Factor between a low temperature (100 K) and a room temperature (298 K) experiment.^68^^1^H *T*TCE1, the value for the pure degassed solvent without radical, was measured to be 50.0 s. The errors in *ε*_C CP_, *Σ*_C CP_, *Σ*†C CP are estimated to be about 10%.

The quenching factor is the fraction of nuclei in the sample which do not contribute to the observable signal.^68^ Quenching factors were measured as described in the ESI.† Another process leading to a decrease in the signal intensity in MAS-DNP experiments is depolarization arising from sample spinning.^69^–^71^ It is thus always essential to quantify the net sensitivity enhancement due to DNP. The overall sensitivity enhancement *Σ*_C CP_ is the overall gain in sensitivity, accounting for quenching and faster relaxation due to the presence of a radical, relative to a regular low temperature NMR experiment, and in the case of *Σ*†C CP accounting also for the Boltzmann factor between a low-temperature DNP and a room-temperature NMR experiment.^68^ Here we see that quenching factors increase as we move from the radicals with shorter to longer electron relaxation times, as might be expected.^69^

##### Effect of deuteration

As discussed previously, deuteration of nuclei nearby the radical center is expected to improve DNP performance. Furthermore, for nitroxide free radicals at temperatures around 60–140 K, *T*_m_ is largely determined by the dynamics of the nearby methyl groups in the α position.^72^ In order to evaluate this effect on the DNP properties, we prepared a version of bTbK (bTbK-d_24_, **28**) with deuteration at the methyl groups and preservation of protons on the methylene of the piperidine rings. The saturation factor of bTbK-d_24_ (**28**) is 1.5 times higher than that of bTbK (**27**), while *ε*_C CP_ and the build-up time increased by factors of 1.2 and 1.8, respectively.

##### Modification of the spirocyclohexyl moieties

In order to increase the saturation factor of the polarizing agents, we have prepared a series of TEKPol derivatives of higher molecular weight by introducing additional groups onto the spirocyclohexyl rings. With respect to TEKPol (**33**), TEKPol2 (**34**) and TEKPol3 (**35**) have one additional phenyl ring on the spirocyclohexyl moieties, and they are regioisomers. It is interesting to note that in **35** the introduction of the phenyl ring in the *para* position of the phenyl in TEKPol does not improve the saturation factor (we rather observed a reduction of around 10%), while in **34** the introduction of two phenyl rings into the 3,5 position on the spirocyclohexyl moieties increases the saturation factor by 50%. As expected, this change in the relaxation properties affects the DNP enhancement and TEKPol2 (**34**) yields the highest *ε*_C CP_ obtained so far under these conditions (222 ± 22). The DNP enhancement of TEKPol2 (**34**) was measured at both 5 mM and 10 mM concentration but it was lower than at 16 mM (47 ± 5 and 119 ± 12, respectively). Note that the introduction of a methoxy (**36**) or an ethoxycarbonyl (**37**) group into the phenyl ring of TEKPol reduces *ε*_C CP_ by 50% or 23%, respectively, and the saturation factor of **36** is reduced by 50%. This result cannot be explained simply by the presence of fast-relaxing protons of the methyl groups since the DNP enhancement of **38** is still high. This observation underlines the difficulty of tuning the substituents involved in the first step of the polarization transfer inside the proton inner sphere.

##### Modification of crowding around the unpaired electron

Recently it has been reported that the introduction of ethyl groups in the α position of the nitroxide sterically shields the unpaired electron and prevents its reduction by ascorbate.^58^ In order to understand the interaction of the solvent molecules with the unpaired electron we have synthesized a bTbK derivative (**29**) containing ethyl groups. The saturation factor of bEtTbK (**29**) is about 3 times higher than the one of bTbK,^13^ but against expectations the DNP enhancement is two times lower (Fig. 2 and 3). This result was confirmed on two different preparations of the radical, and we currently do not have an explanation for this low performance. It may be related to a problem with the samples, or to the properties that lead to the radical shielding mentioned above.^58^ However, a sample with phenylethylene moieties in the α position (bPEtTbK, **30**) yields a DNP enhancement similar to the value obtained for TEKPol (**33**) and TEKPol2 (**34**). In order to obtain a very rigid and bulky bTbK analogue, we also introduced two adamantyl groups into the α position (**31**). In this case *ε*_C CP_ is 55% higher than for bTbK.

## Conclusions

In conclusion we have prepared a series of 37 dinitroxide biradicals and studied their performance as polarizing agents in cross-effect DNP experiments at 9.4 T and 100 K in TCE. We clearly confirm that in this regime the DNP performance is strongly correlated with the electron and nuclear spin relaxation times, with longer relaxation times leading to better enhancements. We also observe that deuteration of the radicals generally leads to better DNP enhancement at the expense of longer build-up time.

We note that better performance is here usually associated with larger molecules. The size of the radicals may also have consequences in different applications: for example larger radicals may not enter the pores of porous materials, which may lead on the one hand to lower signal enhancements but also on the other hand to less signal bleaching,^73^ and to avoidance of possible reactivity between the radical and the substrate. Thus, the size range of the radicals may also itself be an interesting and useful parameter to exploit in these radical families.

It is interesting to note that adding further substituents to TEKPol does not produce significant improvement seen as we go from bTbK to bCTbK to TEKPol. We hypothesize that it may be that at this point the optimum relaxation parameters have been reached in the bTbK series and so the limit of performance for these series of radicals at 9.4 T and 100 K. Notably it has recently been shown that in samples where microwave propagation is improved by incorporating dielectric particles, TEKPol yields enhancements of over 500, so that we consider that under optimum microwave irradiation TEKPol and TEKPol2 approach the theoretical limit. Even though the bulkier TEKPol derivatives may have better relaxation properties, they do not produce significantly better enhancements. To increase enhancements it may be necessary to act on other parameters. Having said this, we note that the new radical TEKPol2 introduced here does slightly outperform the others, and provides the best performance so far under these conditions. It would be particularly interesting to see how the performance measured here at 9.4 T translates to higher fields and/or higher temperatures, and such studies are now underway.

## Supplementary Material

Supplementary information
