# Supplementary material for: Rational design of dinitroxide biradicals for efficient cross-effect dynamic nuclear polarization
Source: Chem Sci. 2015 Oct 13;7(1):550–8. doi: 10.1039/c5sc02921j (PMC5952883; doi:10.1039/c5sc02921j)
Supplement: Supplementary file 1 [file SC-007-C5SC02921J-s001.pdf]

Electronic Supplementary Material (ESI) for Chemical Science.
This journal is © The Royal Society of Chemistry 2015

Supporting information for: Rational design of dinitroxide biradicals for efficient cross-effect dynamic nuclear polarization

Dominik J. Kubicki,[†a] Gilles Casano,[†c] Martin Schwarzwälder,[d] Sébastien Abel,[c] Claire Sauvée,[c] Karthikeyan Ganesan,[c] Maxim Yulikov,[d] Aaron J. Rossini,[a] Gunnar Jeschke,[d] Christophe Copéret,[d] Anne Lesage,[b] Paul Tordo,*[c] Olivier Ouari,*[c] and Lyndon Emsley.*[a]

†These authors contributed equally to this work.

[a]*Institut des Sciences et Ingénierie Chimiques, Ecole Polytechnique Fédérale de Lausanne (EPFL), CH-1015 Lausanne, Switzerland.*

[b]*Université de Lyon, Institut de Sciences Analytiques (CNRS / ENS de Lyon / UCB-Lyon 1), Centre de RMN à Très Hauts Champs, 69100 Villeurbanne, France.*

[c]*Aix Marseille Université, CNRS, ICR UMR 7273, 13397 Marseille, France.*

[d]*ETH Zurich, Departament of Chemistry, Laboratory of Inorganic Chemistry, 8093 Zurich, Switzerland.*

Table of contents

Sample Compositions for DNP Experiments and recorded $T_{DNP}(^1H)$ 2

Acquisition parameters 3

MAS-DNP NMR spectra 4

EPR measurements 6

Quenching factor measurements 8

Synthesis of reported molecules 9

Description of the synthetic schemes 9

Synthetic route to bTurea-diMe (5), bTurea-diCD$_3$ (6), PyPol-DiMe (20), PyPol-diCD$_3$ (22), TEKurea (10), TEKurea-diMe (11) and PyPol-CD$_3$ (21) 9

Synthetic route to b*p*-EtCO2CTurea (12) and TetraPEG (13) 10

Synthetic route to KbCTurea(PEG)$_4$ (14) 10

Synthetic route to PyPolPEGNH$_2$ (17) 11

General synthetic route to b-3,5-diMePyTbK(38), TEKPol2 (34), TEKPol3 (35), TEKPol4 (36), TEKPol5 (37), (Adm)$_2$TbK (31), bEtTbk (29), bPEtTbK (30) and bTbK-d$_{24}$ (28) 12

Synthetic route to b-3,5-diMeAMUPol (26) 14

Experimental Section 15

EPR spectra of reported dinitroxides 41

Sample Compositions for DNP Experiments and recorded $T_{DNP}(^1H)$

The concentration of radical solutions was (16.0 ± 1.0) mM. In each case around 24 µL of solution was placed in a rotor.

Table S1. Sample Compositions for DNP Experiments and Maximum (after degassing) DNP Build-up Times $T_{DNP}(^1H)$.

Number[a]	Radical	Solvent	Maximum $T_{DNP}(^1H)$ / s [b]
**1**	bTurea	TCE	1.2
**2**	bTurea(PEG)$_2$	TCE	1.4
**3**	bTurea(PEG)$_4$	TCE	1.1
**4**	bTurea(PEG)$_{8\text{-}12}$	TCE	1.2
**5**	bTurea-diMe	TCE	1.5
**6**	bTurea-diCD$_3$	TCE	2.3
**7**	bTurea-C$_6$	TCE	1.2
**8**	bCTurea(PEG)$_2$	TCE	1.5
**9**	bCTurea(PEG)$_4$	TCE	1.4
**10**	TEKurea	TCE	1.5
**11**	TEKureaDME	TCE	1.5
**12**	bp-EtCO$_2$CTurea	TCE	1.0
**13**	TetraPEG	TCE	1.0
**14**	KbCTurea(PEG)$_4$	TCE	0.8
**15**	AMUPol	TCE	1.7
**16**	PyPolPEG$_2$OH	TCE	2.0
**17**	PyPolPEGNH$_2$	TCE	2.9
**18**	AMUPol(PEG)$_{8\text{-}12}$	TCE	1.8
**19**	PyPol	TCE	2.5
**20**	PyPol-diMe	TCE	1.8
**21**	PyPol-CD$_3$	TCE	2.4
**22**	PyPol-diCD$_3$	TCE/methanol-d_4 (93:7 v/v)	2.9
**23**	PyPol-C$_6$	TCE	1.8
**24**	PyPol-C$_6$OH	TCE	2.8
**25**	AMUPol-C$_6$	TCE	2.3
**26**	b-3,5-diMeAMUPol	TCE	1.8
**27**	bTbK	TCE/methanol-d_4 (93:7 v/v)	2.6
**28**	bTbK-d$_{24}$	TCE	4.8
**29**	bEtTbK	TCE	2.2
**30**	bPEtTbK	TCE	3.7

**31**	$(Adm)_2TbK$	TCE	3.4
**33**	TEKPol	TCE	3.2
**34**	TEKPol2	TCE	3.1
**35**	TEKPol3	TCE	4.2
**36**	TEKPol4	TCE	2.4
**37**	TEKPol5	TCE	3.1
**38**	b-3,5-diMePyTbK	TCE	3.8

[a]cf. Fig. 1 in the main text. [b]The error of the ^{1}H T_{DNP} fit was smaller than 1%.

Acquisition parameters

Table S2. Acquisition parameters

Pulse Sequence	CP-MAS
Nucleus	^{13}C
Number of scans	8-64
Recycle Delay (s)	4.0
Dwell (us)	12-17
Spectral width (kHz)	30-41
Spinning speed (kHz)	8.0
Number of points	888-2048
^{1}H π pulse length (us)	4.8-5.0
Contact pulse length (us)	750
1H rf field during contact pulse (kHz)	85-120
X rf field during contact pulse (kHz)	60-72
^{1}H rf field during SPINAL-64 decoupling (kHz)	85-120

MAS-DNP NMR spectra

The uncertainty of $\varepsilon_{C\ CP}$ was evaluated to be 10% using the S/N ratio of the spectra in all cases.

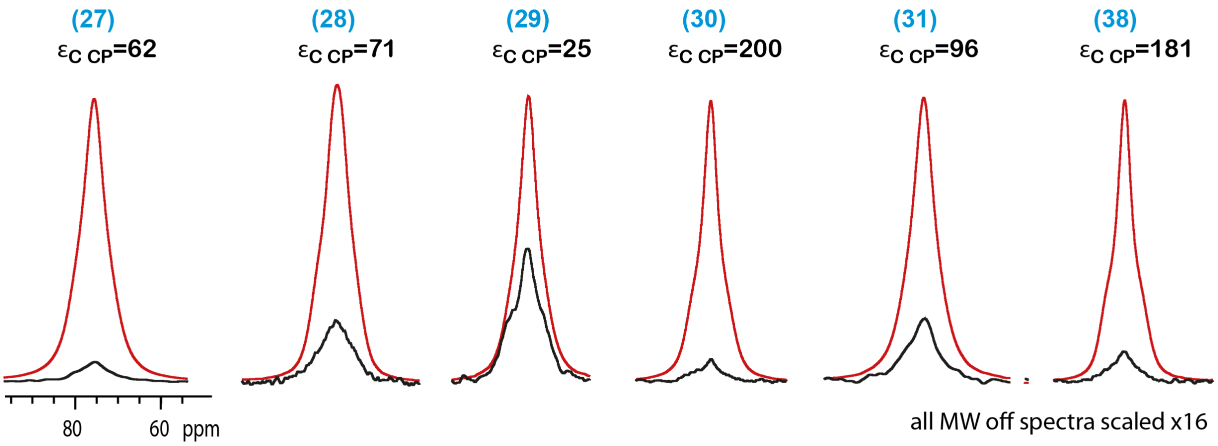

Figure S1. Spectra used to make Figure 2 of the main text (the bTurea series). The number at the top of each spectrum corresponds to the numbering in Figure 1 in the main text. Both ^{13}C CPMAS spectra acquired with (top spectrum, in red) and without (bottom spectrum, in black) microwave irradiation are shown and the determined value of $\varepsilon_{C\ CP}$ is given. Number of scans: 8-32 for microwave on and 16-64 for microwave off spectra. For other acquisition parameters see Table S2.

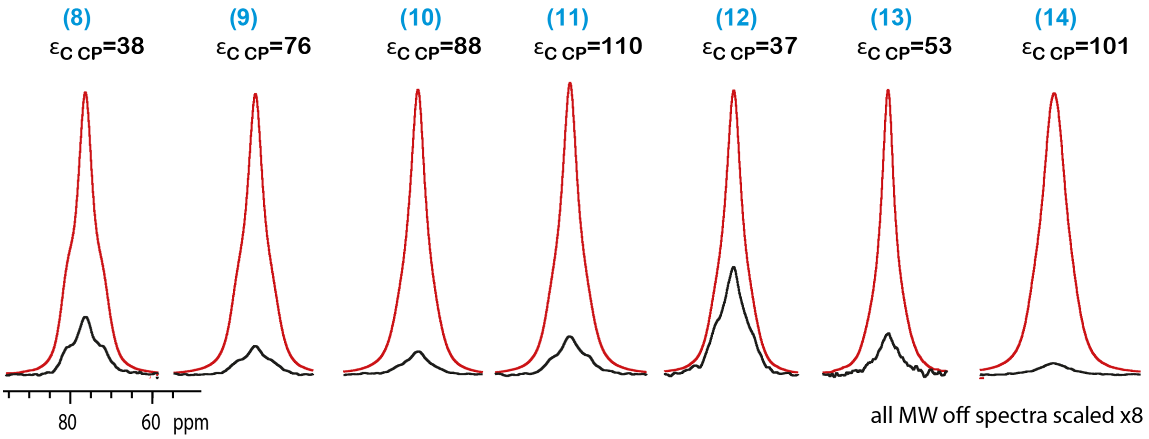

Figure S2. Spectra used to make Figure 2 of the main text (the bCTurea series). The number at the top of each spectrum corresponds to the numbering in Figure 1 in the main text. Both ^{13}C CPMAS spectra acquired with (top spectrum, in red) and without (bottom spectrum, in black) microwave irradiation are shown and the determined value of $\varepsilon_{C\ CP}$ is given. Number of scans: 8-32 for microwave on and 16-64 for microwave off spectra. For other acquisition parameters see Table S2.

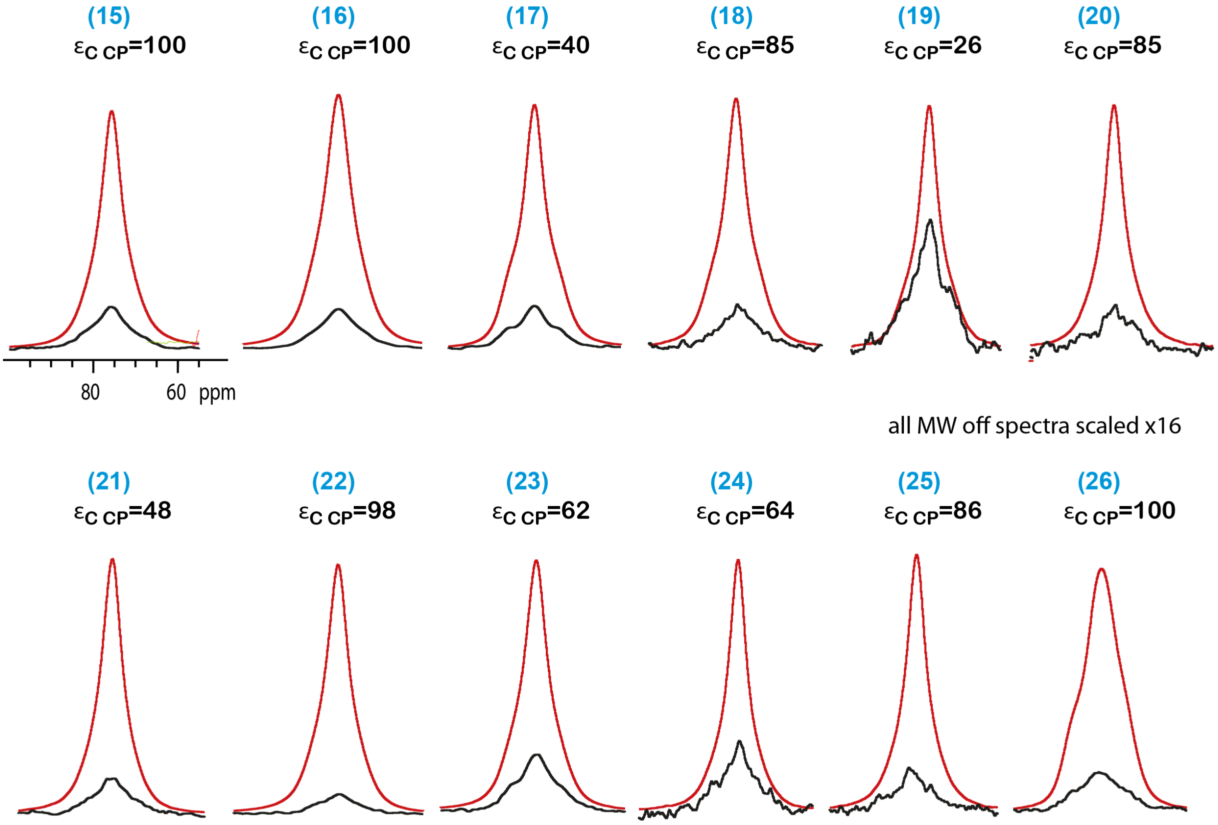

Figure S3. Spectra used to make Figure 2 of the main text (the PyPol series). The number at the top of each spectrum corresponds to the numbering in Figure 1 in the main text. Both ^{13}C CPMAS spectra acquired with (top spectrum, in red) and without (bottom spectrum, in black) microwave irradiation are shown and the determined value of $\varepsilon_{C\,CP}$ is given. Number of scans: 8-32 for microwave on and 16-64 for microwave off spectra. For other acquisition parameters see Table S2.

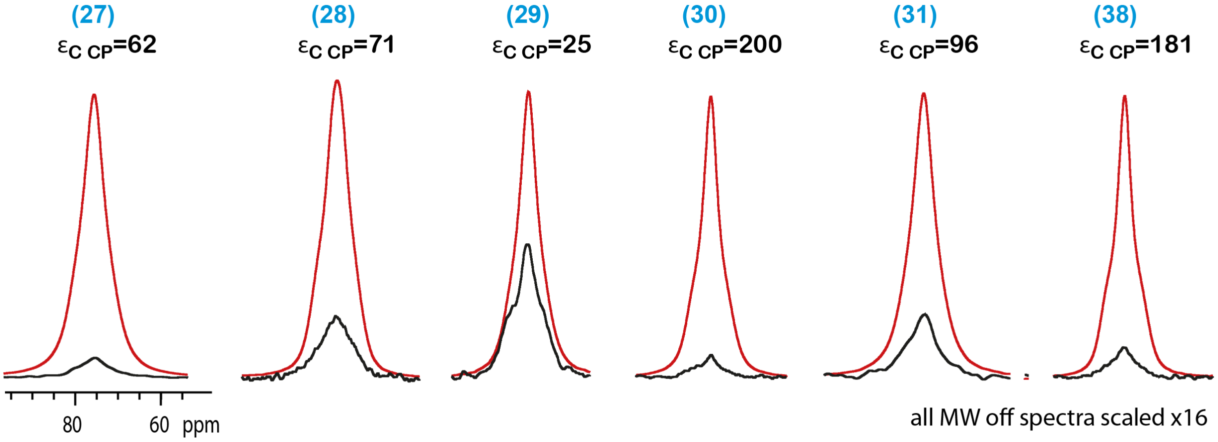

Figure S4. Spectra used to make Figure 2 of the main text (the bTbK series). The number at the top of each spectrum corresponds to the numbering in Figure 1 in the main text. Both ^{13}C CPMAS spectra acquired with (top spectrum, in red) and without (bottom spectrum, in black) microwave irradiation are shown and the determined value of $\varepsilon_{C\,CP}$ is given. Number of scans: 8-32 for microwave on and 16-64 for microwave off spectra. For other acquisition parameters see Table S2.

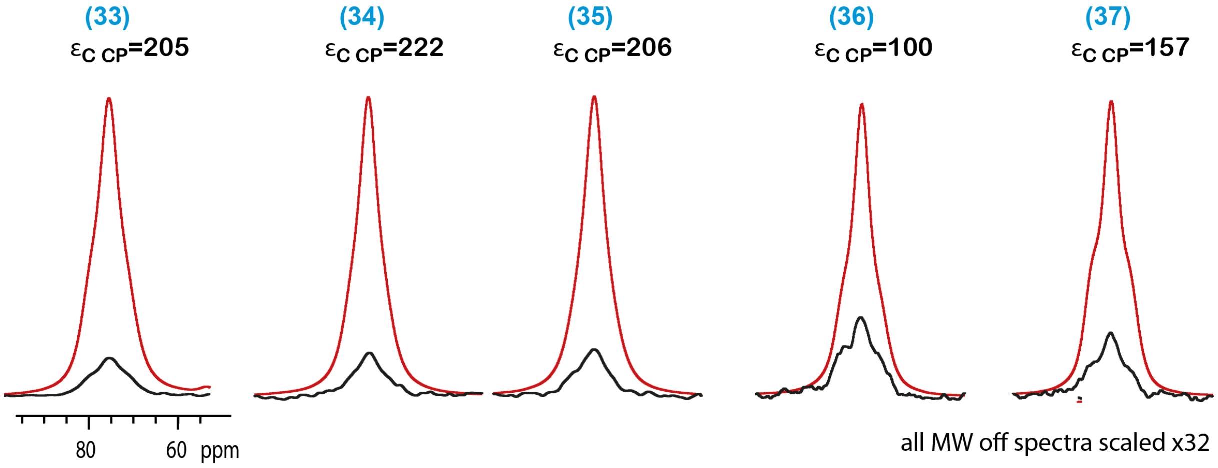

Figure S5. Spectra used to make Figure 2 of the main text (the TEKPol series). The number at the top of each spectrum corresponds to the numbering in Figure 1 in the main text. Both ^{13}C CPMAS spectra acquired with (top spectrum, in red) and without (bottom spectrum, in black) microwave irradiation are shown and the determined value of $\varepsilon_{C\ CP}$ is given. Number of scans: 8-32 for microwave on and 16-64 for microwave off spectra. For other acquisition parameters see Table S2.

EPR measurements

All pulse experiments were recorded at W band (94 GHz) on a Bruker Elexsys E680 EPR spectrometer. The temperature was stabilized with an Oxford helium flow cryostat and was held at 100 K. The longitudinal relaxation time T_{1e} was determined performing an inversion-recovery experiment. The used pulse sequence is shown in Fig. S6. Pulse lengths used were 240 ns for the inversion pulse, 100 ns for the $\pi/2$ and 200 ns for the refocusing π pulse. The initial d_{var} was set to 2000 ns and the time increment was varied depending on the relaxation time between 352 up to 3900 ns. 1024 points were recorded for each trace. The 2-pulse echo delay d was 212 ns. Error bars are estimated to be on the order of 1% as good signal to noise spectra were recorded for all samples. The concentration of the radical solutions was (16.0 ± 0.5) mM. The solvent was 1,1,2,2-tetrachloroethane (TCE).

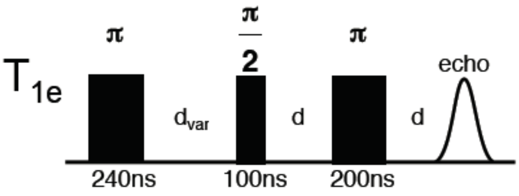

Figure S6. Pulse sequence applied in pulse EPR for inversion-recovery experiments to determine T_{1e}.

The inversion-recovery time traces were fitted using a stretched exponential function:

$$I(t) = I_0 + I_1 exp\left(-(t/T_{1e}^*)^\beta\right)$$

where I_0 is the initial intensity, I_1 the proportionality factor, T_{1e}* the decay time parameter and *β* the stretching parameter.

The first moment <T_{1e}> of the distribution is considered to be the mean relaxation time, called the inversion-recovery time T_{ir} and is given by:

$$\langle T_{1e} \rangle \equiv \int_0^\infty exp\left(-(x/T_{1e}^*)^\beta\right) dt = \frac{T_{1e}^*}{\beta}\Gamma\left(\frac{1}{\beta}\right)$$

The phase memory time T_m was determined using a variable-delay Hahn-echo pulse sequence. The used pulse sequence is shown in Fig. S7. Pulse lengths used were 100 ns for the $\pi/2$ and 200 ns for the refocusing π pulse. The initial d_{var} was set to 344 ns, the time increment was varied depending on the relaxation time between 4 up to 16 ns. 800 points were recorded for each trace. The 2-pulse echo decay traces were fitted using a monoexponential function. Error bars are estimated to be on the order of 1% as good signal to noise spectra were recorded for all samples.

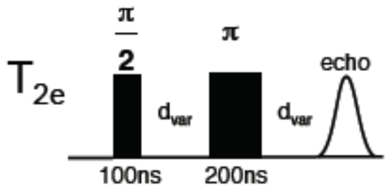

Figure S7. Pulse sequence applied in pulse EPR for 2-pulse Hahn-echo decay experiments to determine T_m.

Table S3. Summary of the recorded electron relaxation properties of the biradicals.

Number[a]	Radical	T_{1e}^* / µs	β	T_{ir} / µs	T_m / ns	Saturation factor / $\cdot 10^{12}$ s^2	^1H T^1 / s	Relaxation factor / $\cdot 10^{12}$ s^3
**3**	bTurea-PEG4	12	0.44	31	1082	34	1.1	37
**4**	bTurea-PEG8-12	8	0.46	19	474	9	1.2	11
**5**	bTureadiMe	13	0.46	30	375	11	1.5	17
**6**	bTureadiCD3	13	0.46	29	326	10	2.3	22
**13**	TetraPEG	17	0.42	48	528	26	1.0	26
**15**	AMUPOL	59	0.55	99	1202	119	1.7	203
**19**	PyPol	26	0.54	45	1160	52	2.5	130
**20**	PyPoldiMe	30	0.50	61	1181	72	1.8	130
**21**	PyPolCD3	50	0.53	91	961	88	2.4	211
**22**	PyPoldiCD3	45	0.54	79	1487	117	2.9	340
**23**	PyPol C6	36	0.50	70	1562	109	1.8	197
**25**	AMUPOL C6	26	0.48	56	1144	64	2.3	148
**28**	bTbK-d24	24	0.56	40	578	23	4.6	107

**29**	bEtTbK	28	0.60	42	433	18	2.2	40
**34**	TEKPol2	48	0.55	81	1210	99	3.2	315
**35**	TEKPol3	41	0.59	63	957	61	4.2	255
**36**	TEKPol4	35	0.52	66	1105	73	2.4	174
**38**	b-3,5-diMEPytbK	43	0.50	86	563	48	3.1	149
**27**[b]	bTbK (14.7 mM)	2	0.36	10	588	6	2.6	15
**32**[b]	bCTbK (13.3 mM)	18	0.49	37	1128	42	3.2	134
**33**[b]	TEKPol (13.0 mM)	21	0.47	46	1416	65	3.7	241

[a]cf. Fig. 1 in the main text.
[b] These data points come from a previous work by Zagdoun et al. (A. Zagdoun, G. Casano, O. Ouari, M. Schwarzwälder, A. J. Rossini, F. Aussenac, Y. M., J. G., C. Copéret, A. Lesage, P. Tordo and L. Emsley, *J. Am. Chem. Soc.*, 2013, 135, 12790-12797)

Quenching factor measurements

The quenching measurements were performed similarly to the procedure described previously.[a] The fraction of NMR signal observed in a sample doped with a radical compared to pure TCE is denoted as θ. The relaxation delay D1 was set to be at least $5 \cdot T_1$ in order to allow complete relaxation. The solvent integral was normalized by mass and by number of scans. Accordingly the quenching factor 1-θ is the fraction of nuclei in the sample which do not contribute to the observable signal. The overall sensitivity enhancement $\Sigma_{C\,CP}$ was calculated according to the following formula:

$$\Sigma_{C\,CP} = \varepsilon_{C\,CP} \cdot \theta \cdot \sqrt{\frac{T_{1(pure\ TCE)}}{T_{DNP(radical\ solution)}}} = \varepsilon_{C\,CP} \cdot \theta \cdot \sqrt{\kappa}$$

With respect to room temperature NMR experiment, the overall sensitivity enhancement including the Boltzmann factor $\Sigma^{\dagger}_{C\,CP}$ reads:

$$\Sigma_{C\,CP}{}^{\dagger} = \frac{298\ K}{105\ K} \cdot \varepsilon_{C\,CP} \cdot \theta \cdot \sqrt{\kappa} = 2.8\sqrt{\kappa}$$

All solutions were 16.0 mM (± 0.5 mM).

[b]	Sample	Conc. / mM	ns[e]	Mass of solution / mg	^{1}H T_{DNP} / s	D1[f] / s	$\varepsilon_{C\,CP}$	Seen signal θ / %	Quenched signal (1-θ) / %	κ	$\Sigma_{C\,CP}$	$\Sigma^{\dagger}_{C\,CP}$
-	pure TCE	-	64	32.9	50±10[c]	300	-	100	0	-	-	-
**19**	PyPol	16.0	64	32.5	3.2	20	26±3	70±7	30±3	16±3	72±13	201±36
**27**	bTbK	16.1	128	33.6	2.6	20	62±6	52±5	48±5	19±4	141±24	395±67
**32**	bCTbK	15.8	128	31	3.0	20	93±9	45±5	55±6	17±3	170±28	477±78
**33**	TEKPol	16.3	128	30.5	3.0	20	205±21	35±4	65±7	17±3	290±49	812±137
**34**	TEKPol2	16.0	16	35.5	3.3	17	155±16[d]	51±5	49±5	15±3	310±54	868±151

[a]A. J. Rossini, A. Zagdoun, M. Lelli, D. Gajan, F. Rascon, M. Rosay, W. E. Maas, C. Copéret, A. Lesage and L. Emsley, *Chemical Science*, 2012, 3, 108-115.
[b]cf. Fig. 1 in the main text.
[c] ^{1}H T_1. The value for the degassed sample varied depending on the glass quality, hence the large error associated with it. The error of ^{1}H T_{DNP} fit was smaller than 1%.
[d]Measurement performed on a different DNP system where the radical was not saturated with microwaves even at the highest available MW power.
[e]Number of scans
[f]Relaxation delay

The errors of $\varepsilon_{C\,CP}$ and θ were evaluated to be 10% by taking the S/N ratio of spectra. The errors of κ, $\Sigma_{C\,CP}$, $\Sigma^{\dagger}_{C\,CP}$ were calculated using the propagation of error.

Synthesis of reported molecules

Description of the synthetic schemes

Synthetic route to bTurea-diMe (5), bTurea-diCD$_3$ (6), PyPol-DiMe (20), PyPol-diCD$_3$ (22), TEKurea (10), TEKurea-diMe (11) and PyPol-CD$_3$ (21)

bTurea-diMe (**5**), bTurea-diCD$_3$ (**6**), PyPol-DiMe (**20**), PyPol-diCD$_3$ (**22**), TEKurea (**10**), TEKurea-diMe (**11**) were synthesized in a two-steps sequence starting from ketones **40**, **43** and **46**. First, amino derivatives **41**, **44**, **48**, amino derivatives **42**, **45** and amino compound **47** were prepared by reacting ketone **40**, **43** and **46** with methylamine hydrochloride, methylamine-D$_3$-hydrochloride or ammonium acetate in the presence of sodium cyanoborohydride. Then, the compounds **41**, **42**, **44**, **45**, **47** and **48** were then reacted with triphosgene to yield dinitroxides bTurea-diMe (**5**), bTurea-diCD$_3$ (**6**), PyPol-DiMe (**20**), PyPol-diCD$_3$ (**22**), TEKurea (**10**), TEKurea-diMe (**11**) respectively. PyPol-CD$_3$ (**21**) was obtained by condensation of amino compound **49** and methyl-D$_3$-amine **45** in the presence of triphosgene.

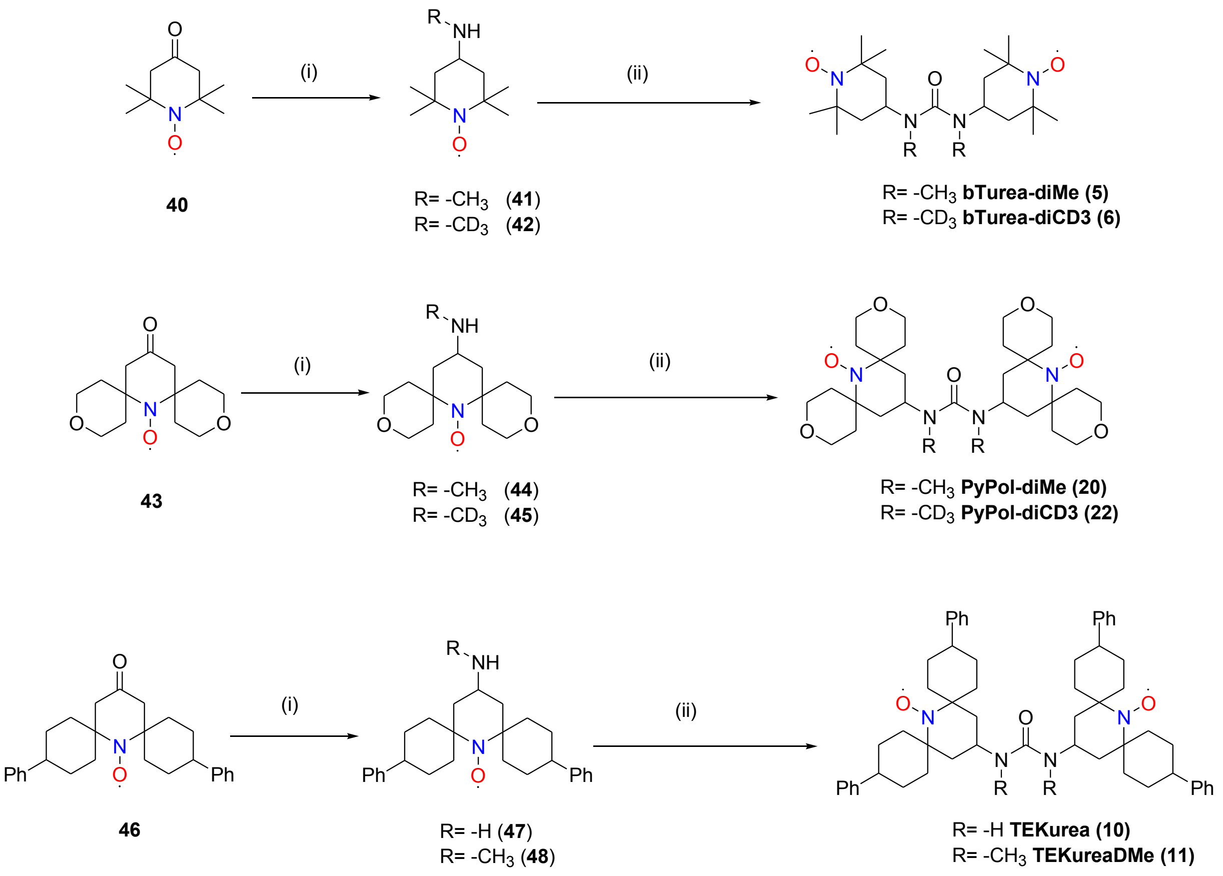

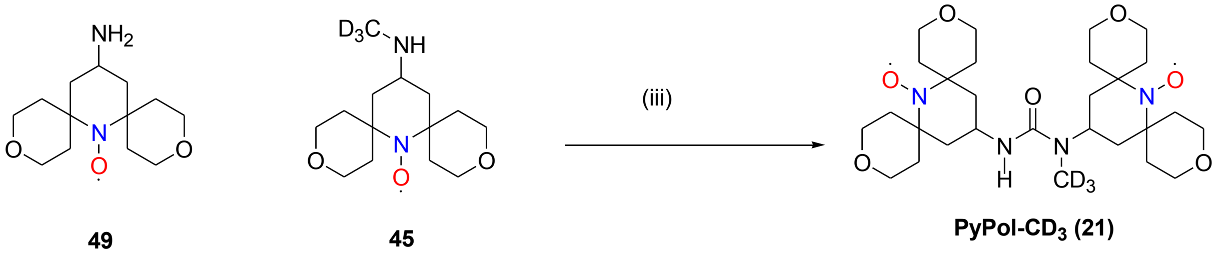

SI-Scheme 1: synthetic route to dinitroxides 5, 6, 10, 11, 20, 21, 22. Reagents and conditions: (i) RNH_2.HCl or $AcONH_4$, $NaBH_3CN$, MeOH, 25°C. (ii) Triphosgene, TEA, CH_2Cl_2, reflux, 6h. (iii) Triphosgene, TEA, CH_2Cl_2, 25°C, 12h.

Synthetic route to b*p*-EtCO2CTurea (12) and TetraPEG (13)

The spirocyclohexyl moieties were introduced according the method of Yamada et al.[1] starting from ethyl 4-cyclohexanonecarboxylate and amine **50**. The resulting 2,6-substitued piperidin-4-one **51** was oxidized with hydrogen peroxide in the presence of sodium tungstate to afford **52**. Reductive amination of ketone **52** using sodium borohydride and ammonium acetate yielded to amine **53** that was further reacted with triphosgene to obtain b*p*-EtCO_2CTurea (**12**). Then, the hydrolysis of the four ethylester functions gave compound **54**. Finally, tetra(ethylene glycol)methyl ether was coupled at tetraacid **54** in the presence of HBTU to afford tetraPEG (**13**).

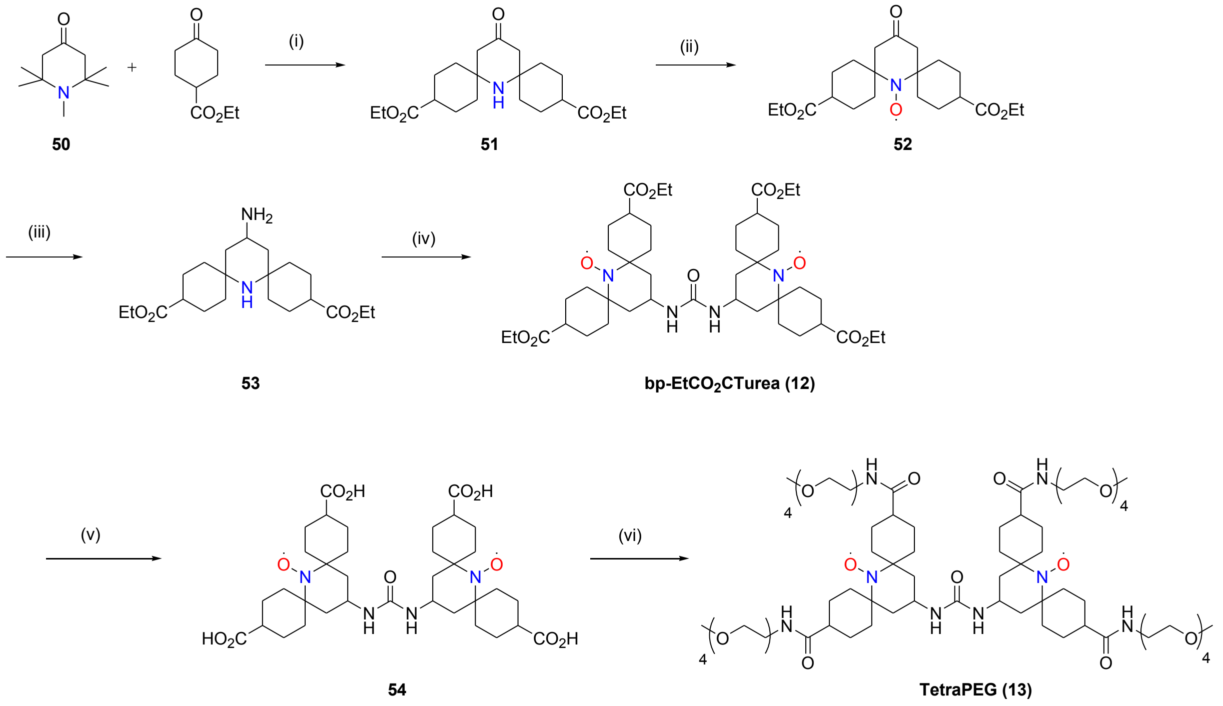

SI-Scheme 2: synthetic route to dinitroxides 12 and 13. Reagents and conditions: (i) NH_4Cl, DMSO, 60°C, 20h. (ii) H_2O_2 (30%), $Na_2WO_4.2H_2O$, EtOH, 25°C, 24h. (iii) $AcONH_4$, $NaBH_3CN$, MeOH, 25°C. (iv) Triphosgene, TEA, CH_2Cl_2, reflux, 6h. (v) NaOH, EtOH/THF, 80°C, 8h. (vi) DIPEA, HBTU, $H_2N(PEG)_4OMe$.

Synthetic route to KbCTurea$(PEG)_4$ (14)

Compound **55** was synthesized according to the literature procedure.[1] Amine **56** was prepared by reacting ketone **55** with tetra(ethylene glycol)methyl ether in the presence of sodium

triacetoxyborohydride. Ketone **55** was achieved by reductive amination using sodium cyanoborohydride and ammonium acetate yielded to amine **57**. Finally, a mixture of **56** and **57** was reacted with triphosgene to give KbCTurea(PEG)$_4$ (**14**).

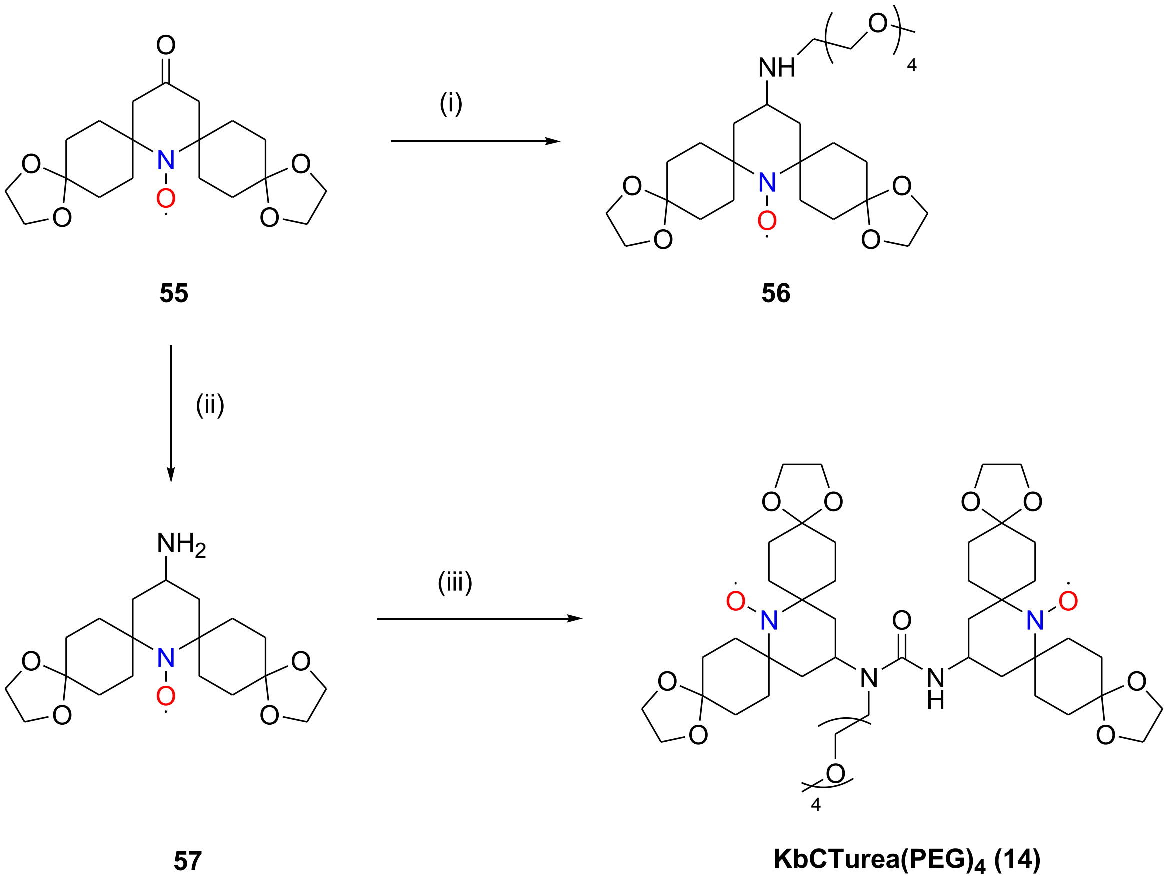

SI-Scheme 3: synthetic route to KbCTurea(PEG)$_4$ (14). Reagents and conditions: (i) $NH_2(CH_2CH_2)_4OCH_3$, $Na(OAc)_3BH$, AcOH, THF, 25°C, 16h. (ii) $AcONH_4$, $NaBH_3CN$, MeOH, 25°C, 20h. (iii) **56**, Triphosgene, TEA, CH_2Cl_2, 25°C, 12h.

Synthetic route to PyPolPEGNH$_2$ (17)

bPyTO (**43**) and N-acetyl-2,2'-ethylenedioxy bis-(ethylamine) were prepared according to the reported procedures.[1,2]

Dinitroxide **59** was synthesized using the same strategy than KbCTurea(PEG)$_4$ (**14**). First, amine **58** was prepared by reacting ketone **43** with N-acetyl-2,2'-ethylenedioxy bis-(ethylamine) in the presence of sodium triacetoxyborohydride. Then, a mixture of **58** and **49** was reacted in the presence of triphosgene to obtain dinitroxide **59**. Finally, acetamido group was removed in aqueous basic conditions to give PyPolPEGNH$_2$ (**17**).

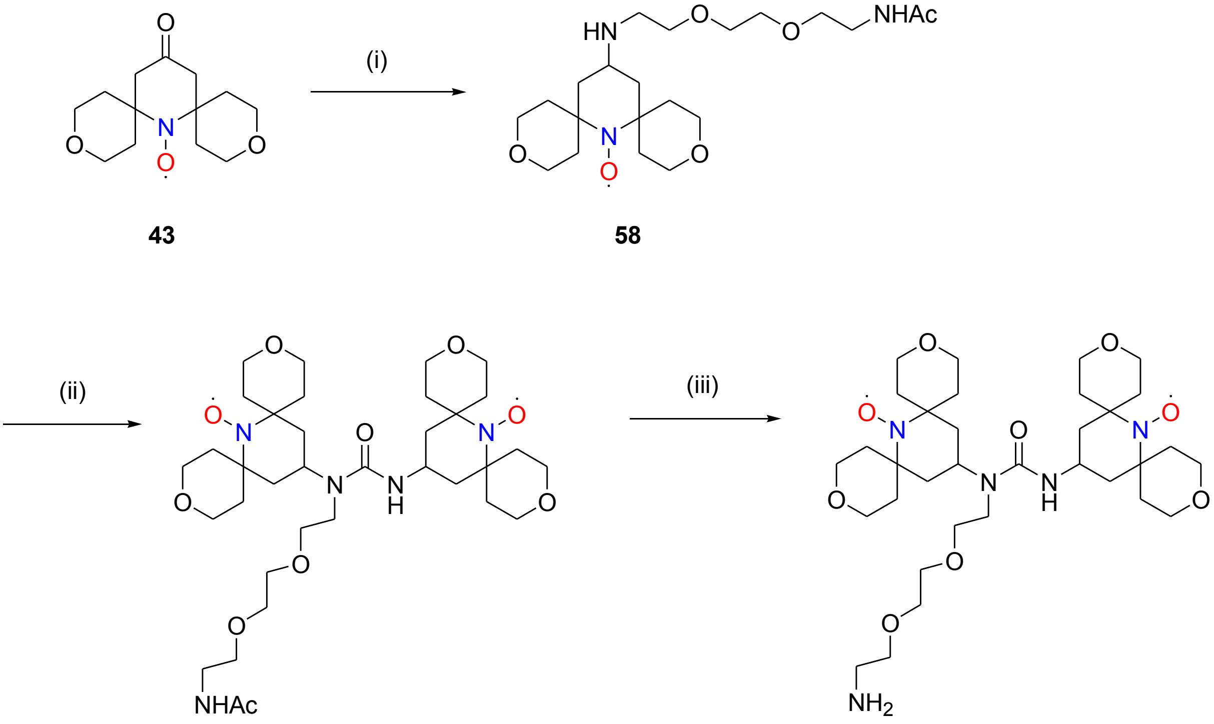

43 **58**

59 **PyPolPEGNH$_2$ (17)**

SI-Scheme 4: Synthetic route to **PyPolPEGNH$_2$ (17).** Reagents and conditions: (i) $NH_2(OCH_2CH_2)_2NHAc$, $Na(OAc)_3BH$, AcOH, THF, 25°C, 16h. (ii) **49**, triphosgene, TEA, CH_2Cl_2, 25°C, 12h. (iii) KOH, EtOH/Water, 50°C, 16h.

General synthetic route to b-3,5-diMePyTbK(38), TEKPol2 (34), TEKPol3 (35), TEKPol4 (36), TEKPol5 (37), (Adm)$_2$TbK (31), bEtTbk (29), bPEtTbK (30) and bTbK-d$_{24}$ (28)

According to the published procedure for TEKPol (**33**), a series of new dinitroxides **38**, **34**, **35**, **36**, **37**, **29**, **30**, **31** and **28** was prepared in a three or four steps sequence starting from 1,2,2,6,6-pentamethyl-4-piperidinone (**50**). First, the spiro(hetero)cyclohexyl moieties were introduced by Yamada's methods, using *cis*-2,6-dimethyltetrahydropyran-4-one, tetrahydro-4H-thiopyran-4-one, *cis*-3,5-diphenylcyclohexanone, *cis*-2,6-bis(phenyl)dihydro-*2H*-thiopyran-4(*3H*)-one, 4-(4-biphenylyl)cyclo-hexanone, 4-(4-methoxyphenyl)cyclohexanone, and 4-(4-oxo-cyclohexyl)benzoic acid ethyl ester as reactant, to give 2,6-dispisubstitued piperidin-4-one derivatives **60**, **61**, **62**, **63**, **70**, **71**, **72**, respectively and a mono spirosubstituted **76** piperidin-4-one was obtained with 2-adamantanone. Then, the spirosubstituted piperidin-4-one derivatives were reacted with pentaerythritol to afford bisketals **64**, **65**, **66**, **67**, **73**, **74**, **75** and **77**. The desulfurization of compounds **65** and **67** with Raney-Ni afforded compounds **68** and **69**. Finally, the new bisketal compounds were oxidized by hydrogen peroxide in the presence of sodium tungstate to yield the new dinitroxide biradicals **38**, **34**, **35**, **36**, **37**, **31**, **29** and **30**. bTbK-d$_{24}$ (**28**) was prepared in a two steps sequence using the same strategy described before starting from **78** which was synthesized following the general procedure of Lin *et al.*[3]

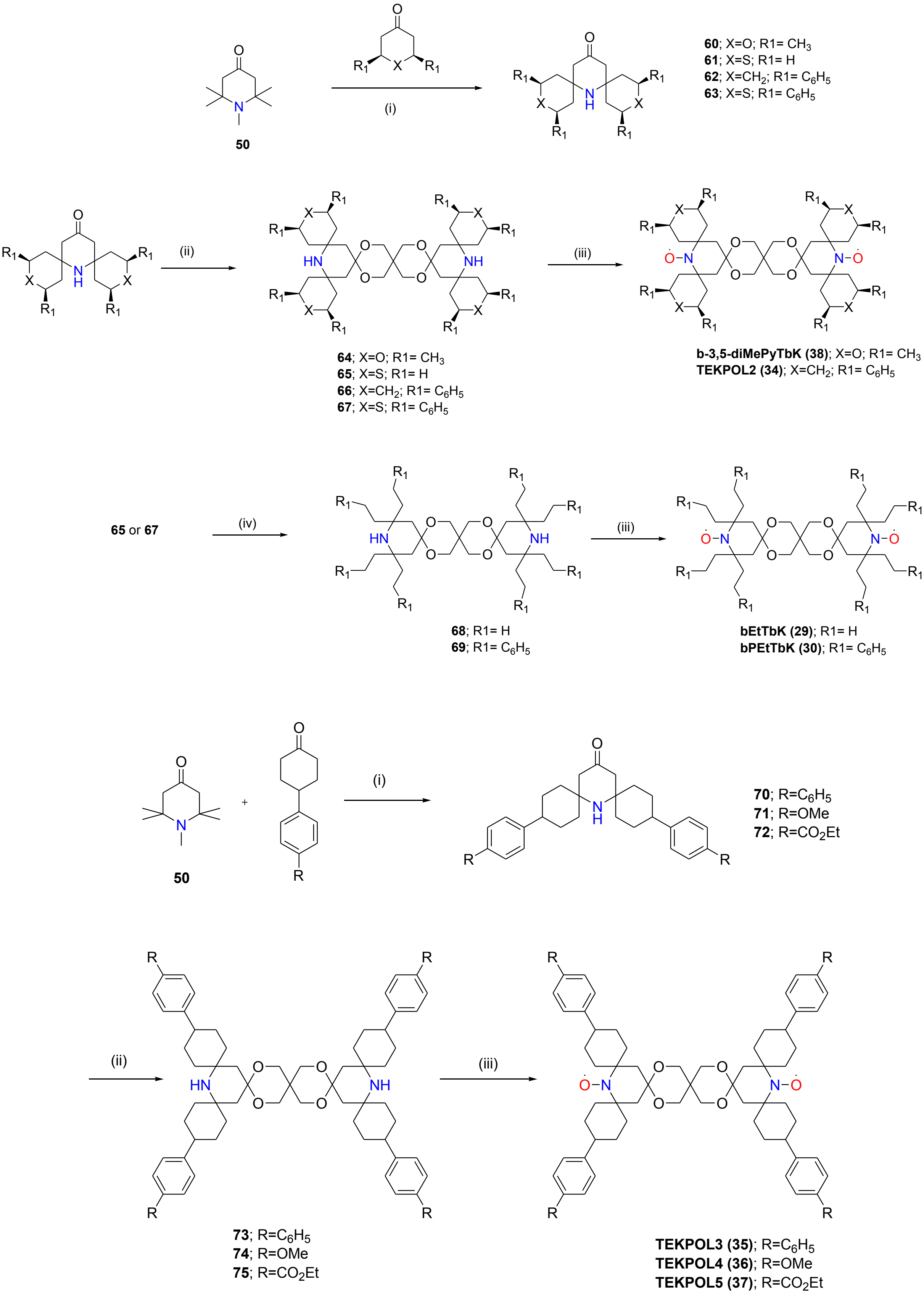

(i)
50
60; X=O; R1= CH3
61; X=S; R1= H
62; X=CH2; R1= C6H5
63; X=S; R1= C6H5
(ii)
(iii)
64; X=O; R1= CH3
65; X=S; R1= H
66; X=CH2; R1= C6H5
67; X=S; R1= C6H5
b-3,5-diMePyTbK (38); X=O; R1= CH3
TEKPOL2 (34); X=CH2; R1= C6H5
65 or 67
(iv)
(iii)
68; R1= H
69; R1= C6H5
bEtTbK (29); R1= H
bPEtTbK (30); R1= C6H5
50
(i)
70; R=C6H5
71; R=OMe
72; R=CO2Et
(ii)
(iii)
73; R=C6H5
74; R=OMe
75; R=CO2Et
TEKPOL3 (35); R=C6H5
TEKPOL4 (36); R=OMe
TEKPOL5 (37); R=CO2Et

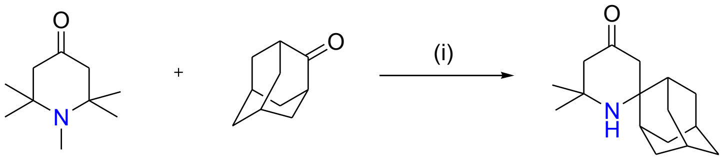

50 **76**

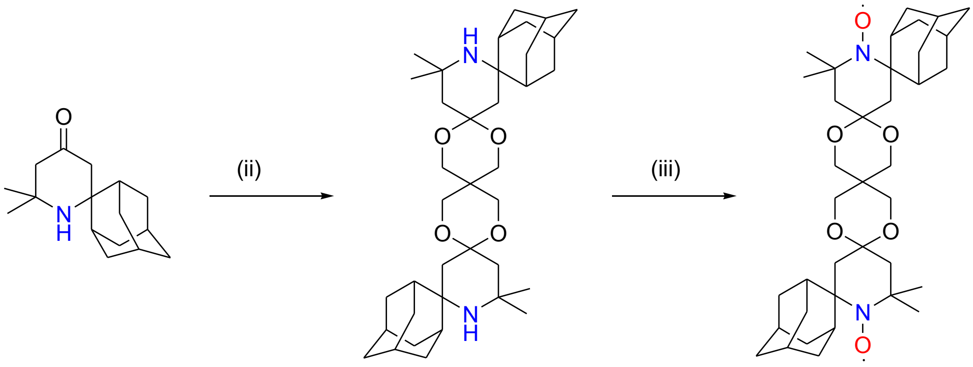

77 **(Adm)2TbK (31)**

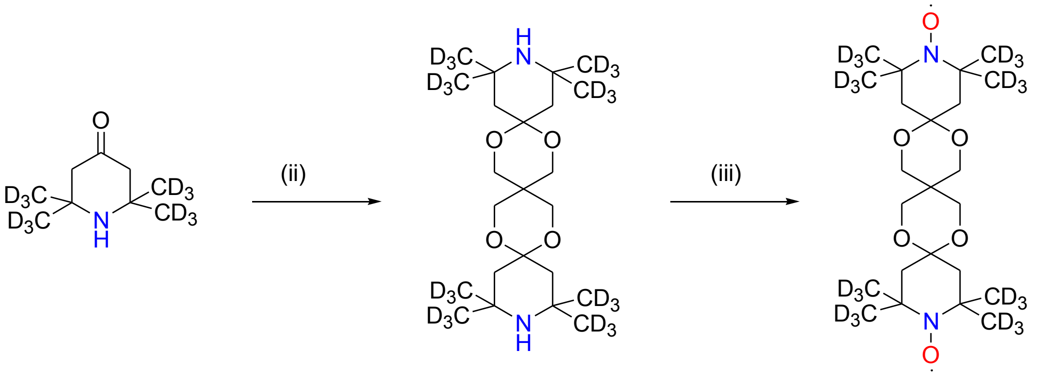

78 **79** **bTbk-d$_{24}$ (28)**

SI-Scheme 5: Reagents and conditions: (i) NH_4Cl, DMSO, 60°C, 20h. (ii) *p*-TsOH, pentaerythritol, PhMe, reflux, 24h. (iii) H_2O_2 (30%), $Na_2WO_4.2H_2O$, EtOH, 25°C, 24h. (iv) Ni-Raney, EtOH, reflux, 24h.

Synthetic route to b-3,5-diMeAMUPol (26)

b-3,5-diMeAMUPol (**26**) was synthesized using the similar strategy than KbCTurea(PEG)$_4$ (**14**) starting from **60**.

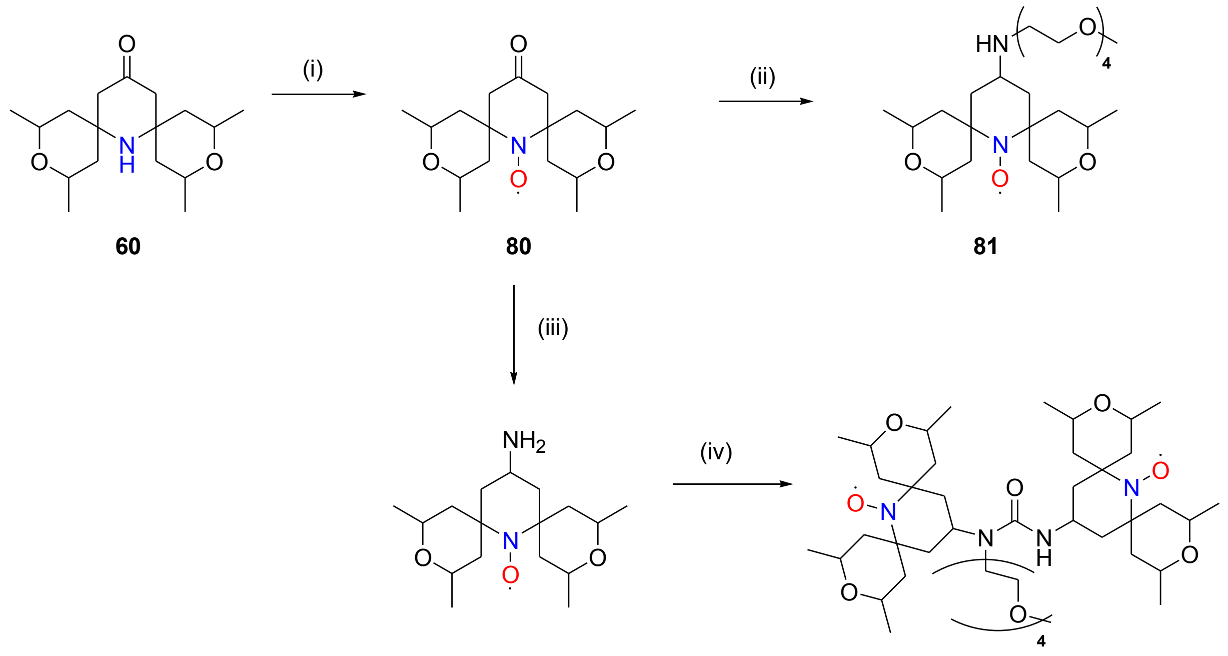

SI-Scheme 6: synthetic route to b-3,5-diMeAMUPol (**26**)**.**Reagents and conditions: (i) H_2O_2 (30%), $Na_2WO_4.2H_2O$, EtOH, 25°C, 24h. (ii) $NH_2(CH_2CH_2)_4OCH_3$, $Na(OAc)_3BH$, AcOH, THF, 25°C, 16h. (iii) $AcONH_4$, $NaBH_3CN$, MeOH, 25°C, 20h. (iv) 81, Triphosgene, TEA, CH_2Cl_2, 25°C, 12h.

Experimental Section

Compounds **43**, **49**, **50**, **55**, bTurea (**1**), bTurea(PEG)$_2$ (**2**), bTurea(PEG)$_4$ (**3**), bTurea(PEG)$_{8\text{-}12}$ (**4**), bTurea-C$_6$ (**7**), bCTurea(PEG)$_2$ (**8**), bCTurea(PEG)$_4$ (**9**), AMUPol (**15**), PyPolPEG$_2$OH (**16**), AMUPol(PEG)$_{8\text{-}12}$ (**18**), PyPol (**19**), PyPol-C$_6$ (**23**), PyPol-C$_6$OH(**24**), AMUPol-C$_6$ (**25**), bTbK (**27**), bCTbK(**32**), TEKPol (**33**) and TOTAPOL (**39**) were synthesized according to reported procedures.[1,4,5,6,7,8,9,10] Tetra(ethyleneglycol)methyl*, Cis*-2,6-dimethyltetrahydropyran-4-one, *cis*-3,5-diphenylcyclohexanone, *cis*-2,6-bis(phenyl)dihydro-*2H*-thiopyran-4(*3H*)-one, 4-(4-biphenylyl)cyclohexanone, 4-(4-methoxyphenyl)cyclohexanone, 4-(4-oxo-cyclohexyl)-benzoic acid ethyl ester were prepared according to the literature procedures.[11,12,13,14,15,16,17,18] All chemicals used in synthesis were purchased from Aldrich Chemical Co. Commercially available starting materials were used without further purification. Purification of products was accomplished by flash chromatography on silica gel (Merck silica gel 60, 230-400 Mesh). NMR measurements were recorded on a Bruker AVL 300 spectrometer (^{1}H-NMR 300.1 MHz and ^{13}C-NMR 75.5 MHz) using $CDCl_3$ as the solvent (internal reference). Splitting patterns are indicated as follows: br, broad; s, singlet; d, doublet; t, triplet; q, quadruplet; m, multiplet; dd, doublet of doublets. Mass spectra analyses were carried out using a Q-STAR elite at the Aix-Marseille Université Mass spectrum facility, spectropole Saint Jérôme Marseille. Melting point were determined using a Bibby SMP3 apparatus and were uncorrected. The final were purified to ≥ 95% and were confirmed by elemental analysis or HPLC. HPLC experiments were performed using Agilent 1200 system equipped with UV-Vis absorption detector. A fused core RP C18 column (Phenomenex, Kinetex C18, 100 mm x 4.6 mm, 2.6 µm) was used. Typically a gradient elution using aqueous mobile phase with increasing fraction of acetonitrile (from 10% to 40% over 5 min and from 40 to 100% over 5 min) in the presence of 0.1% TFA was used. The compounds were eluted using a flow rate of 1.5 mL/min.

General procedure for synthesis compound 41,42,44,45 and 48.

Under argon atmosphere a solution of ketone (1 mmol), methylamine hydrochloride or methylamine-D_3-hydrochloride (6 mmol) in dry MeOH was stirred at 25°C during 3h. After this time, $NaBH_3(CN)$ (0.8 mmol) was added and the reaction was stirred 24h at room temperature. Then a 10% K_2CO_3 aqueous solution was added. The mixture was concentrated under reduced pressure and the remaining aqueous phase was extracted twice with CH_2Cl_2, dried over Na_2SO_4 and concentrated under reduced pressure. The crude product was purified by SiO_2 column chromatography using CH_2Cl_2/EtOH as eluent in increasing polarity to yield the desired compounds.

Compound 41.
A total of 80 mg (43%) of **41** was obtained as a red solid. HRMS-ESI: calcd for $C_{10}H_{22}N_2O^{\cdot}$ ($[M+H]^+$) 186.1727 found 186.1727.

Compound 42.
A total of 100 mg (53%) of **42** was obtained as a red solid. HRMS-ESI: calcd for $C_{10}H_{19}D_3N_2O^{\cdot}$ ($[M+H]^+$) 189.1915 found 189.1914.

Compound 44.
A total of 180 mg (67%) of **44** was obtained as a red solid. HRMS-ESI: calcd for $C_{14}H_{26}N_2O_3^{\cdot}$ ($[M+H]^+$) 270.1938 found 270.1938.

Compound 45.
A total of 260 mg (85%) of **45** was obtained as a pale red solid. mp 131°C. ESI-MS m/z = 273 $[M+H]^+$; 295 $[M+Na]^+$.

Compound 48.
A total of 300 mg (71%) of **48** was obtained as a pale red solid. mp 185°C. ESI-MS m/z = 418 $[M+H]^+$; 440 $[M+Na]^+$.

General procedure for synthesis compounds 47, 53, 57 and 82.
Under argon atmosphere, a solution of ammonium acetate (10 mmol) in dry MeOH (3 mL) was stirred for 10 minutes. Then, a solution of ketone (1 mmol) in dry MeOH (5 mL) was added and the reaction was stirred at room temperature during 5h. Then, $NaBH_3(CN)$ (0.72 mmol) was added at 0°C and the reaction was stirred at 25°C during 20h. After this time, 20 mL of 10% K_2CO_3 aqueous solution was added. The mixture was concentrated under reduced pressure and the remaining aqueous phase was extracted with CH_2Cl_2 (2 x 60 mL). The organic layer was dried on Na_2SO_4, concentrated under reduced pressure and the residue was purified by SiO_2 column chromatography using CH_2Cl_2/EtOH as eluent as eluent in increasing polarity to yield the desired compounds.

Compound 47.
A total of 271 mg (66%) of **47** was obtained as a red solid. mp 218°C**.** ESI-MS *m/z* = 404 $[M+H]^+$; 426 $[M+Na]^+$.

Compound 53.
A total of 170 mg (43%) of **53** was obtained as a red solid. ESI-MS *m/z* = 396 $[M+H]^+$; 418 $[M+Na]^+$.

Compound 57.
A total of 150 mg (41%) of **57** was obtained as a red solid. ESI-MS *m/z* = 368 $[M+H]^+$.

Compound 82.
A total of 130 mg (42%) of **82** was obtained as a red solid. ESI-MS *m/z* = 312 $[M+H]^+$; $[M+Na]^+$.

General procedure for synthesis compounds 5, 6, 10, 11, 12, 20, 22.
To a solution of secondary amine or primary amine (0.5 mmol) and TEA (1.2 mmol) in CH_2Cl_2 was added at 0°C triphosgene (0.1 mmol) under argon atmosphere. The mixture was stirred under reflux during 6h. At the end of reaction, a 10% K_2CO_3 aqueous solution was added and the solution was extracted twice with CH_2Cl_2, dried over Na_2SO_4 and concentrated under reduced pressure. The crude

product was purified by SiO_2 column chromatography using CH_2Cl_2/EtOH as eluent in increasing polarity to yield the desired compounds.

bTurea-diMe (5)
A total of 23 mg (58%) of **5** was obtained as a red solid. HRMS-ESI: calcd for $C_{21}H_{41}N_4O_3^{2\cdot}$ ($[M+H]^+$) 397.3173 found 397.3173.

bTurea-diCD$_3$ (6)
A total of 21 mg (52%) of **6** was obtained as a red solid. HRMS-ESI: calcd for $C_{21}H_{34}D_6N_4O_3^{2\cdot}$ ($[M+H]^+$) 403.3550 found 403.3546.

PyPol-diMe (20)
A total of 20 mg (35%) of **20** was obtained as a red solid. HRMS-ESI: calcd for $C_{29}H_{49}N_4O_7^{2\cdot}$ ($[M+H]^+$) 565.3596 found 565.3595.

PyPol-diCD$_3$ (22)
A total of 27 mg (47%) of **22** was obtained as a red solid. HRMS-ESI: calcd for $C_{29}H_{43}D_6N_4O_7^{2\cdot}$ ($[M+H]^+$) 571.3972 found 571.3971.

TEKurea (10)
A total of 38 mg (46%) of **10** was obtained as a red solid. HRMS-ESI: calcd for $C_{55}H_{69}N_4O_3^{2\cdot}$ ($[M+H]^+$) 833.5364 found 833.53.

TEKureaDME (11)
A total of 43 mg (50%) of **11** was obtained as a red solid. HRMS-ESI: calcd for $C_{57}H_{73}N_4O_3^{2\cdot}$ ($[M+H]^+$) 861.5677 found 861.5671.

bp-EtCO$_2$CTurea (12)
A total of 58 mg (71%) of **12** was obtained as a red solid. HRMS-ESI: calcd for $C_{43}H_{72}N_5O_{11}^{2\cdot}$ ($[M+NH_4]^+$) 834.5223 found 834.5228.

Synthesis of compound 58.
To a solution of **43** (0.75 g, 2.94 mmol) and N-acetyl-2,2'-ethylenedioxy bis-(ethylamine) (0.6 g, 3.18 mmol) in dry THF was added AcOH (0.10 mL) to obtain pH around 4. After 5h at 25°C, $NaBH(OAc)_3$ was added and the reaction was stirred 16h at 25°C. After this time, the mixture was concentrated under reduced pressure. The residue was solubilized in CH_2Cl_2, washed with a saturated aqueous solution of $NaHCO_3$, dried over Na_2SO_4, concentrated under reduced pressure and the crude product was purified by SiO_2 column chromatography using CH_2Cl_2/EtOH (98/2) as eluent to give **58** (0.65 g, 51%) as a red oil. ESI-MS m/z = 429 $[M+H]^+$; 451 $[M+Na]^+$.

General procedure of compounds 14, 21, 26, 59.
A solution of secondary amine (1 mmol), primary amine (0.5 mmol), NEt_3 (1.2 mmol) and triphosgene (0.5 mmol) was stirred at room temperature in CH_2Cl_2 under argon atmosphere during 12h. At the end of reaction, the solution was washed with a saturated $NaHCO_3$ aqueous solution, dried over Na_2SO_4 and concentrated under reduced pressure. The crude product was purified by SiO_2 column chromatography using CH_2Cl_2/EtOH as eluent in increasing polarity to yield the desired compounds.

PyPol-CD$_3$ (21)

A total of 162 mg (58%) of **21** was obtained as a red solid. HRMS-ESI: calcd for $C_{28}H_{43}D_3N_4O_7^{2\cdot}$ ($[M+H]^+$) 554.3628 found 554.3623.

KbCTurea(PEG)$_4$ (14)
A total of 130 mg (26%) of **14** was obtained as a red solid. ESI-MS m/z = 951 $[M+H]^+$; 968 $[M+NH_4]^+$; 973 $[M+Na]^+$. HRMS-ESI: calcd for $C_{48}H_{78}N_4O_{15}^{2\cdot}$ ($[M+H]^+$) 951.5536 found 951.5532. HPLC: rt= 11.07 min.

b-3,5-diMeAMUPol (26)

A total of 140 mg (33%) of **26** was obtained as a red solid. HRMS-ESI: calcd for $C_{44}H_{78}N_4O_{11}^{2\cdot}$ ($[M+H]^+$) 839.5740 found 839.5740.HPLC: rt= 11.13 min.

PyPolPEGNHAc (59)

A total of 165 mg (47%) of **59** was obtained as a purple oil. ESI-MS *m/z* = 710 $[M+H]^+$; 732 $[M+Na]^+$.

Synthesis of PyPolPEGNH₂ (17).

To a stirred solution of **59** (165 mg, 0.23 mmol) in ethanol was added a solution of KOH (1.27 g, 19.2 mmol) in water (0.8 mL) at 25°C. The mixture was stirred at 50°C for 16h. After this time, the mixture was concentrated under reduced pressure, diluted in CH_2Cl_2, washed with brine (20 mL), dried over Na_2SO_4 and concentrated under reduced pressure. The crude product was purified by SiO_2 column chromatography using CH_2Cl_2/EtOH/NH_4OH (9/1/1) to give **17** (0.1 g, 65%) as a purple solid. ESI-MS *m/z* = 668 $[M+H]^+$; 690 $[M+Na]^+$. HRMS-ESI: calcd for $C_{33}H_{58}N_5O_9^{2\cdot}$ ($[M+H]^+$) 668.4229 found 668.4225.

Synthesis of compound 51.

To a stirred mixture of 1,2,2,6,6-pentamethypiperidin-4-one **50** (1.69 g, 10.00 mmol) and ethyl 4-cyclohexanonecarboxylate (5.0 g, 29.40 mmol) in dimethylsulfoxide (50 mL), NH_4Cl (3.3 g, 61.68 mmol) was added at room temperature. The mixture was heated at 60°C during 20 h, then it was diluted with water (200 mL), pH was adjusted at 2 with 1N HCl aqueous solution (40 mL) and the mixture was extracted with diethylether (2 x 100 mL). The aqueous layer was adjusted to pH 11 by adding 50 mL of saturated K_2CO_3 aqueous solution and then extracted with chloroform (2 x 100 mL). The organic phase was concentrated under reduced pressure, washed with brine (100 mL), dried over Na_2SO_4, and the solvent was distilled under reduced pressure. The crude product was purified by SiO_2 column chromatography using pentane / ethylacetate (1/1) to afford **51** (0.56 g, 15%) as a white solid. ^1H NMR (300 MHz, $CDCl_3$) δ 1.15-1.19 (t, 6H), 1.65-1.85 (m, 16H), 2.15-2.25 (m, 2H), 2.33 (s, 4H), 4.02-4.07 (q, 4H). ^{13}C NMR (300 MHz, $CDCl_3$) δ 14.05, 24.63, 39.19, 41.72, 49.65, 56.37, 60.13, 175.06, 209.98. ESI-MS *m/z* = 380 $[M+H]^+$; 402 $[M+Na]^+$.

Synthesis of compound 52.

Compound **51** (0.5 g, 1.32 mmol) and $Na_2WO_4.2H_2O$ (52 mg, 0.16 mmol) were stirred in ethanol (15 mL) and H_2O_2 (30%, 5.46 mmol, 625 µL) was slowly added at 0°C. The mixture was stirred for 24 h at room temperature, then K_2CO_3 (0.80 g) was added and the solution was extracted twice with chloroform (50 mL). The organic layer was dried over Na_2SO_4 and distilled under reduced pressure. The crude product was purified by SiO_2 column chromatography using Chloroform / MeOH (99/1) as eluent to provide compound **52** (0.43 g, 50%) as a pale red solid. ESI-MS *m/z* = 395 $[M+H]^+$; 412 $[M+NH_4]^+$; 417 $[M+Na]^+$.

Synthesis of compound 80.

Compound **60** (0.8 g, 2.71 mmol) was oxidized according to the general procedure described above for the oxidation of **51**. The crude product was purified by SiO_2 column chromatography using CH_2Cl_2 / EtOH (99/1) as eluent to provide compound **80** (0.75 g, 89%) as a yellow solid. mp 165-170°C. X-band EPR spectrum (293 K, in CH_2Cl_2): triplet, A_N = 1.47 mT. HRMS-ESI: calcd for $C_{17}H_{28}NO_4^{\cdot}$ ($[M+H]^+$) 311.2091 found 311.2089.

Synthesis of compound 54.

A solution of **12** (0.25 g, 0.3 mmol) and NaOH (0.24 g, 6.1 mmol) in a mixture EtOH/THF (1mL/1mL) was stirred at 80°C for 8h. It was then allowed to cool to room temperature. At 0°C, a 6 M HCl solution was added until pH 3 and the product precipitated. It was then filtered off, washed with EtOH and H_2O and dried to give **54** (0.15 g, 71%). HRMS-ESI: calcd for $C_{35}H_{52}N_4O_{11}$ ($[M+Na]^+$) 727.3525 found 727.3525.

Synthesis of TetraPEG (13).

To a solution of **54** (50 mg, 0.07 mmol), DIPEA (0.15 mL, 0.84 mmol) and HBTU (0.32 mg, 0.84 mmol) in dry DMF (2 mL) was added aminePEG (118 mg, 0.57 mmol) and the solution was stirred at room temperature for 16h. Brine was added and the solution was extracted with CH_2Cl_2, dried over Na_2SO_4 and the solvent was evaporated under reduced pressure. Then, the residue was dried under high vacuum to remove the remaining DMF. It was then purified by SiO_2 column chromatography using CH_2Cl_2/EtOH (9/1) as eluent to give TetraPEG (**13**) (70 mg, 69%) as a red oil. HRMS-ESI: calcd for $C_{71}H_{136}N_{10}O_{23}$ ($[M+2NH_4]^{2+}$) 748.4884 found 748.4888.

General procedure for compounds 56 and 81.
To a solution of ketone (1 mmol), tetra(ethylene glycol) methy ether amine (1.3 mmol) in dry THF was added AcOH to adjust pH at around 6-7 and the solution was stirred at room temperature for 2h. Then, $Na(OAc)_3BH$ (1.5 mmol) was added and the reaction was stirred at 25°C for 16h. After this time, the mixture was concentrated under reduced pressure. The residue was solubilized in CH_2Cl_2, washed with a saturated aqueous solution of $NaHCO_3$, dried over Na_2SO_4, concentrated under reduced pressure and the crude product was purified by SiO_2 column chromatography using CH_2Cl_2/EtOH as eluent in increasing polarity to yield the desired compounds.

Compound 56.
A total of 280 mg (50%) of **56** was obtained as a red solid. ESI-MS *m/z* = 558 $[M+H]^+$; 564 $[M+Li]^+$; 580 $[M+Na]^+$.

Compound 81.
A total of 290 mg (57%) of **81** was obtained as a red solid. ESI-MS *m/z* = 502 $[M+H]^+$; 524 $[M+Na]^+$.

Synthesis of compound 60.
Compound **60** was prepared according to the general procedure described for **51**, using *cis*-2,6-dimethyltetrahydropyran-4-one (3.84 g, 30.00 mmol) in place of ethyl 4-cyclohexanonecarboxylate. The crude product was purified by SiO_2 column chromatography using pentane / ethylacetate (90/10) to afford **60** (0.36 g, 12 %) as a white solid. The structure of compound **60** was determined by single crystal X-ray diffraction. mp 126-128°C; ^{1}H NMR (300 MHz, $CDCl_3$) δ 1.11 (s, 6H), 1.13 (s, 6H), 1.15-1.26 (m, 5H), 1.59-1.66 (m, 4H), 2.46 (s, 4H), 3.44-3.52 (m, 4H). ^{13}C NMR (300 MHz, $CDCl_3$) δ 21.84, 48.09, 49.55, 55.82, 69.05, 209.66. ESI-MS *m/z* = 296 $[M+H]^+$. HRMS-ESI: calcd for $C_{17}H_{29}NO_3$ ($[M+H]^+$) 296.2220 found 296.2219.

Synthesis of compound 61.
Compound **61** was prepared according to the general procedure described for **51**, using tetrahydro-4*H*-thiopyran-4-one (3.48 g, 30.00 mmol) in place of ethyl 4-cyclohexanonecarboxylate. The crude product was purified by SiO_2 column chromatography with pentane / ethylacetate (90/10) to afford **61** (0.89 g, 33 %) as a white solid. mp 156°C (lit.,[1] 155-157°C). ^{1}H NMR (300 MHz, $CDCl_3$) δ 0.8 (brs, 1H), 1.75-1.91 (m, 8H), 2.28 (s, 4H), 2.44-2.50 (m, 4H), 2.88-2.96 (m, 4H). ^{13}C NMR (300 MHz, $CDCl_3$) δ 24.03, 41.40, 53.00, 55.71, 208.99. ESI-MS *m/z* = 272 $[M+H]^+$.

Synthesis of compound 62.
Compound **62** was prepared according to the general procedure described for **51**, using *cis*-3,5-diphenylcyclohexanone (8.90 g, 35.60 mmol) in place of ethyl 4-cyclohexanonecarboxylate. The crude product was purified by SiO_2 column chromatography using pentane / ethylacetate (90/10) to afford **62** (0.51 g, 8 %) as a white solid (as a mixture of diasteroisomers). ^{1}H NMR (300 MHz, $CDCl_3$) δ 1.48-1.70 (m, 4H), 1.90-2.18 (m, 8H), 2.37-2.65 (m, 4H), 2.82-3.49 (m, 4H), 7.10-7.40 (m, 20H). ESI-MS *m/z* = 540 $[M+H]^+$; 546 $[M+Li]^+$.

Synthesis of compound 63.
Compound **63** was prepared according to the general procedure described for **51**, using *cis*-2,6-bis(phenyl)dihydro-2*H*-thiopyran-4(3*H*)-one (8.04g, 30.00 mmol) in place of ethyl 4-cyclohexanonecarboxylate. The crude product was purified by SiO_2 column chromatography with

CH_2Cl_2/EtOH (95/5) to afford **63** (0.43 g, 8 %) as a white solid. (as a mixture of diasteroisomers). ^1H NMR (300 MHz, $CDCl_3$) δ 1.92-2.68 (m, 12H), 4.13-4.80 (m, 4H), 7.20-7.45 (m, 20H). ESI-MS *m/z* = 576 $[M+H]^+$; 598 $[M+Na]^+$.

Synthesis of compound 70.
Compound **70** was prepared according to the general procedure described for **51**, using 4-(4-biphenylyl)cyclohexanone (7.5 g, 30.00 mmol) in place of ethyl 4-cyclohexanonecarboxylate. The crude product was purified by SiO_2 column chromatography with pentane / ethylacetate (70/30) to afford **70** (0.16 g, 3%) as a white solid. ^1H NMR (300 MHz, $CDCl_3$) δ 1.60-1.70 (m, 8H), 1.84-2.00 (m, 9H), 2.50-2.59 (m, 6H), 7.28-7.62 (m, 18H). ^{13}C NMR (300 MHz, $CDCl_3$) δ 29.27, 40.22, 42.17, 48.07, 55.77, 125.99, 126.33, 127.69, 138.13, 139.99, 144.45, 209.81. ESI-MS *m/z* = 540 $[M+H]^+$; 546 $[M+Li]^+$.

Synthesis of compound 71.
Compound **71** was prepared according to the general procedure described for **51**, using 4-(4-methoxyphenyl)cyclohexanone (6.20 g, 30.00 mmol) in place of ethyl 4-cyclohexanonecarboxylate. The crude product was purified by SiO_2 column chromatography using pentane / ethylacetate (70/30) to afford **71** (180 mg, 4 %) as a white solid. ^1H NMR (300 MHz, $CDCl_3$) δ 1.44-2.00 (m, 17H), 2.40-2.55 (m, 6H), 3.78 (m, 6H), 6.83 (d, J = 8.34 Hz, 4H), 7.12 (d, J = 8.44 Hz, 4H). ^{13}C NMR (300 MHz, $CDCl_3$) δ 30.50, 41.25, 42.62, 49.09, 55.26, 56.77, 113.81, 127.58, 138.56, 157.93, 211.02. ESI-MS *m/z* = 448 $[M+H]^+$; 554 $[M+Li]^+$.

Synthesis of compound 72.
Compound **72** was prepared according to the general procedure described for **51**, using 4-(4-oxo-cyclohexyl)-benzoic acid ethyl ester (7.38 g, 30.00 mmol) in place of ethyl 4-cyclohexanonecarboxylate. The crude product was purified by SiO_2 column chromatography with DCM/EtOH 99/1 to afford **72** (0.55 g, 10 %) as a white solid. mp 193°C. ^1H NMR (300 MHz, $CDCl_3$) δ = 1.36 (t, J = 7.10 Hz, 6H), 1.54-1.66 (m, 8H), 1.75-2.01 (m, 8H), 2.53 (s, 4H), 4.33 (d, J = 7.10 Hz, 4H), 7.22 (d, J = 8.25 Hz, 2H), 7.93 (d, J = 8.25 Hz, 4H). ^{13}C NMR (75 MHz, $CDCl_3$) δ = 14.32, 29.95, 40.97, 43.53, 49.00, 56.72, 60.76, 126.70, 128.48, 129.72, 151.51, 166.53, 210.55. ESI-MS m/z = 532 $[M+H]^+$; 554 $[M+Na]^+$.

Synthesis of compound 76.
Compound **76** was prepared according to the general procedure described for **51**, using 2-adamantanone (4.5 g, 30.00 mmol) in place of ethyl 4-cyclohexanonecarboxylate. The crude product was purified by SiO_2 column chromatography using pentane / ethylacetate (95/5) to afford **76** (0.13 g, 5%). ^1H NMR (300 MHz, $CDCl_3$) δ 1.17 (s, 6H), 1.40-1.90 (m, 15H), 2.20 (s, 2H), 2.47 (s, 2H). ^{13}C NMR ($CDCl_3$) δ 26.97, 27.13, 31.95, 32.90, 33.43, 38.40, 38.46, 49.94, 54.33, 54.73, 61.51, 211.68. ESI-MS *m/z* = 248 $[M+H]^+$; 270 $[M+Na]^+$.

General procedure of synthesis of compound 64, 65, 66, 73, 74, 75, 77 and 79.
Ketone (1 mmol) was dissolved in toluene (60 mL), pentaerythritol (0.40 mmol) and *p*-toluenesulfonic acid (0.8 mmol) were added to the stirred solution. The mixture was heated at reflux in a Dean Stark system for 24 h. After cooling, the solution was concentrated under reduced pressure and 10% Na_2CO_3 aqueous solution was added. The mixture was extracted twice with chloroform (40 mL), dried over Na_2SO_4 and the solvent was distilled under reduced pressure. The crude product was purified by SiO_2 column chromatography using CH_2Cl_2/EtOH as eluent in increasing polarity to yield the desired compounds

Compound 64.
A total of 85 mg (31%) of **64** was obtained as a white solid .^{13}C NMR ($CDCl_3$) δ 22.16, 32.83, 38.19, 48.43, 51.75, 63.39, 69.53, 99.18. HRMS-ESI: calcd for $C_{39}H_{66}N_2O_8$ ($[M+H]^+$) 691.4892 found 691.4888.

Compound 65.

A total of 182 mg (70 %) of **65** was obtained as a white solid. ^{1}H NMR ($CDCl_3$) δ 1.67 (s, 8H), 1.70-1.88 (m, 16H), 2.39-2.47 (m, 8H), 2.88-2.99 (m, 8H), 3.72 (s, 8H) .^{13}C NMR ($CDCl_3$) δ 24.08, 32.72, 41.41, 42.71, 51.45, 63.37, 99.05. ESI-MS *m/z* = 643 $[M+H]^+$.

Compound 66.
A total of 345 mg (73 %) of **66** was obtained as a white solid (as a mixture of diasteroisomeric compounds). ^{1}H NMR ($CDCl_3$) δ 1.50-2.20 (m, 32H), 2.70-3.28 (m, 8H), 3.50-3.92 (m, 8H), 7.00-7.56(m, 40H). ESI-MS *m/z* =1179 $[M+H]^+$; 590 $[M+2H]^{2+}$.

Compound 73.
A total of 200 mg (42 %) of **73** was obtained as a white solid. . ^{1}H NMR ($CDCl_3$) δ 1.50-1.73 (m, 18H), 1.80-2.13 (m, 25H), 2.57 (m, 4H), 3.83 (s, 8H), 7.25-7.60 (m, 40H) .^{13}C NMR ($CDCl_3$) δ 30.63, 33.19, 37.84, 41.12, 43.19, 52.46, 63.72, 99.78, 126.97, 127.04, 127.13, 128.67, 138.94, 141.03, 145.90. ESI-MS *m/z* =1179 $[M+H]^+$; 590 $[M+2H]^{2+}$.

Compound 74.
A total of 190 mg (47 %) of **74** was obtained as a white solid. ^{1}H NMR ($CDCl_3$) δ 1.20-2.00 (m, 40H), 2.34-2.48 (m, 4H), 3.70 (s, 20H), 6.77 (d, J= 8.16 Hz, 8H), 7.07 (d, J= 8.34 Hz, 8H).^{13}C NMR ($CDCl_3$) δ 30.81, 33.16, 37.70, 40.93, 42.59, 52.72, 55.22, 63.67, 99.67, 113.70, 127.53, 138.91, 157.80. ESI-MS *m/z* =995 $[M+H]^+$.

Compound 75.
A total of 390 mg (84 %) of **75** was obtained as a white solid.. ^{1}H NMR (300 MHz, $CDCl_3$) δ 1.32 (t, J = 7.1 Hz, 12H), 1.35-1.65 (m, 16H), 1.70-1.85 (m, 8H), 1.89 (s, 8H), 1.95-2.05 (m, 8H), 2.52 (m, 4H), 3.77 (s, 8H), 4.29 (q, J = 7.1 Hz, 8H), 7.22 (d, J = 8.2 Hz, 8H), 7.90 (d, J = 8.2 Hz, 8H). ^{13}C NMR (75 MHz, $CDCl_3$) δ 14.42, 30.46, 33.16, 37.89, 41.03, 43.76, 52.44, 60.81, 63.78, 99.75, 126.80, 128.39, 129.72, 152.10, 166.62. ESI-MS m/z = 1163 $[M+H]^+$.

Compound 77.
A total of 173 mg (73 %) of **77** was obtained as a white solid. ^{13}C NMR ($CDCl_3$) δ 27.35, 31.97, 32.27, 32.69, 34.08, 34.12, 37.97, 38.35, 38.91, 40.85, 41.06, 50.62, 56.52, 63.27, 63.55, 100.03. ESI-MS *m/z* = 595 $[M+H]^+$; 298 $[M+2H]^{2+}$.

Compound 79.
A total of 150 mg (86%) of **79** was obtained as a white solid. ^{1}H NMR ($CDCl_3$) δ 1.59 (s, 8H), 3.68 (s, 8H). ^{13}C NMR ($CDCl_3$) δ 30.82 (m), 32.29, 42.64, 50.37, 63.18, 99.28. ESI-MS *m/z* = 435 $[M+H]^+$; 452 $[M+NH_4]^+$; 457 $[M+Na]^+$; 473 $[M+K]^+$.

Synthesis of compound 68.
A suspension of Raney Ni (50% in water) was added to a solution of **65** (0.20 g, 0.31 mmol) in ethanol (25 mL). The mixture was stirred at reflux during 24 h. After cooling, the solution was filtered through a pad of Celite and the filtrate was concentrated under reduced pressure. The residue was dissolved in chloroform (30 mL), washed with 10% K_2CO_3 aqueous solution (30 mL), dried over Na_2SO_4 and the solvent was distilled under reduced pressure to yield **68** (30 mg, 19 %) as a white solid. ^{1}H NMR ($CDCl_3$) δ 0.70-0.76 (m, 24H), 1.26-1.34 (m, 8H), 1.43-1.51 (m, 8H), 1.58 (s, 8H), 3.66 (s, 8H) .^{13}C NMR ($CDCl_3$) δ 8.04, 32.17, 32.74, 39.49, 54.83, 63.49, 99.79. ESI-MS *m/z* =523 $[M+H]^+$.

Synthesis of compound 69.
Compound **63** (0.43 g, 0.74 mmol) was dissolved in toluene (80 mL), pentaerythritol (45 mg, 0.33 mmol) and *p*-toluenesulfonic acid (0.13 g, 0.65 mmol) were added to the stirred solution. The mixture was refluxed in a Dean Stark system for 24 h. After cooling, the solution was concentrated under reduced pressure and 10% Na_2CO_3 aqueous solution (100 mL) was added. The mixture was extracted twice with chloroform (150 mL), dried over Na_2SO_4 and the solvent was distilled under reduced pressure. The residue was purified by SiO_2 column chromatography with CH_2Cl_2/EtOH as eluent (90/10) to afford **67** (0.16 g, 38 %) as a white solid. ESI-MS *m/z* = 1251 $[M+H]^+$; 626 $[M+2H]^{2+}$.

Then, a suspension of Raney/Ni (50% in water) was added to a solution of **67** (0.16 g, 0.12 mmol) in ethanol (60 mL). The mixture was stirred at reflux during 24 h. After cooling, the solution was filtered through a pad of Celite and the filtrate was concentrated under reduced pressure. The residue was dissolved in chloroform (30 mL), washed with 10% K_2CO_3 aqueous solution (30 mL), dried over Na_2SO_4 and the solvent was distilled under reduced pressure. The residue was purified by AlO_2 column chromatography with CH_2Cl_2/EtOH (99/1) to give **69** (50 mg, 37 %) recrystallized from DCM/pentane as a white solid. ^{1}H NMR ($CDCl_3$) δ 1.80-1.99 (m, 24H), 2.62-2.72 (m, 16H), 3.76 (s, 8H), 7.10-7.30 (m, 40H) .^{13}C NMR ($CDCl_3$) δ 30.45, 33.09, 40.00, 43.12, 55.09, 63.61, 99.48, 125.67, 128.36, 128.39, 142.73. ESI-MS *m/z* =1132 $[M+H]^+$. 566 $[M+2H]^{2+}$.

Synthesis of compound 78.
Compound **78** was prepared using a modified procedure reported by Lin *et al.*[17]. 2,2,6,6-Tetraperdeuteromethyl-3,3,5,5-tetradeuteropiperidine was stirred in pure water (H_2O) and freeze dried (repeated 2 times) to obtain **78**. ^{1}H NMR ($CDCl_3$) δ 2.16 (s). ^{13}C NMR (300 MHz, $CDCl_3$) δ 31 (m), 53.82, 54.79, 210.81. ESI-MS *m/z* = 168 $[M+H]^+$; 190 $[M+Na]^+$.

Synthesis of b-3,5-diMePyTbK (38).
Compound **64** (35 mg, 0.05 mmol) and $Na_2WO_4.2H_2O$ (2 mg, 0.006 mmol) were stirred in ethanol (5 mL) and H_2O_2 (30%, 0.21 mmol, 24 µL) was slowly added at 5°C. The mixture was stirred for 24 h at room temperature, then K_2CO_3 (0.10 g) was added and the solution was extracted twice with chloroform (30 mL). The organic layer was dried over Na_2SO_4 and distilled under reduced pressure. The crude product was purified by SiO_2 column chromatography using Chloroform / MeOH (99/1) as eluent to provide pure b-3,5-diMePyTbK **(38)** (18 mg, 50%) as a pale red solid. X-band EPR spectrum (293 K, in CH_2Cl_2): triplet, A_N = 1.53 mT. ESI-MS *m/z* = 721 $[M+H]^+$. HRMS-ESI: calcd for $C_{39}H_{64}N_2O_{10}^{\cdot\cdot}$ ($[M+H]^+$) 721.4634 found 721.4637. HPLC: rt=10.28 min.

Synthesis of TEKPOL2 (34).
The diamine **66** (85 mg, 0.07 mmol) was oxidized according to the general procedure described above for the oxidation of **64**. The crude product was purified by SiO_2 column chromatography using chloroform as eluent to provide pure TEKPol2 (**34**) (39 mg, 45%), which was recrystallized from acetone to yield pale red small crystals. X-band EPR spectrum (293 K, in CH_2Cl_2): triplet, A_N = 1.48 mT. mp 230-233°C.ESI-MS *m/z* = 1209 $[M+H]^+$; 1231 $[M+Na]^+$. HRMS-ESI calcd for $C_{83}H_{88}N_2O_6^{\cdot\cdot}$ ($[M+NH_4]^+$) 1226.6981 found 1226.6956.Elemental analysis: C, 82.04; H, 7.34; N, 2.32 calcd for $C_{83}H_{88}N_2O_6^{2\cdot}$: C, 82.41; H, 7.33; N, 2.32.

Synthesis of bEtTbK (29).
The diamine **68** (30 mg, 0.06 mmol) was oxidized according to the general procedure described above for the oxidation of **64**. The crude product was purified by SiO_2 column chromatography using CH_2Cl_2/EtOH (99/1) as eluent to provide pure bEtTbK (**29**) as a pale yellow solid (19 mg, 57%). X-band EPR spectrum (293 K, in CH_2Cl_2): triplet, A_N = 1.445 mT. mp 140-142°C. ESI-MS *m/z* = 553 $[M+H]^+$; 575 $[M+Na]^+$; 591 $[M+K]^+$. HRMS-ESI: calcd for $C_{31}H_{56}N_2O_6^{\cdot\cdot}$ ($[M+H]^+$) 553.4211 found 553.4211. HPLC: rt = 12.731 min.

Synthesis of bPEtTbK (30).
The diamine **69** (50 mg, 0.044 mmol) was oxidized according to the general procedure described above for the oxidation of **64**. The crude product was purified by SiO_2 column chromatography using CH_2Cl_2 as eluent to provide bPEtTbK (**30**) as a red solid (30 mg, 60%). X-band EPR spectrum (293 K, in CH_2Cl_2): triplet, A_N = 1.42 mT. ESI-MS *m/z* = 1161 $[M+H]^+$; 1199 $[M+K]^+$. HRMS-ESI: calcd for $C_{79}H_{88}N_2O_6^{\cdot\cdot}$ ($[M+NH_4]^+$) 1178.6981 found 1178.6980.

Synthesis of TEKPOL3 (35).
The diamine **73** (25 mg, 0.02 mmol) was oxidized according to the general procedure described above for the oxidation of **64**. The crude product was purified by SiO_2 column chromatography using chloroform as eluent to provide pure TEKPol3 **(35)** (13 mg, 54%) as a pale red solid. X-band EPR

spectrum (293 K, in CH_2Cl_2): triplet, A_N = 1.51 mT. mp 275-278°C.ESI-MS *m/z* = 1209 $[M+H]^+$; 1226 $[M+NH_4]^+$. HRMS-ESI calcd for $C_{83}H_{88}N_2O_6^{\cdot\cdot}$ ($[M+H]^+$) 1209.6715 found 1209.6720.Elemental analysis: C, 81.91; H, 7.57; N, 2.19 calcd for $C_{83}H_{88}N_2O_6^{\cdot\cdot}$: C, 82.41; H, 7.33; N, 2.32.

Synthesis of TEKPOL4 (36).
The diamine **74** (40 mg, 0.04 mmol) was oxidized according to the general procedure described above for the oxidation of **64**. The crude product was purified by SiO_2 column chromatography using chloroform as eluent to provide pure TEKPol4 (**36**) as a red solid (19 mg, 46%). X-band EPR spectrum (293 K, in CH_2Cl_2): triplet, A_N = 1.506 mT. mp 245-248°C.ESI-MS *m/z* = 1025 $[M+H]^+$; 1047 $[M+Na]^+$. HRMS-ESI calcd for $C_{63}H_{80}N_2O_{10}^{\cdot\cdot}$ ($[M+H]^+$) 1025.5886 found 1025.5901.Elemental analysis: C, 73.96; H, 8.05; N, 2.69 calcd for $C_{63}H_{80}N_2O_{10}^{2\cdot}$: C, 73.80; H, 7.86; N, 2.73.

Synthesis of TEKPOL5 (37).
The diamine **75** (0.2 g, 0.17 mmol) was oxidized according to the general procedure described above for the oxidation of **64**. The crude product was purified by SiO_2 column chromatography using CH_2Cl_2/EtOH (99/1) as eluent to provide pure TEKPol5 (**37**) as a red solid (0.16 mg, 80%). X-band EPR spectrum (293 K, in CH_2Cl_2): triplet, A_N = 1.505 mT. HRMS-ESI: calcd for $C_{71}H_{90}N_2O_{14}^{2\cdot}$ ($[M+2H]^{2+}$) 597.3191 found 597.3186.

Synthesis of (Adm)2TbK (31).
The diamine **77** (50 mg, 0.08 mmol) was oxidized according to the general procedure described above for the oxidation of **64**. The crude product was purified by SiO_2 column chromatography using chloroform as eluent to provide pure (Adm)2TbK **(31)** (23 mg, 46 %) as a pale red solid. X-band EPR spectrum (293 K, in CH_2Cl_2): triplet, A_N = 1.53 mT. ESI-MS *m/z* = 625 $[M+H]^+$; 642 $[M+NH_4]^+$; 647 $[M+Na]^+$.

Synthesis of bTbK-d₂₄ (28).
The diamine **79** (100 mg, 0.23 mmol) was oxidized according to the general procedure described above for the oxidation of **64**. The crude product was purified by SiO_2 column chromatography using CH_2Cl_2/EtOH (99/1) as eluent to provide bTbK-d_{24} **(28)** (90 mg, 84 %) as a pale red solid. mp 180-182°C. X-band EPR spectrum (293 K, in CH_2Cl_2): triplet, A_N = 1.55 mT. ESI-MS *m/z* = 465 $[M+H]^+$. HRMS-ESI calcd for $C_{23}H_{17}D_{24}N_2O_6^{2\cdot}$ ($[M+H]^+$) 465.4466 found 465.4460. ^{1}H NMR of reduced ***bTbK-d₂₄*** ($CDCl_3$) δ 2.33 (s, 8H), 3.71 (s, 8H).

NMR spectra

When reported, NMR spectra of biradicals were obtained after adding a drop of phenylhydrazine directly in the NMR tubes.

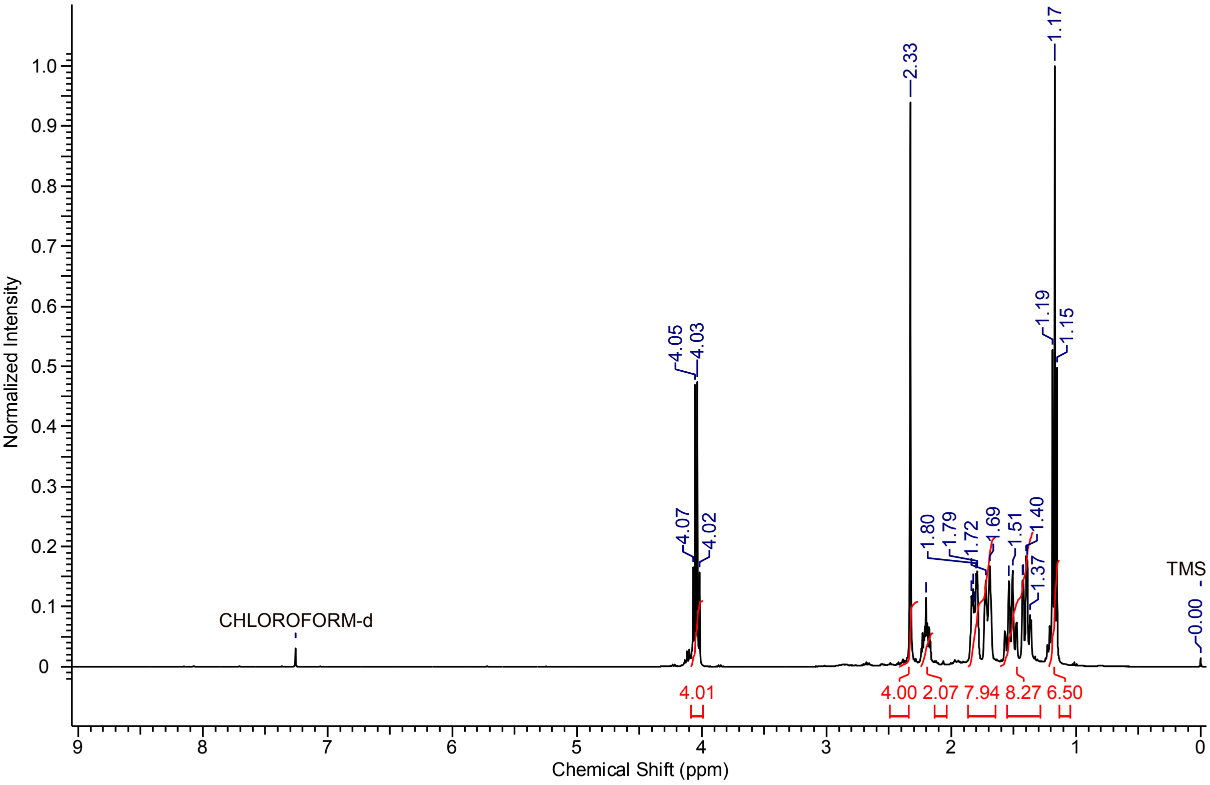

^{1}H NMR ($CDCl_3$, 300 MHz) spectrum of **51**.

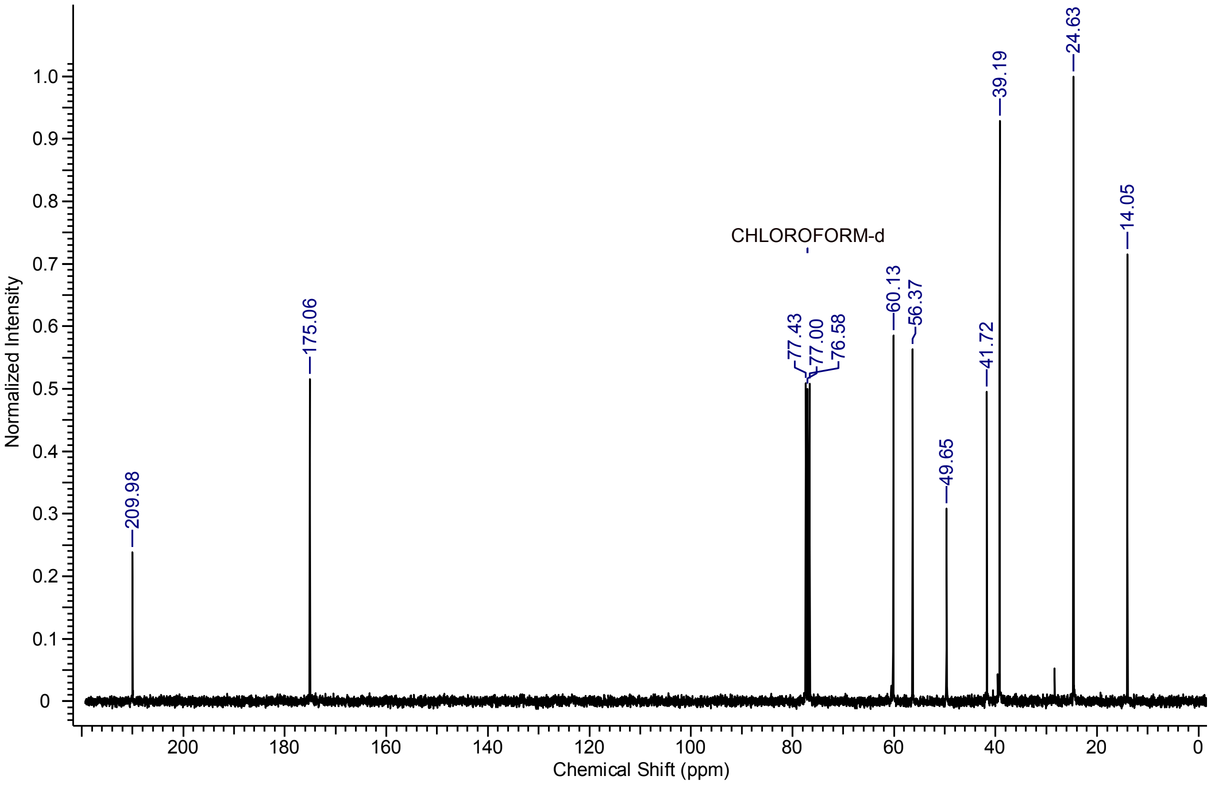

^{13}C NMR ($CDCl_3$, 75 MHz) spectrum of **51**.

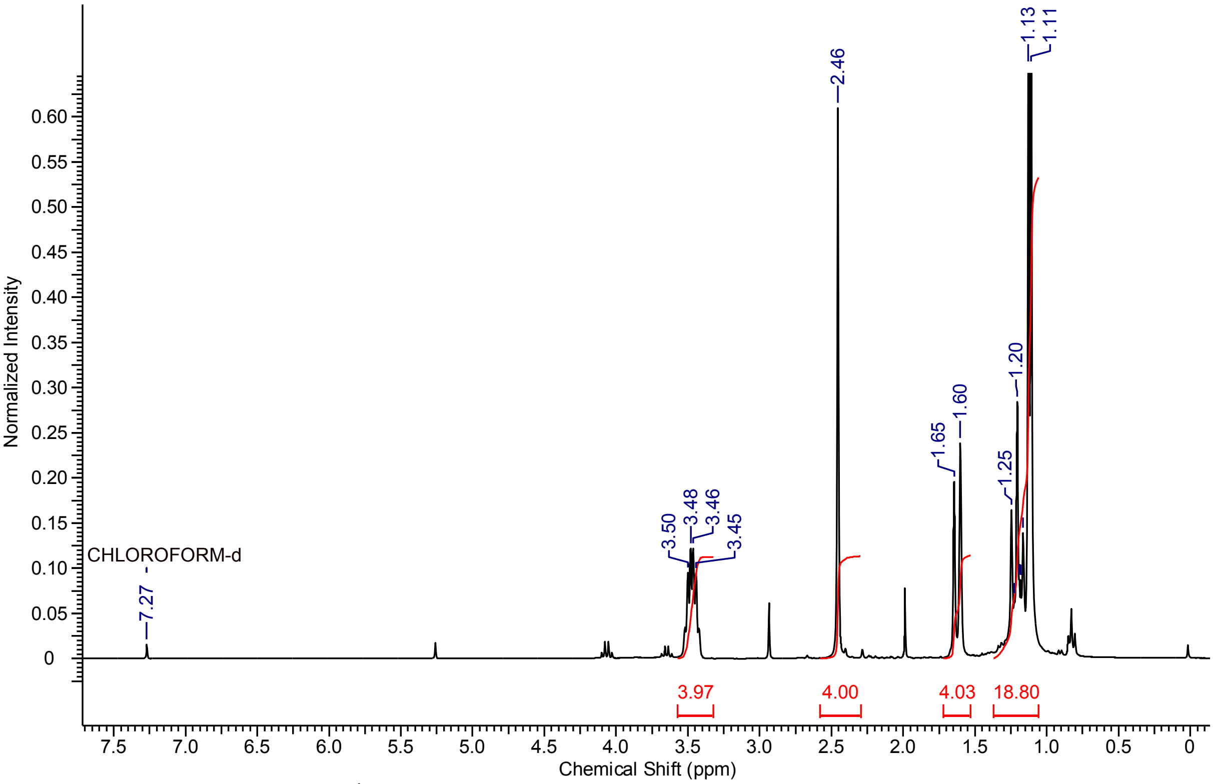

^{1}H NMR ($CDCl_3$, 300 MHz) spectrum of **60**.

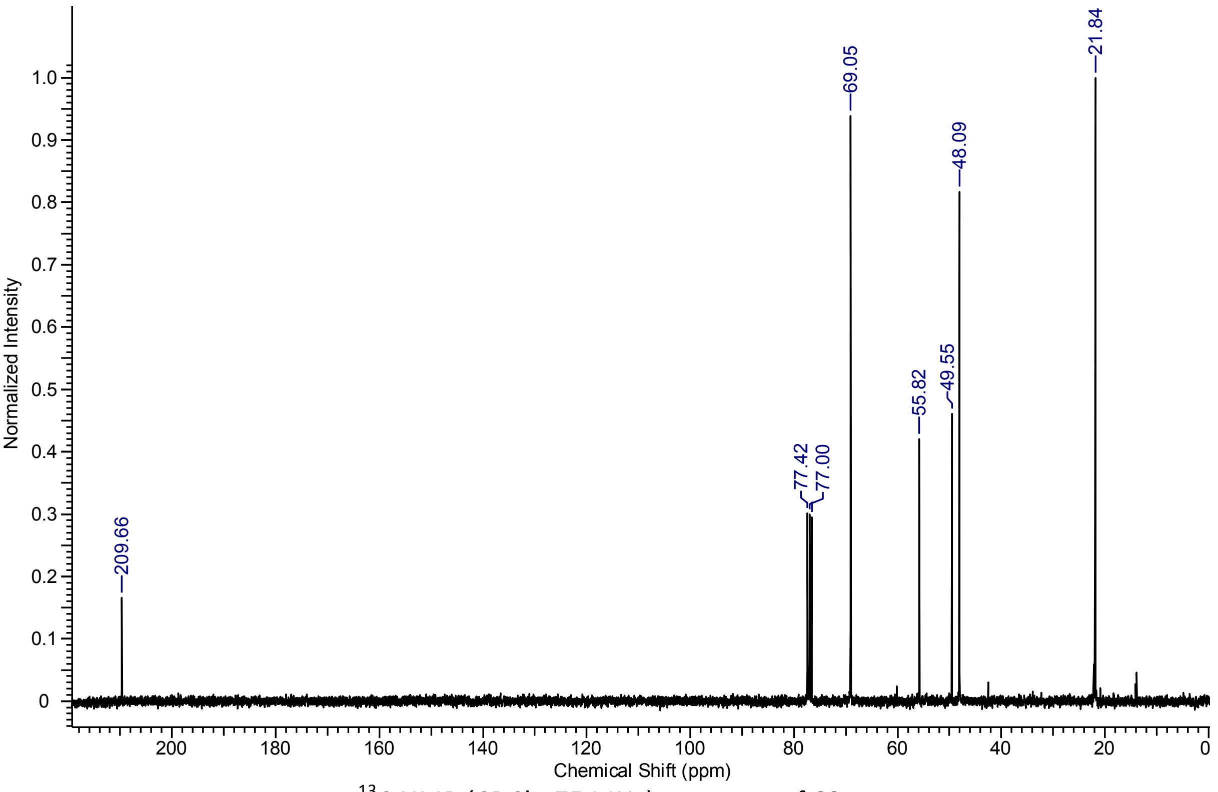

^{13}C NMR ($CDCl_3$, 75 MHz) spectrum of **60**.

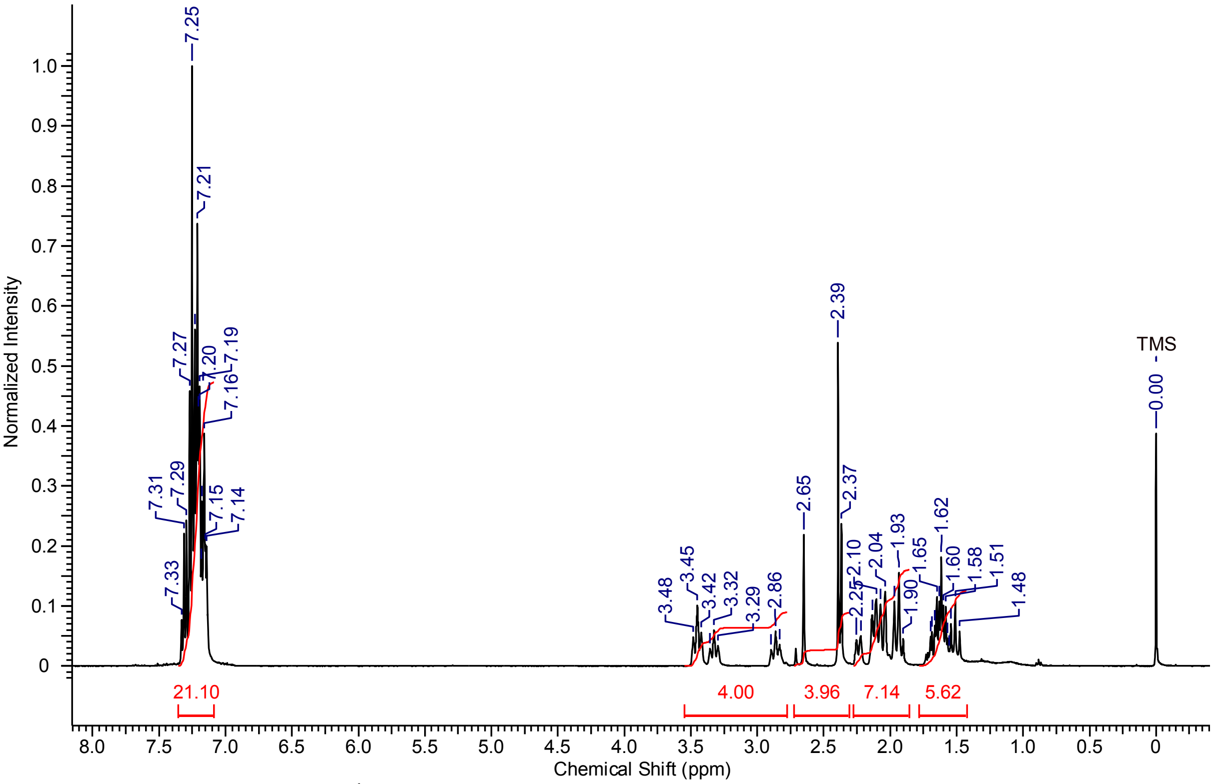

^{1}H NMR ($CDCl_3$, 300 MHz) spectrum of **62**.

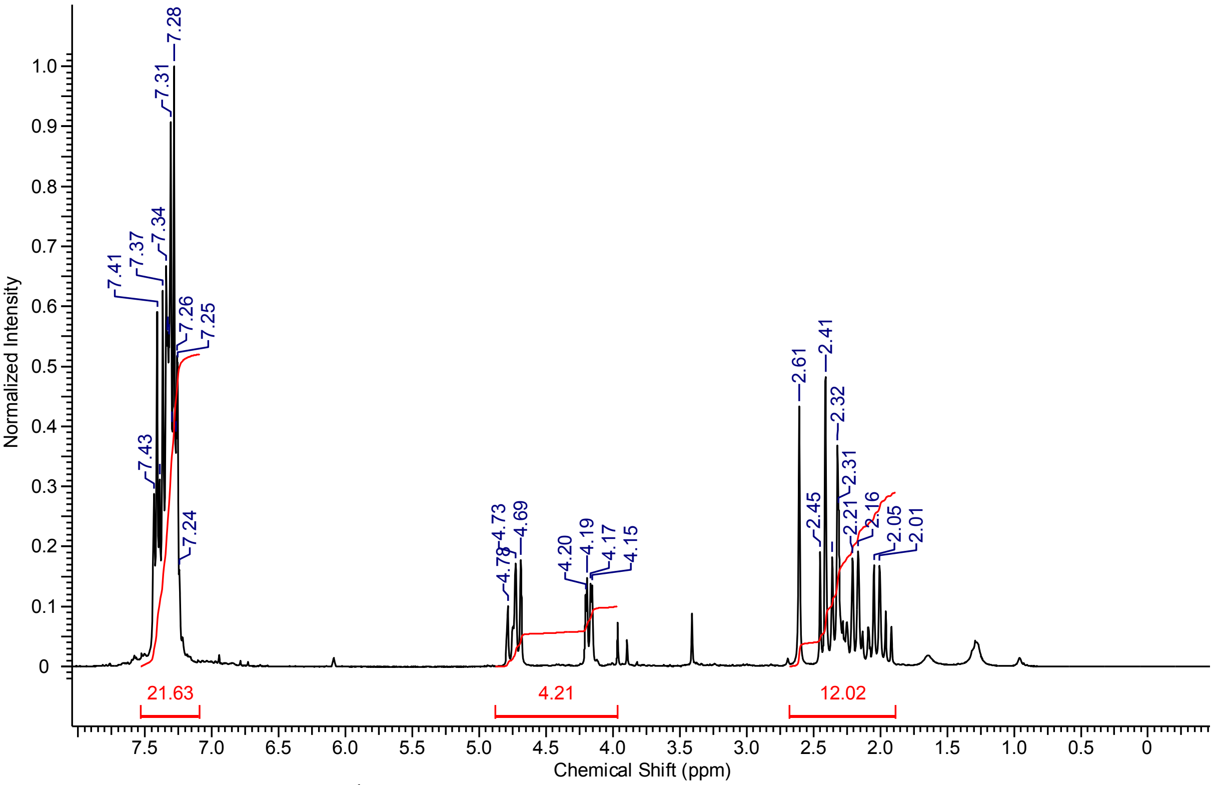

^{1}H NMR ($CDCl_3$, 300 MHz) spectrum of **63**.

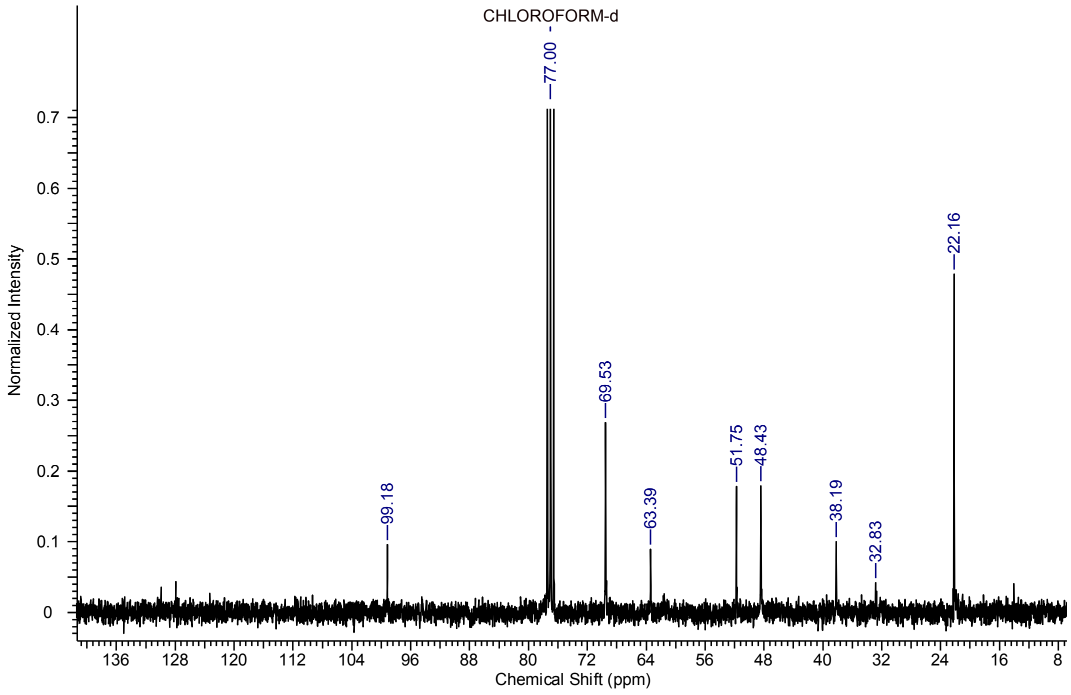

^{13}C NMR ($CDCl_3$, 75 MHz) spectrum of **64**.

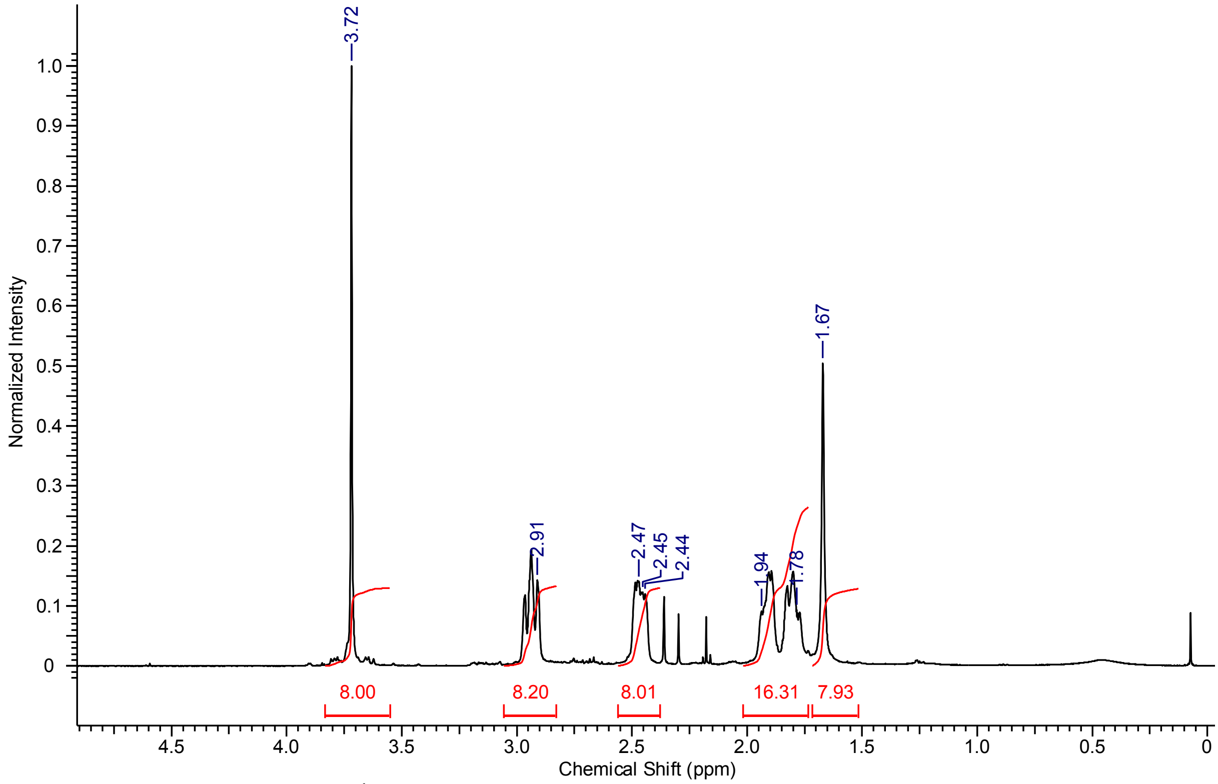

^{1}H NMR ($CDCl_3$, 300 MHz) spectrum of **65**.

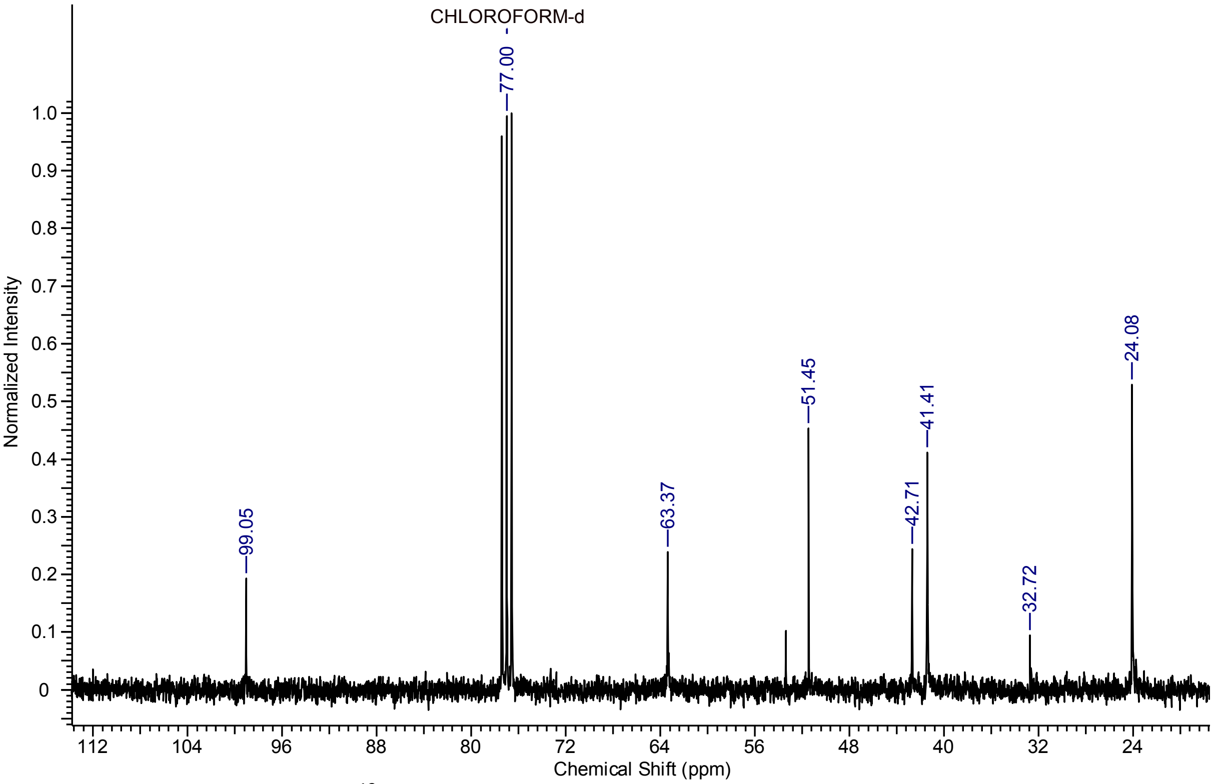

^{13}C NMR ($CDCl_3$, 75 MHz) spectrum of **65**.

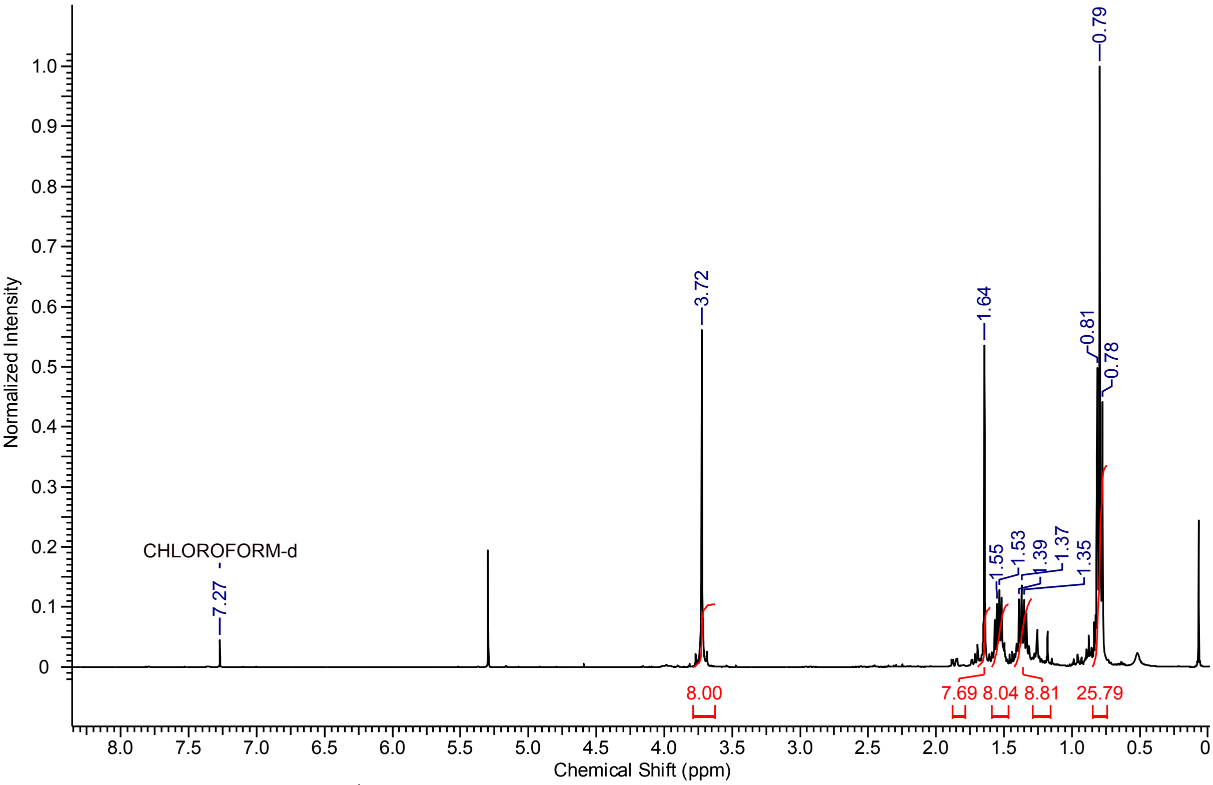

^{1}H NMR ($CDCl_3$, 300 MHz) spectrum of **68**.

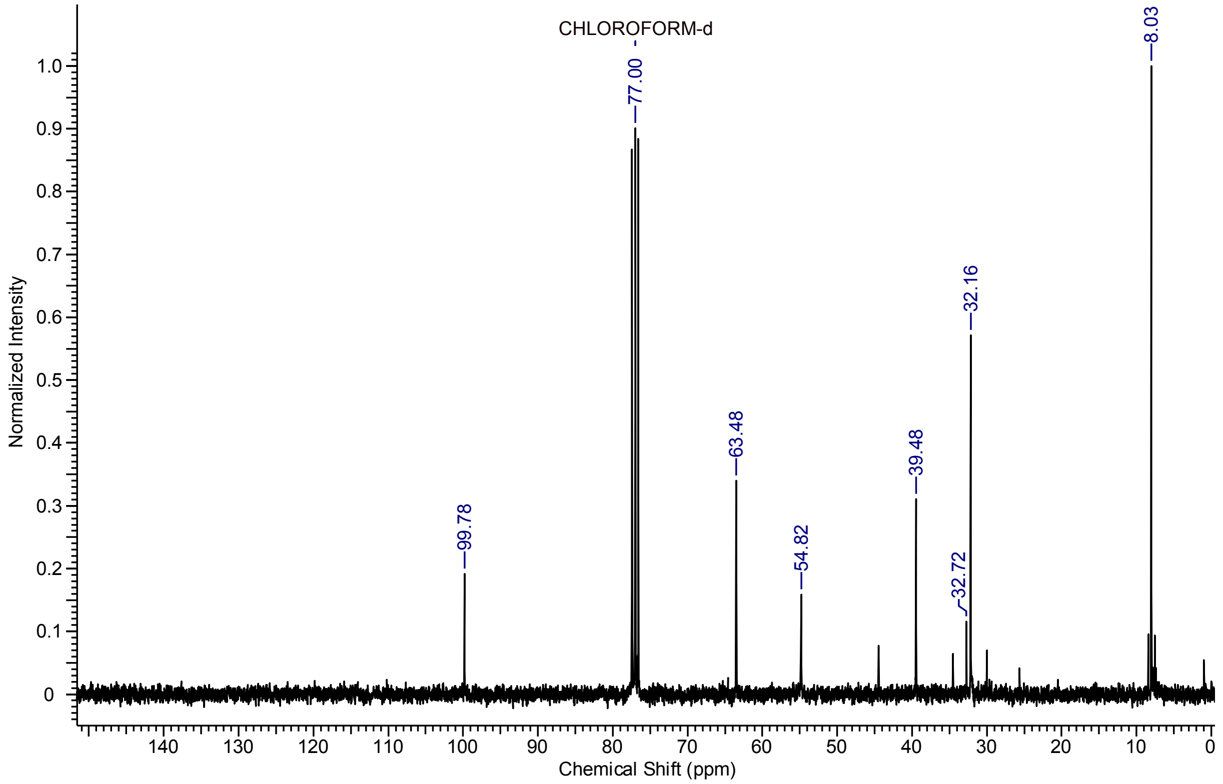

^{13}C NMR ($CDCl_3$, 75 MHz) spectrum of **68**.

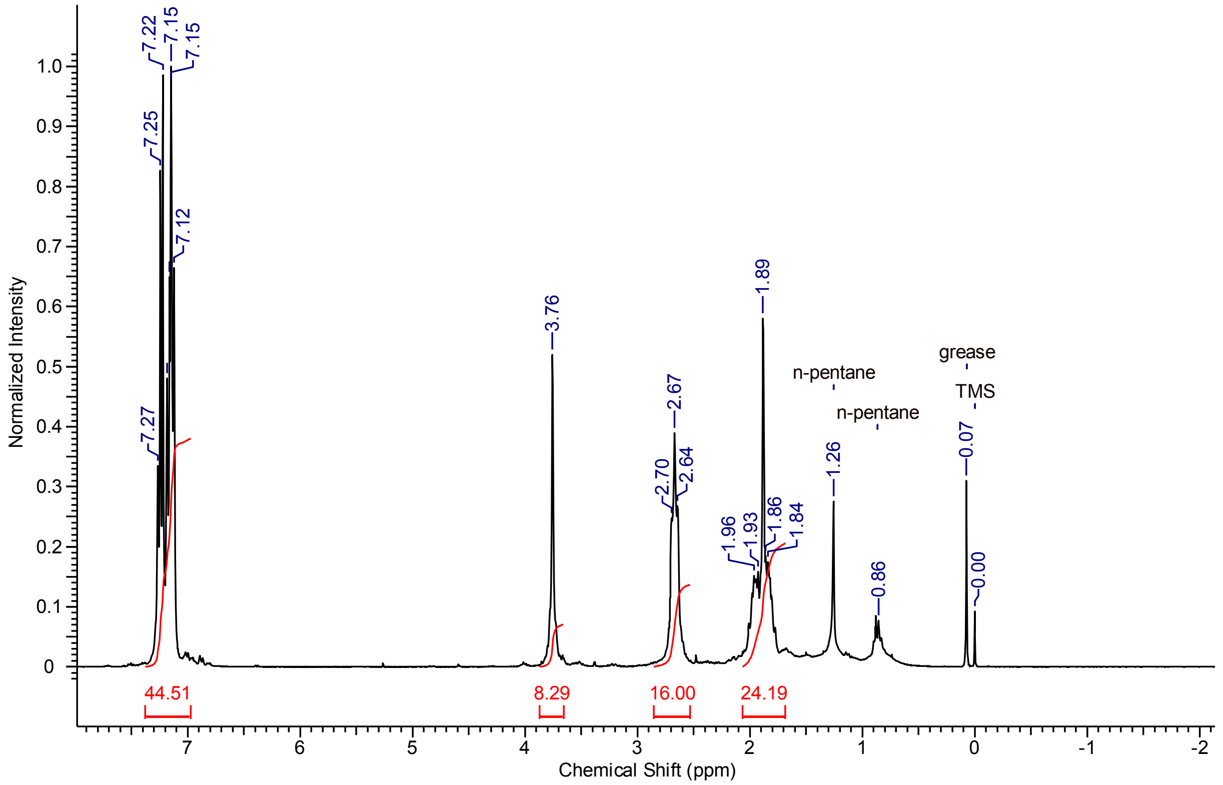

^{1}H NMR ($CDCl_3$, 300 MHz) spectrum of **69**.

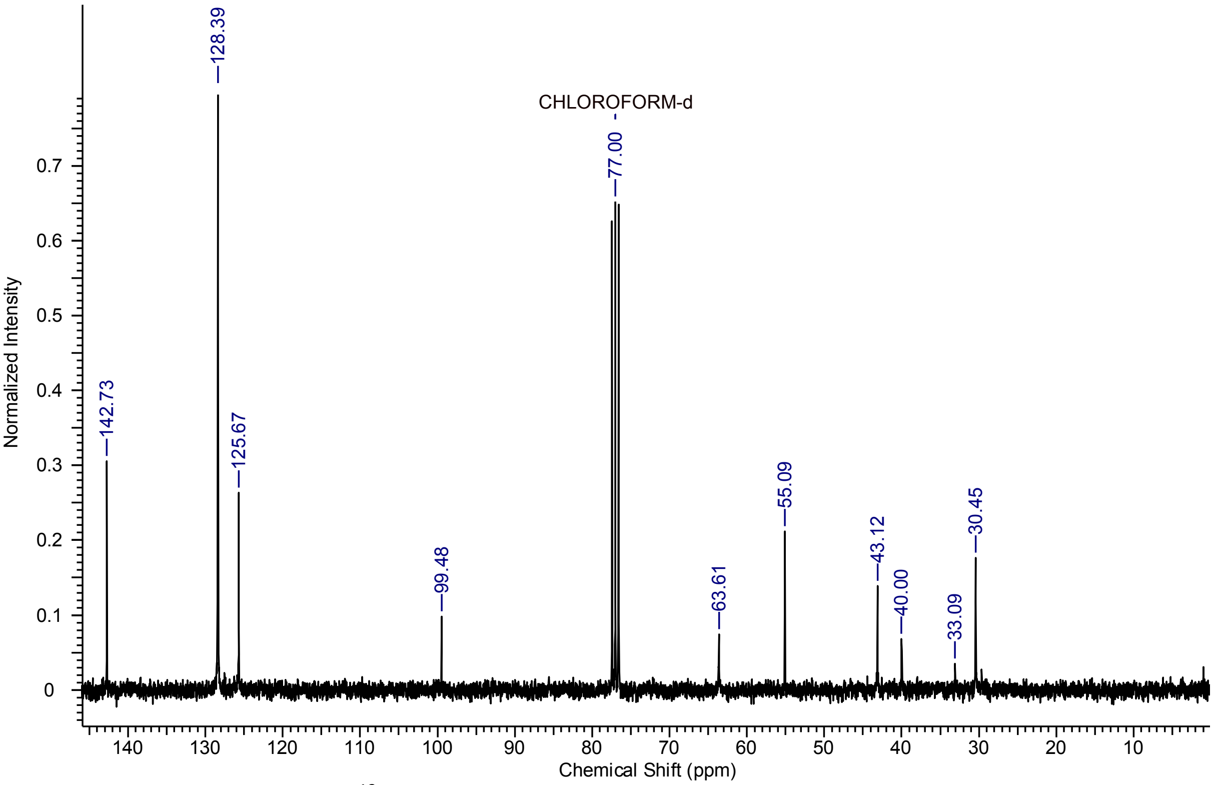

^{13}C NMR ($CDCl_3$, 75 MHz) spectrum of **69**.

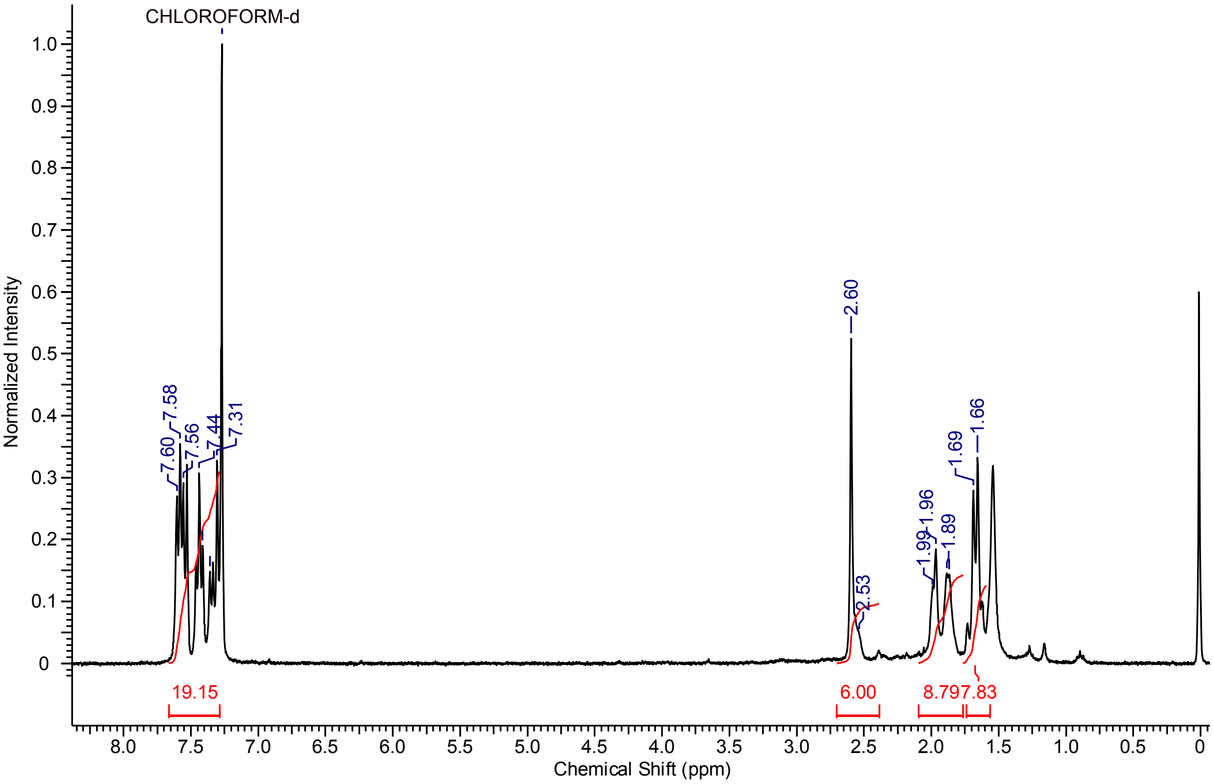

^{1}H NMR ($CDCl_3$, 300 MHz) spectrum of **70**.

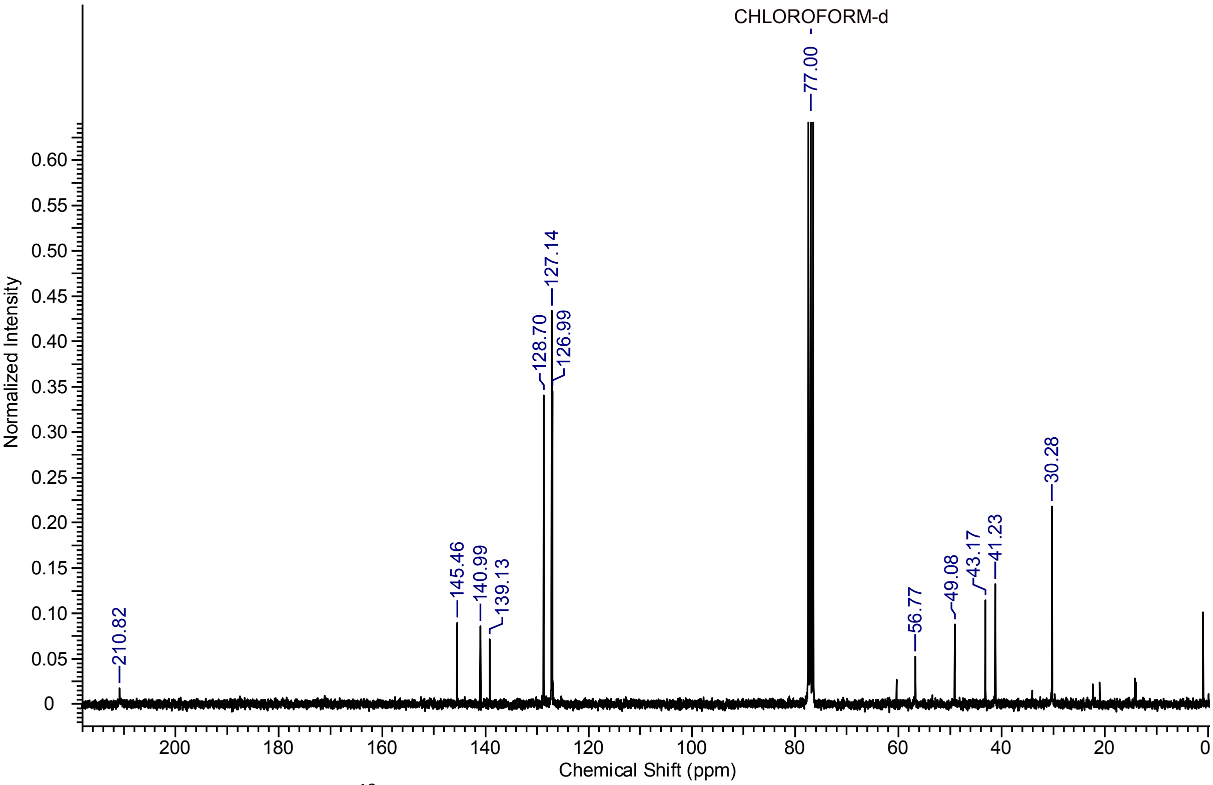

^{13}C NMR ($CDCl_3$, 75 MHz) spectrum of **70**.

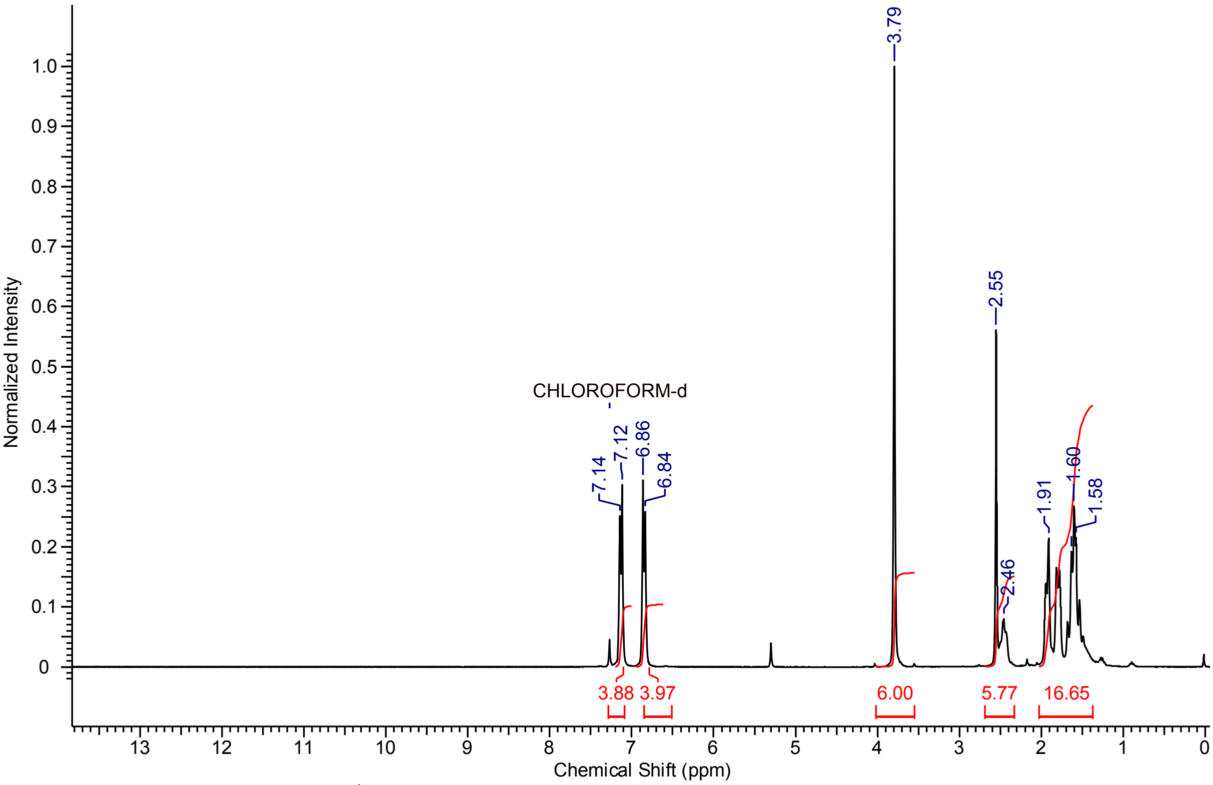

^{1}H NMR ($CDCl_3$, 300 MHz) spectrum of **71**.

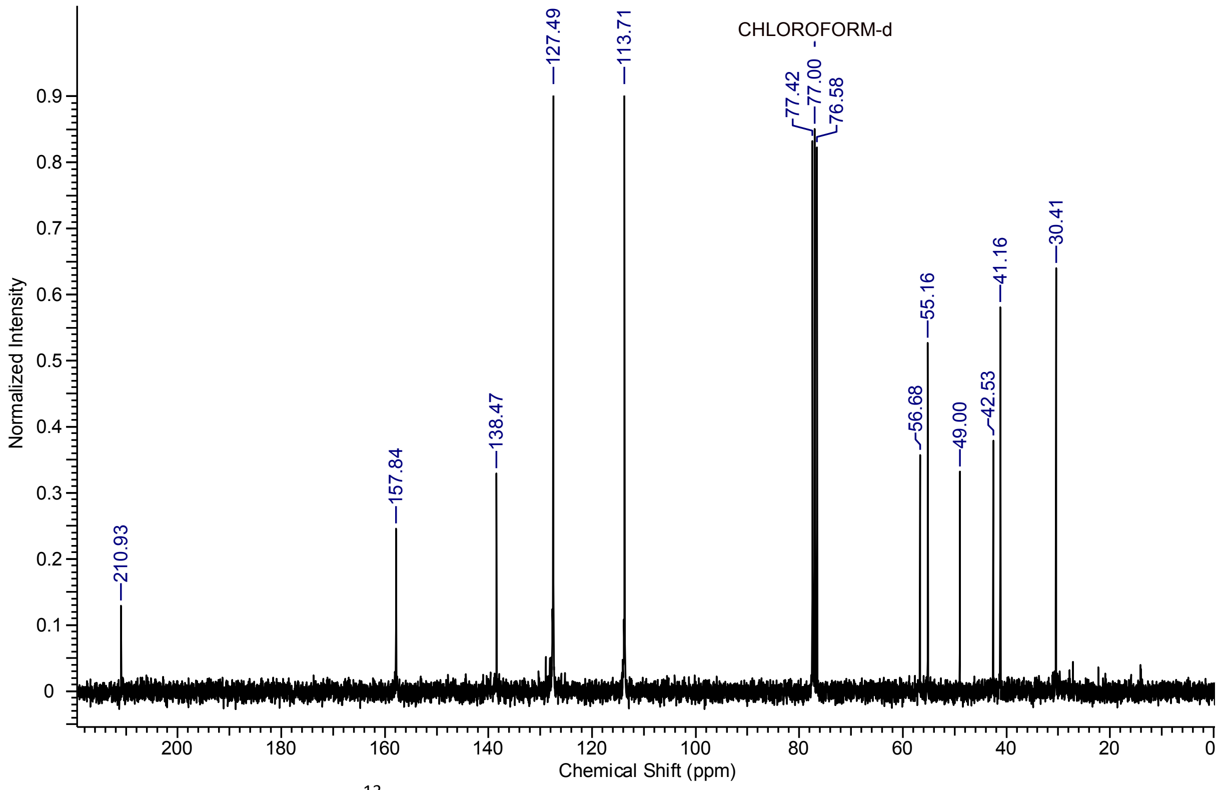

^{13}C NMR ($CDCl_3$, 75 MHz) spectrum of **71**.

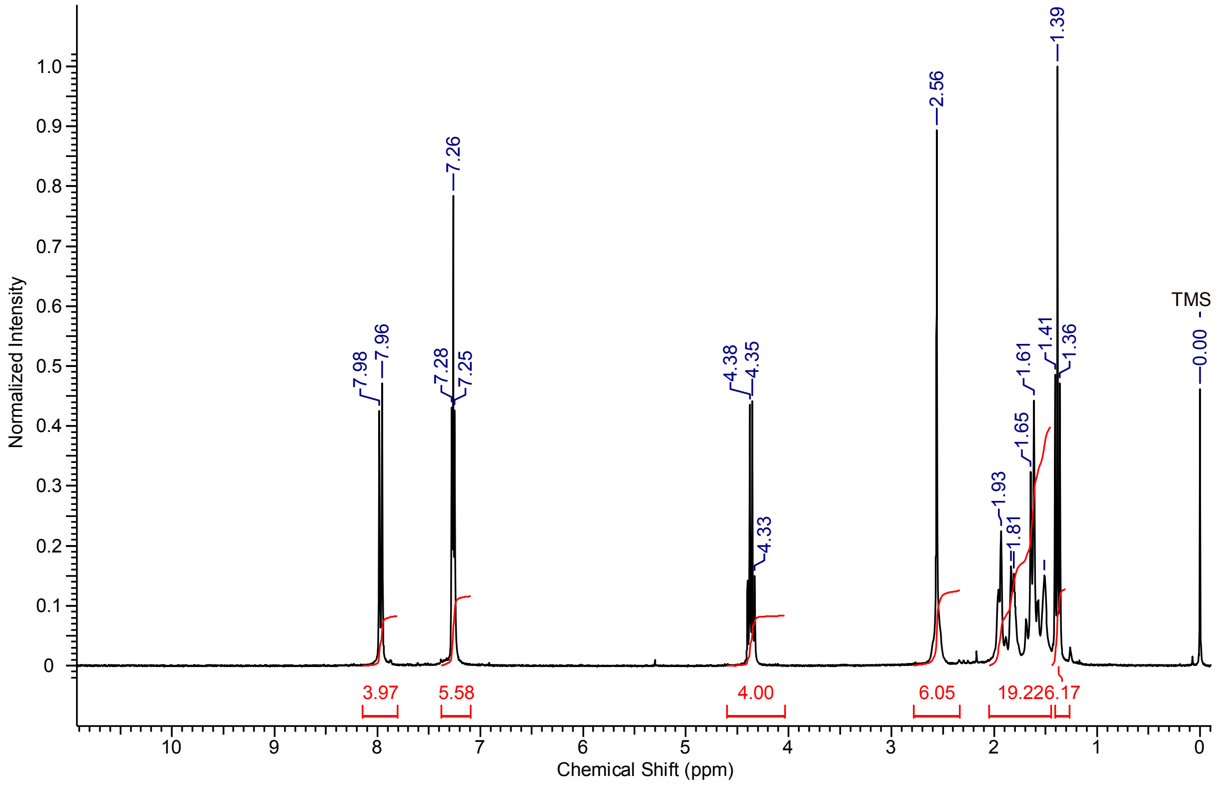

^{1}H NMR ($CDCl_3$, 300 MHz) spectrum of **71**.

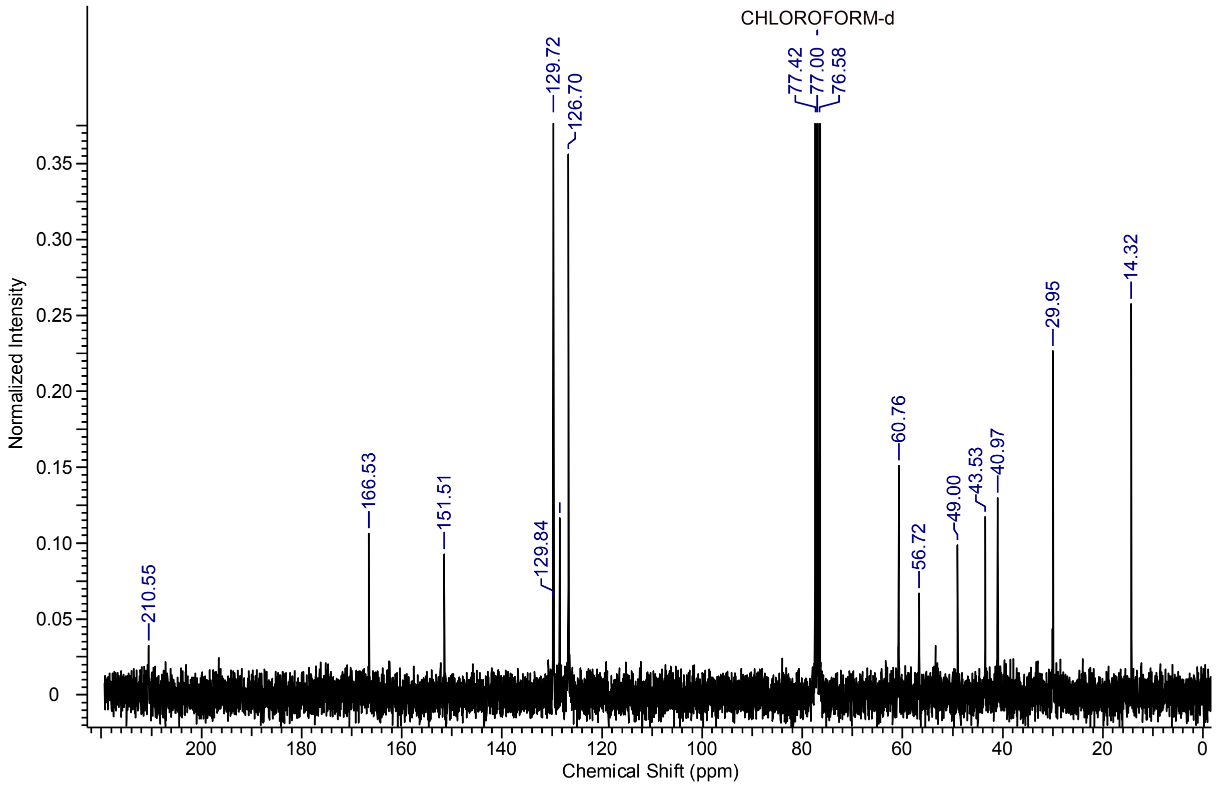

^{13}C NMR ($CDCl_3$, 75 MHz) spectrum of **72**.

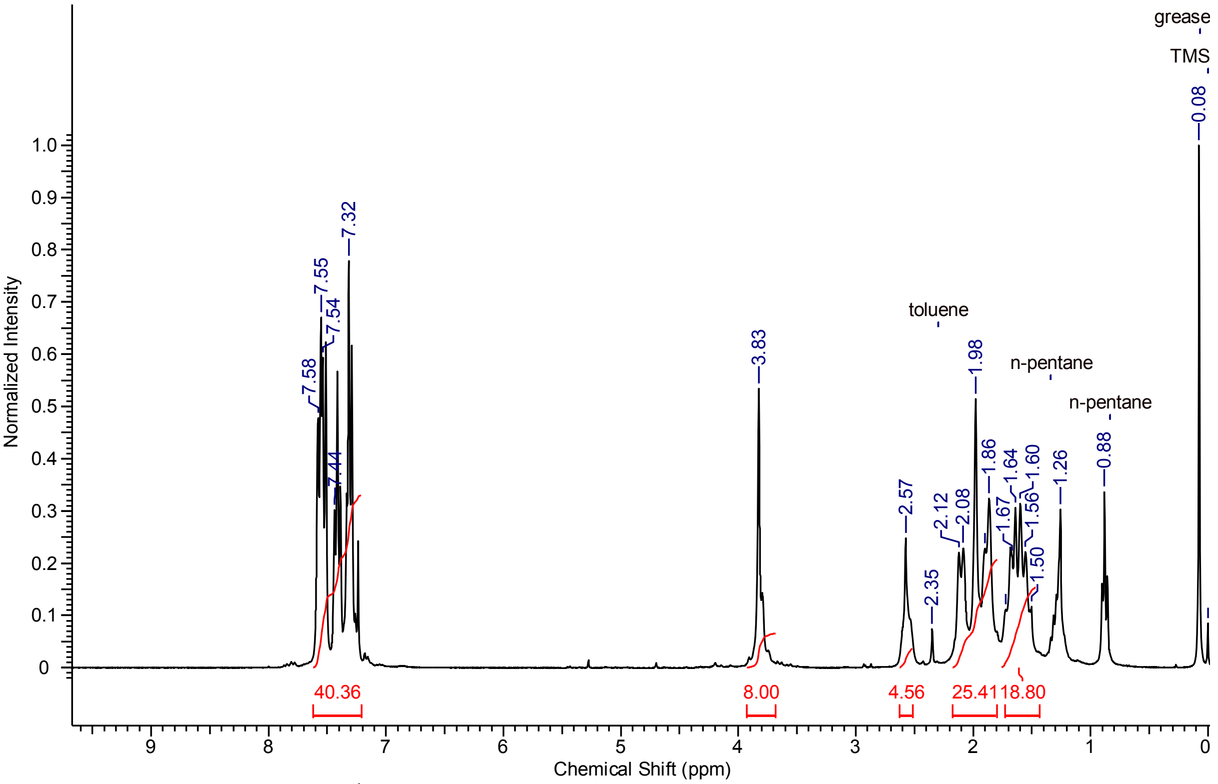

^{1}H NMR ($CDCl_3$, 300 MHz) spectrum of **73**.

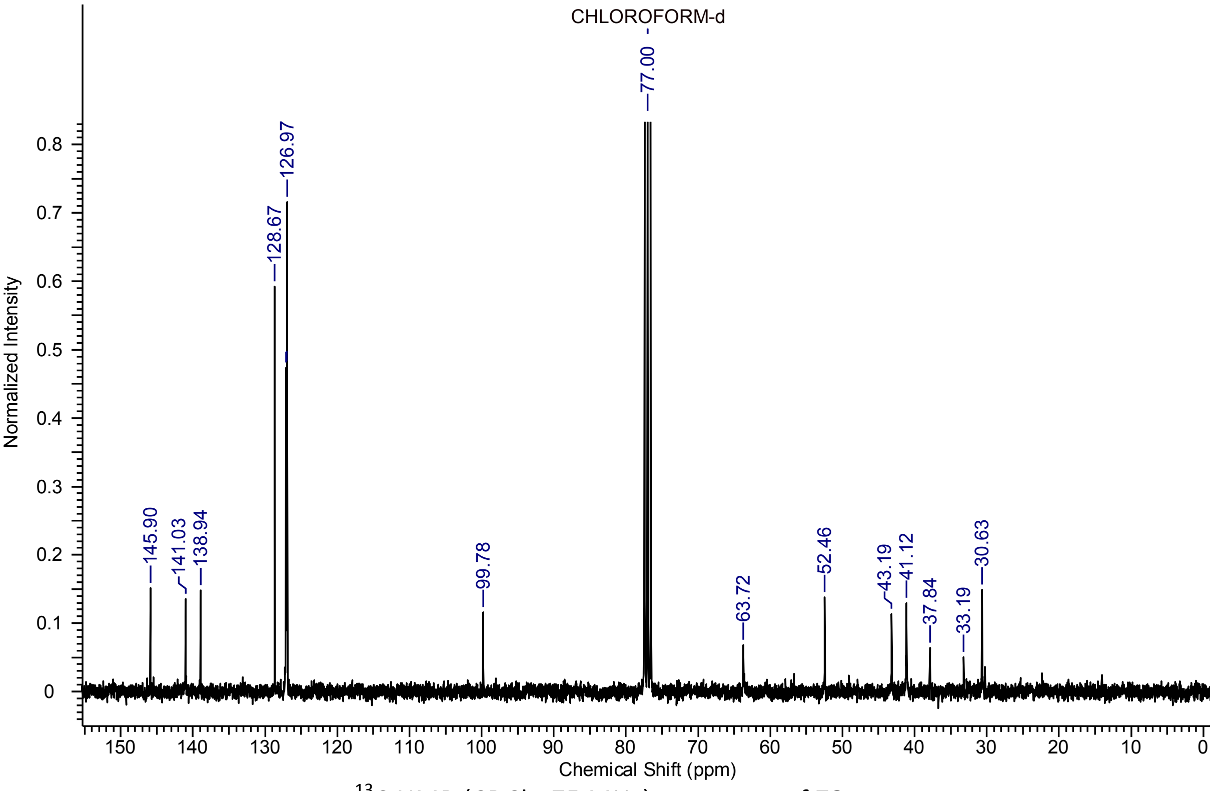

^{13}C NMR ($CDCl_3$, 75 MHz) spectrum of **73**.

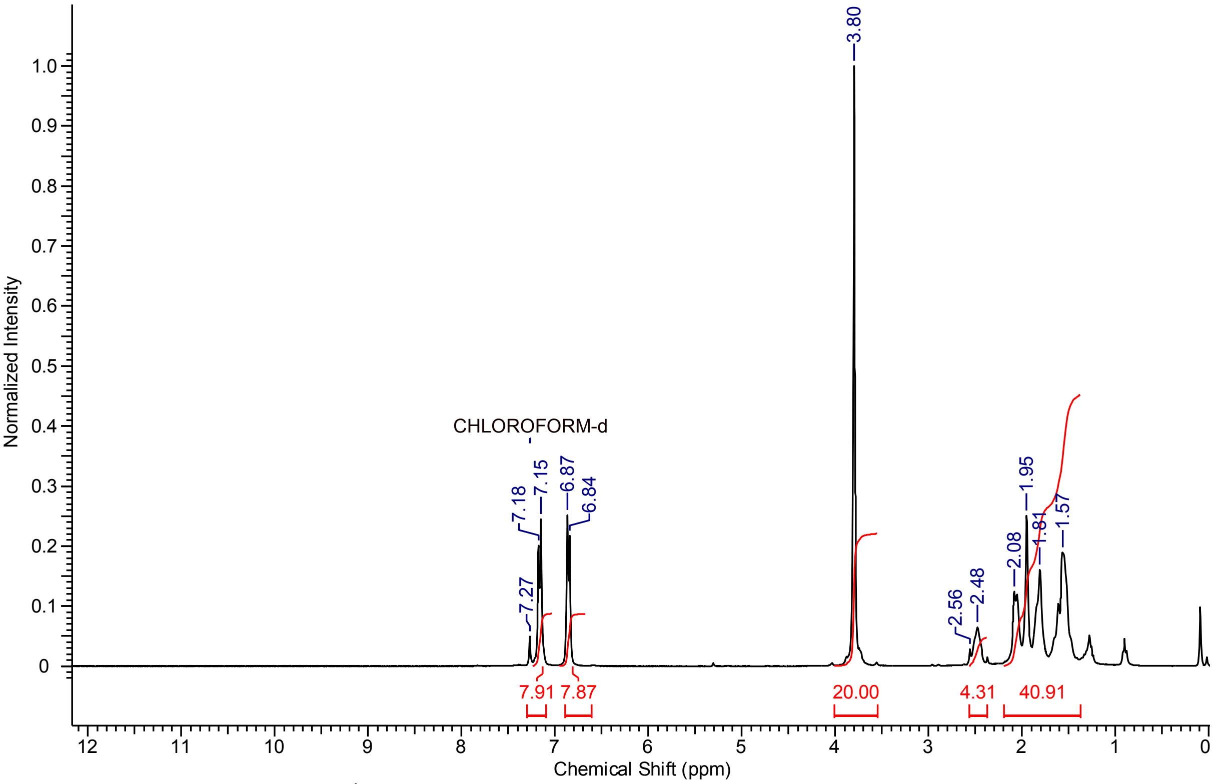

^{1}H NMR ($CDCl_3$, 300 MHz) spectrum of **74**.

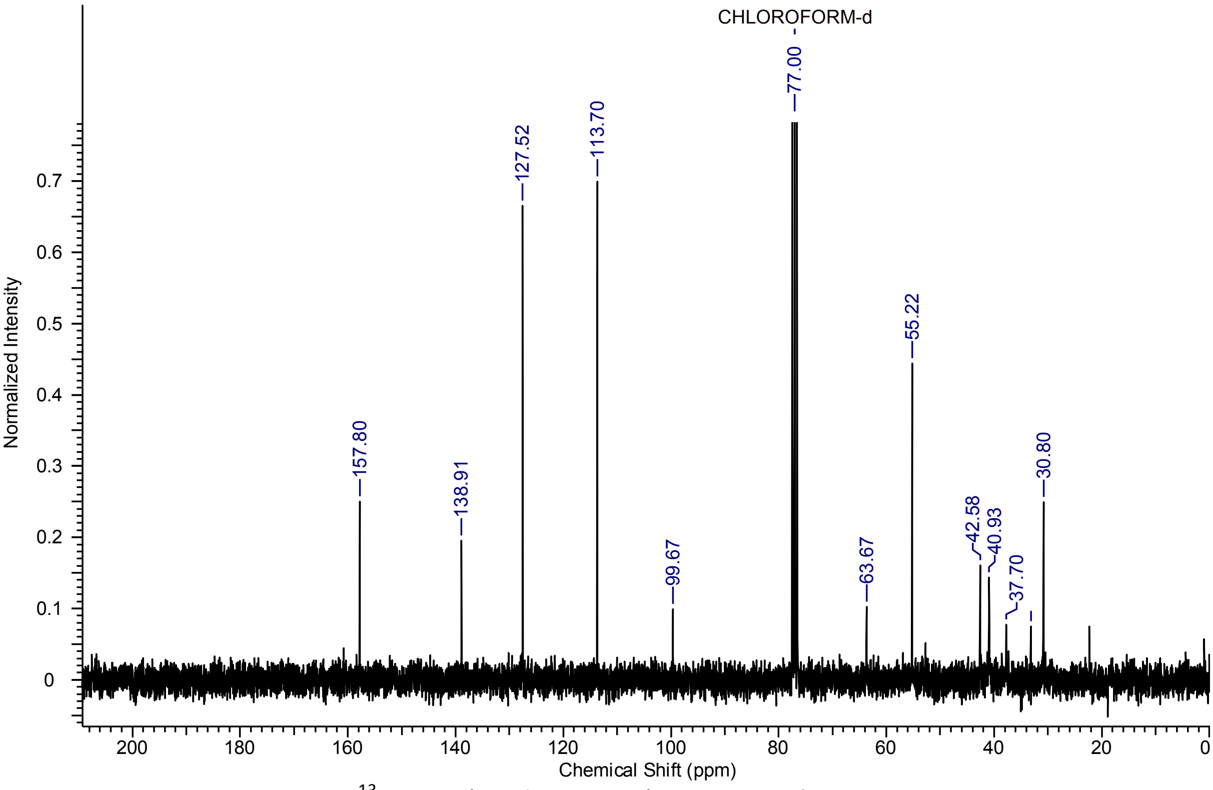

^{13}C NMR ($CDCl_3$, 75 MHz) spectrum of **74**.

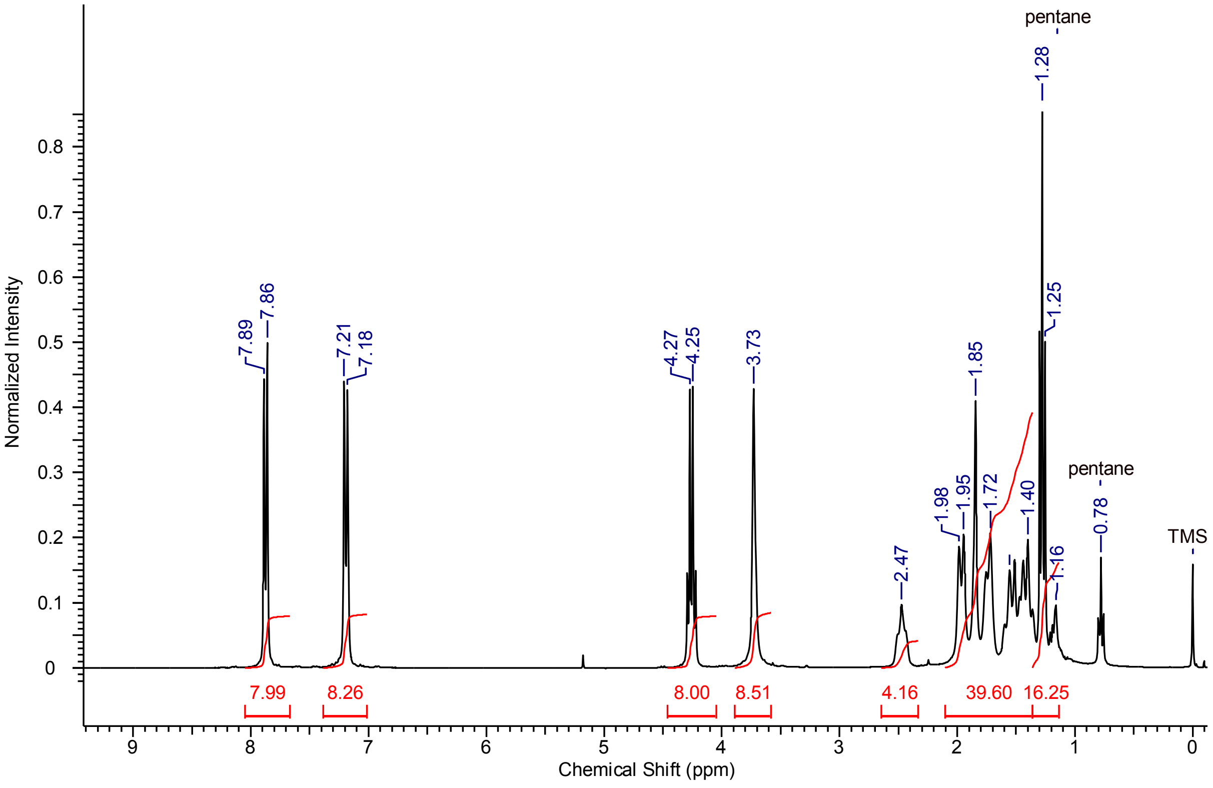

^{1}H NMR ($CDCl_3$, 300 MHz) spectrum of **75**.

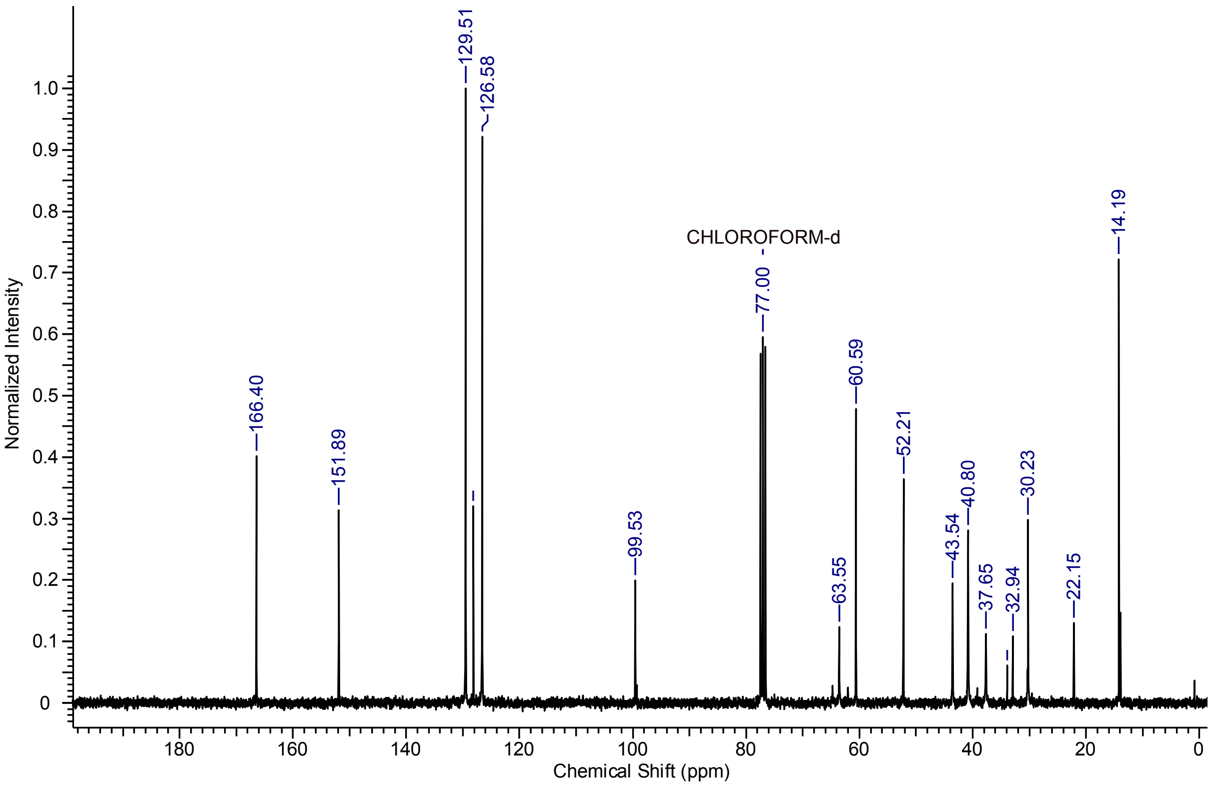

^{13}C NMR ($CDCl_3$, 75 MHz) spectrum of **75**.

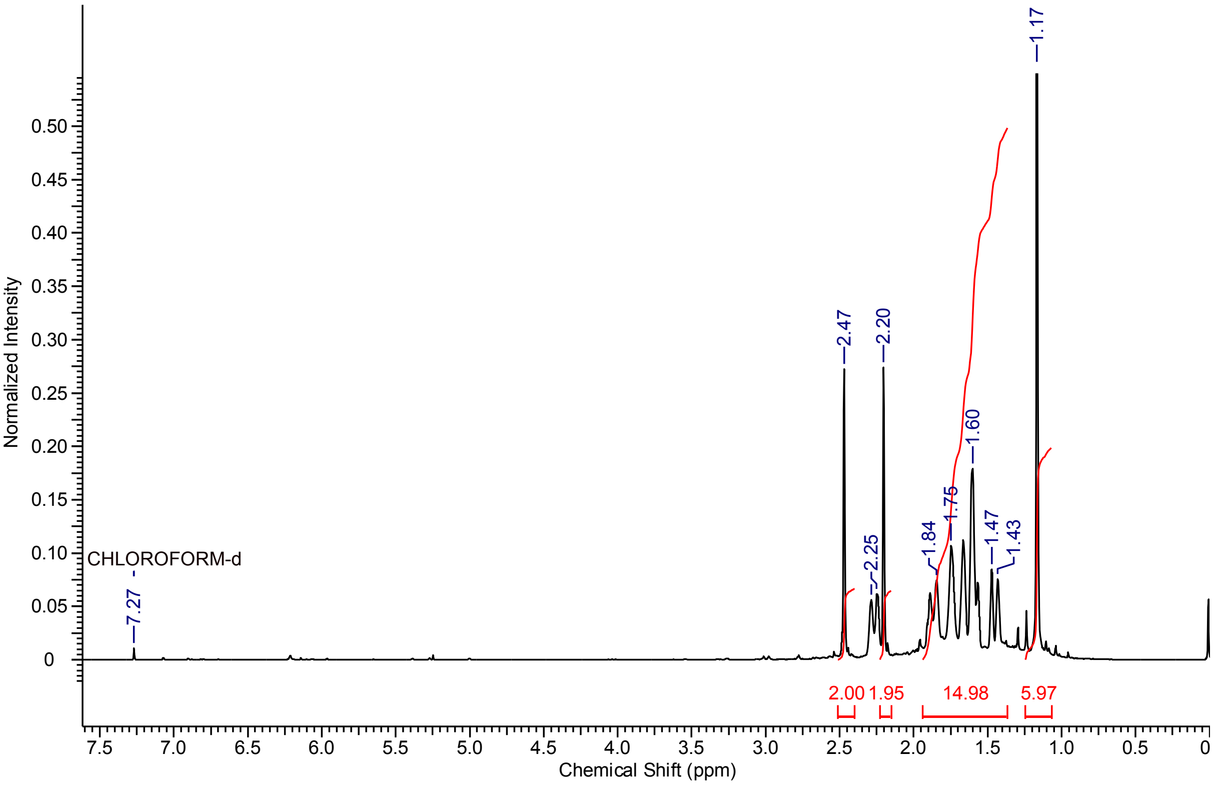

^{1}H NMR ($CDCl_3$, 300 MHz) spectrum of **76**.

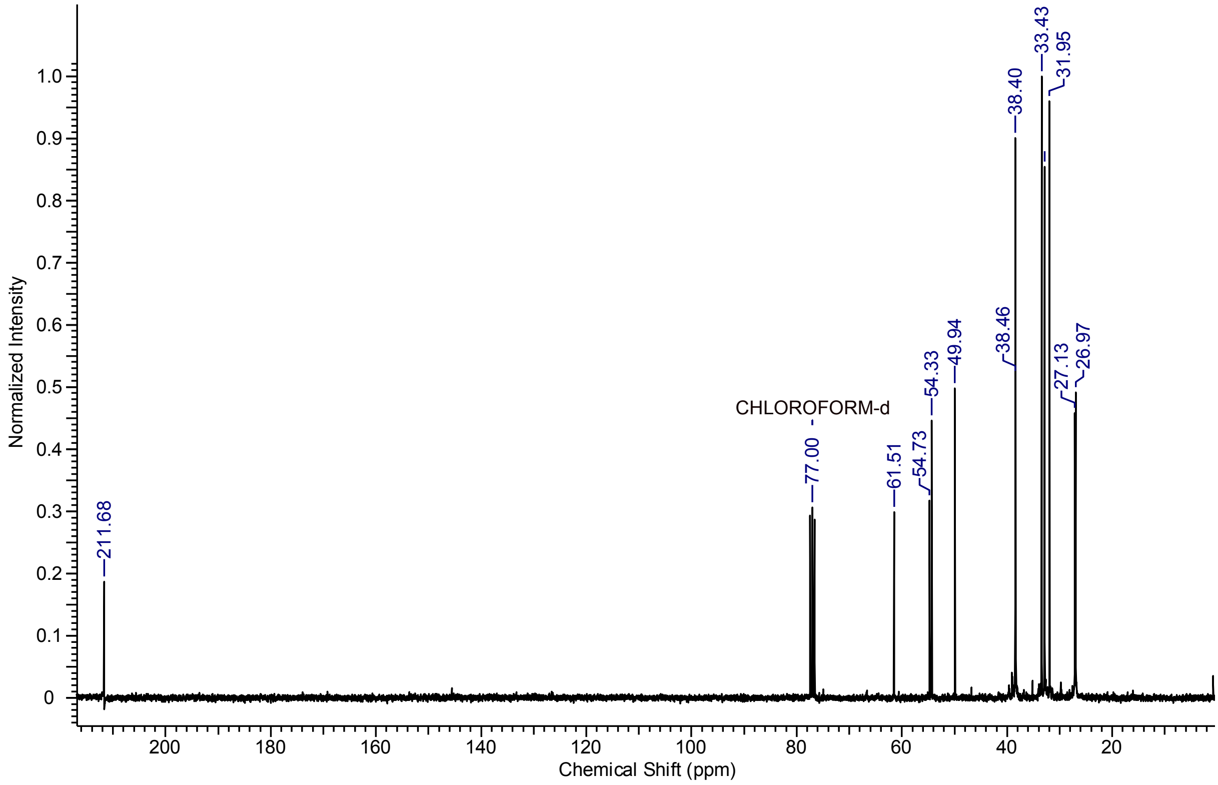

^{13}C NMR ($CDCl_3$, 75 MHz) spectrum of **76**.

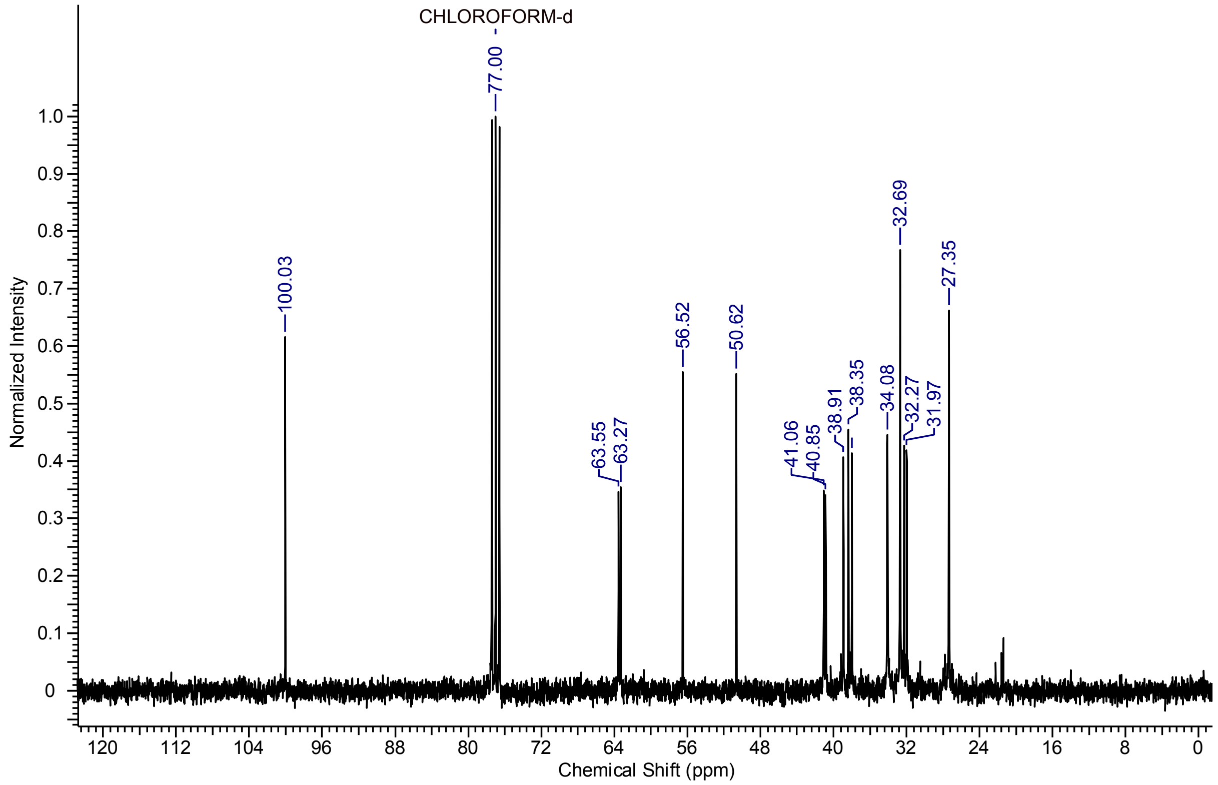

^{13}C NMR ($CDCl_3$, 75 MHz) spectrum of **77**.

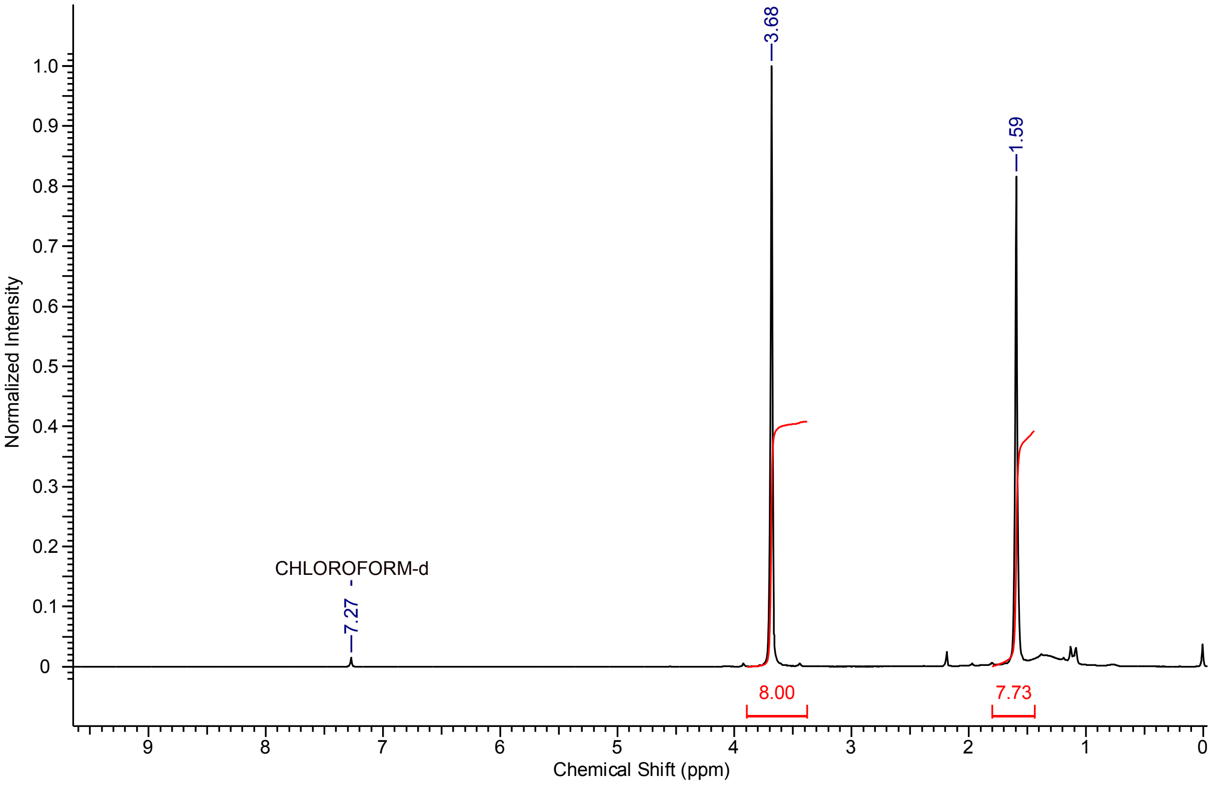

^{1}H NMR ($CDCl_3$, 300 MHz) spectrum of **79**.

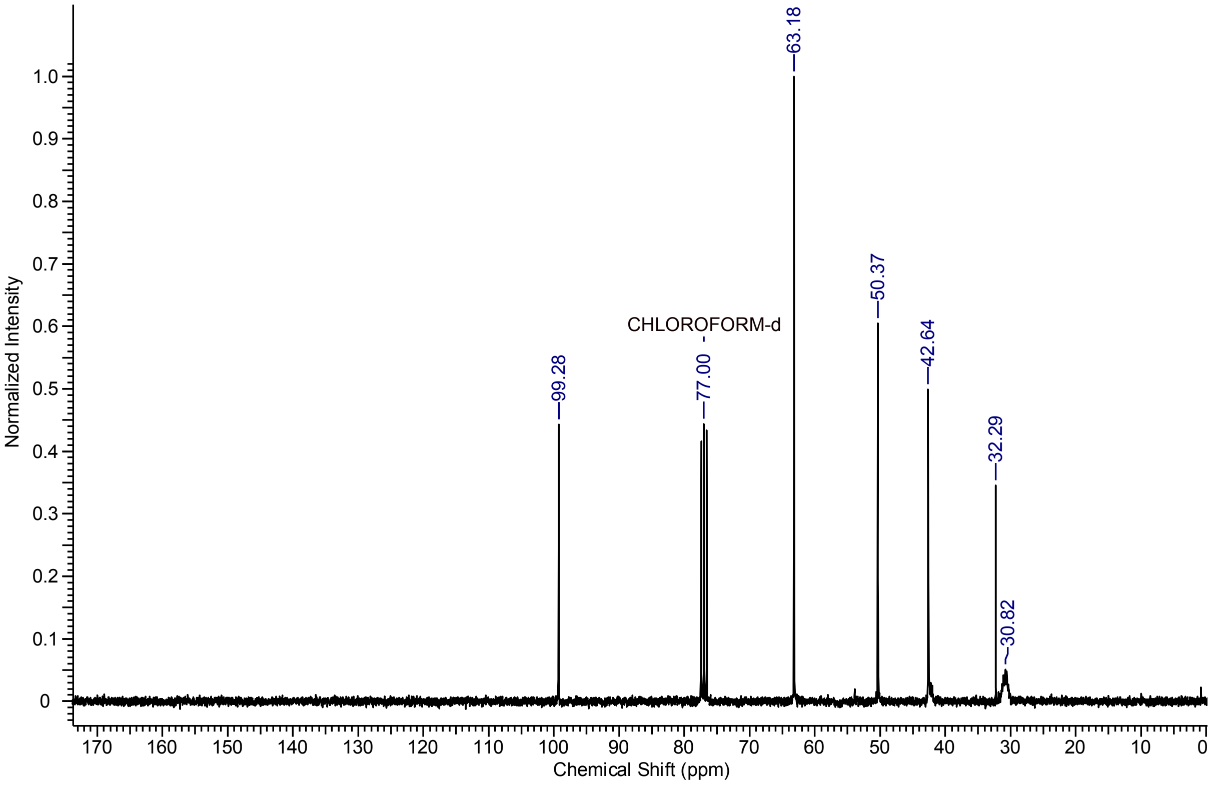

^{13}C NMR ($CDCl_3$, 75 MHz) spectrum of **79**.

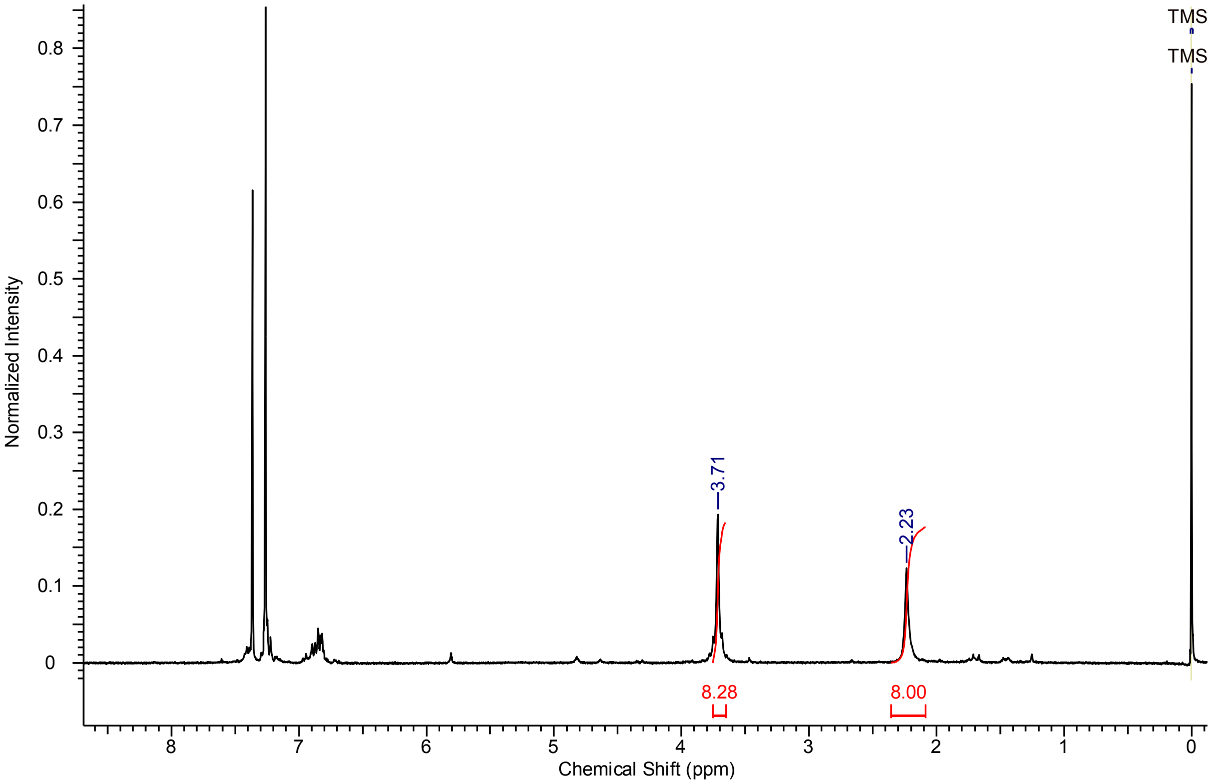

^{1}H NMR ($CDCl_3$, 300 MHz) spectrum of ***bTbK-d₂₄* (28)** after addition of a drop of phenylhydrazine***.***

EPR spectra of reported dinitroxides

1 mM in TCE, 293 K, 9 GHz (X band) EPR

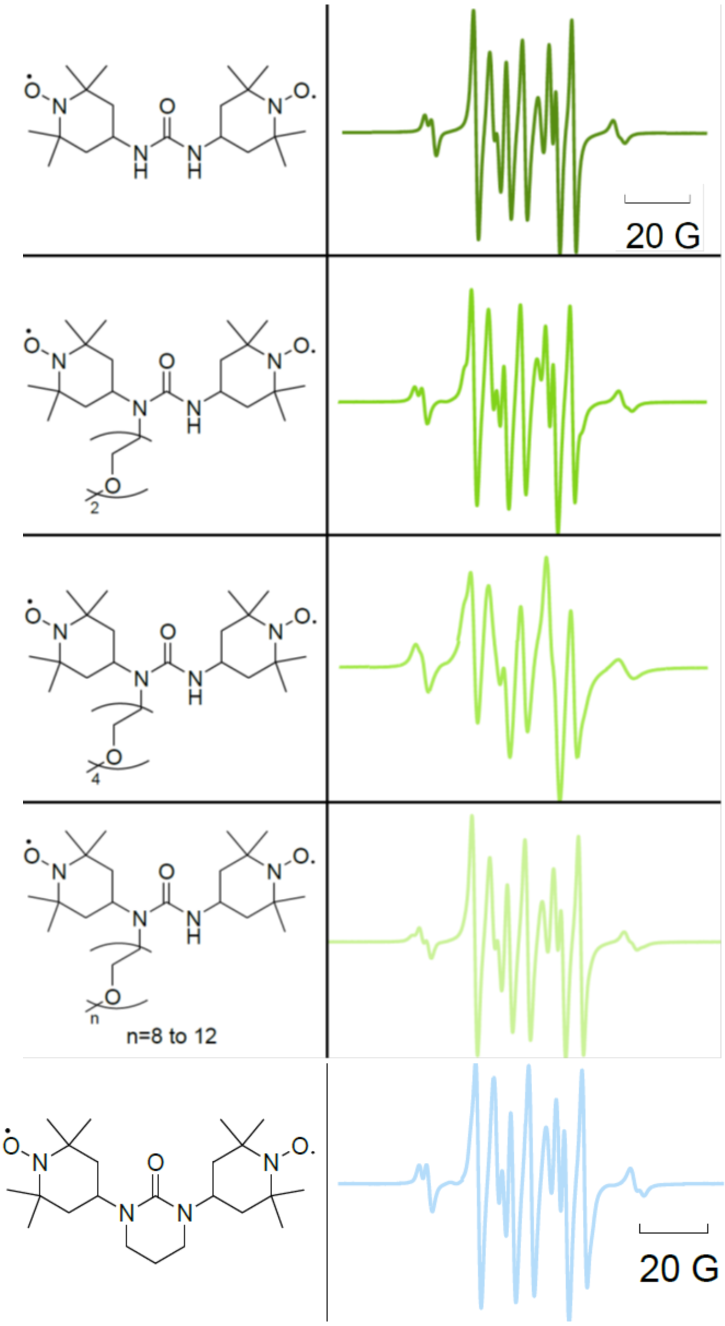

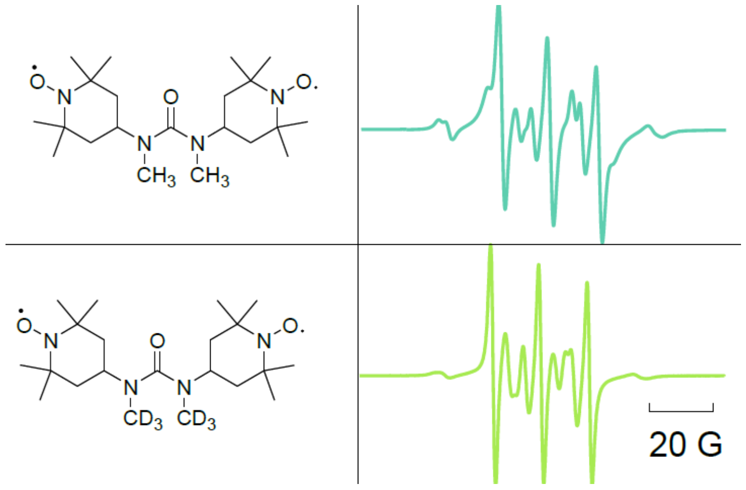
20 G

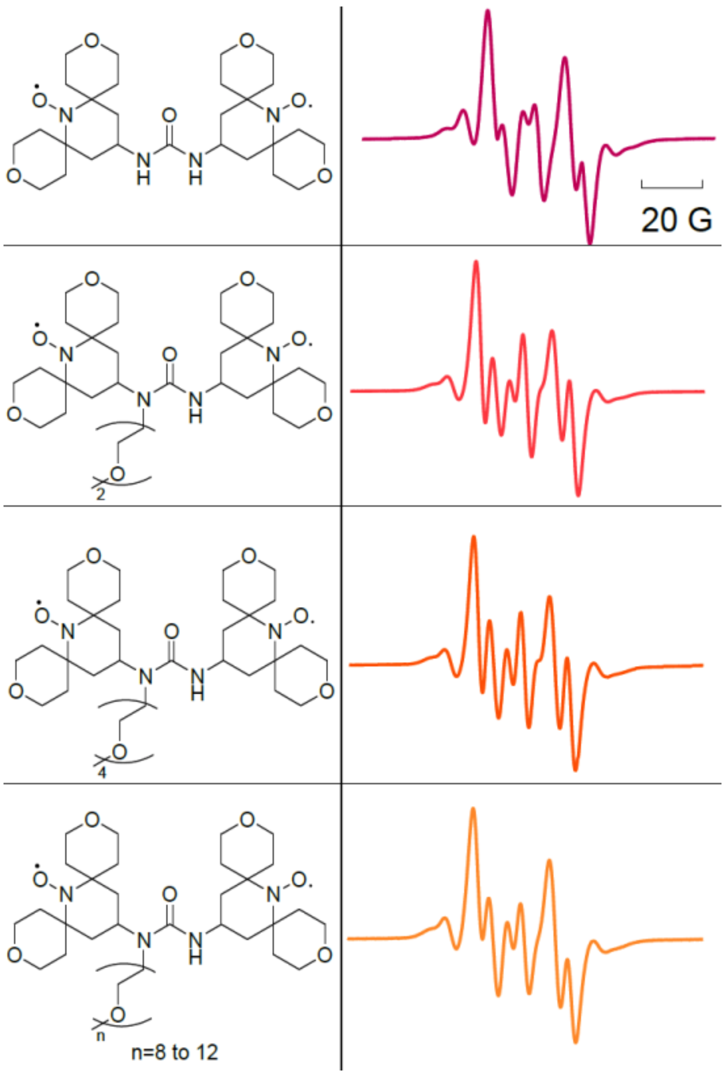
20 G
n=8 to 12

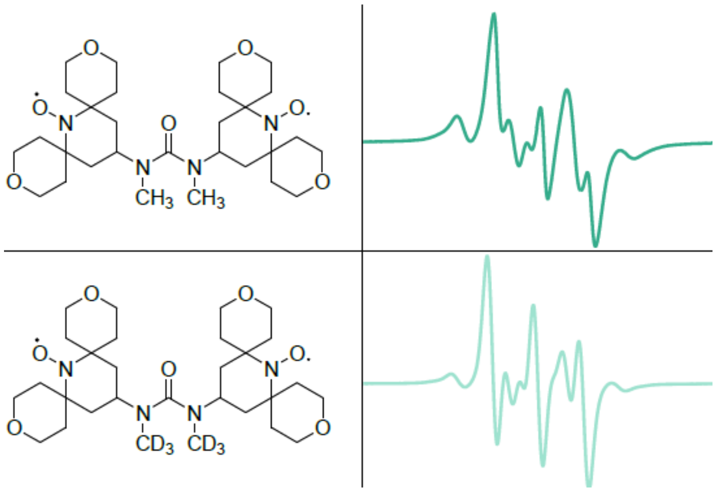

CH3 CH3
CD3 CD3

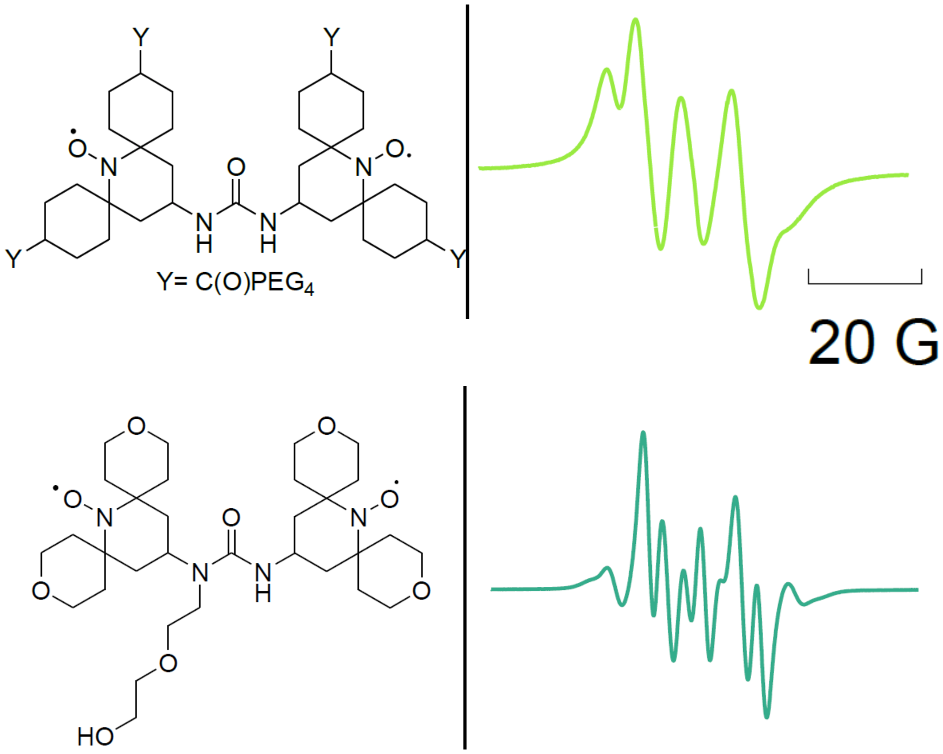

Y= C(O)PEG4
20 G
HO

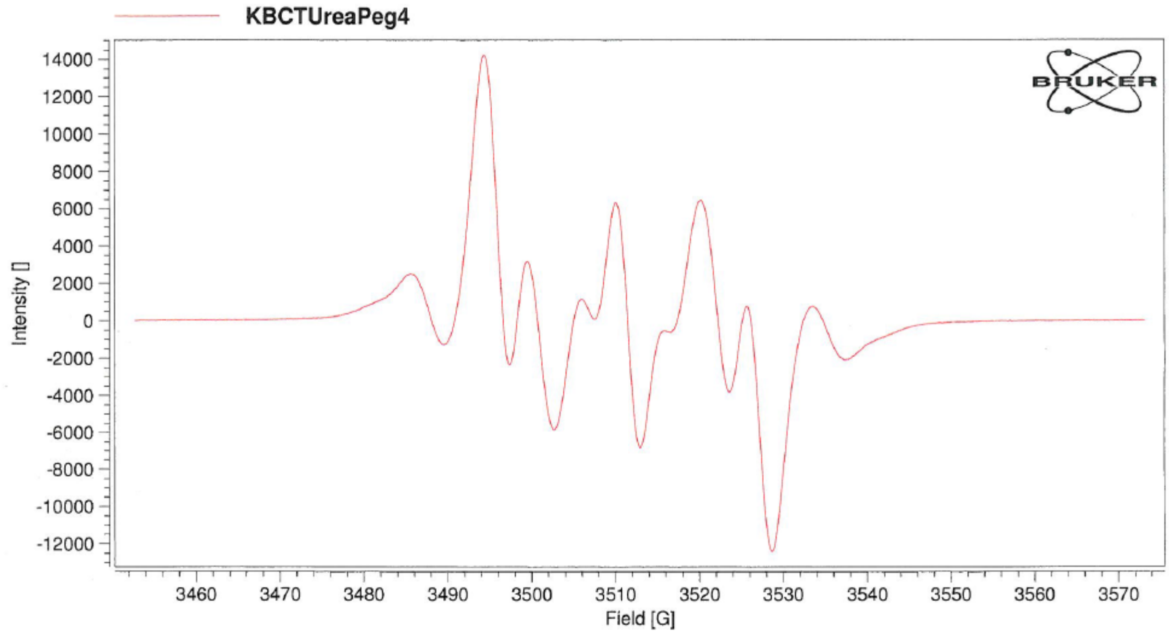
KBCTUreaPeg4
BRUKER
Intensity []
Field [G]

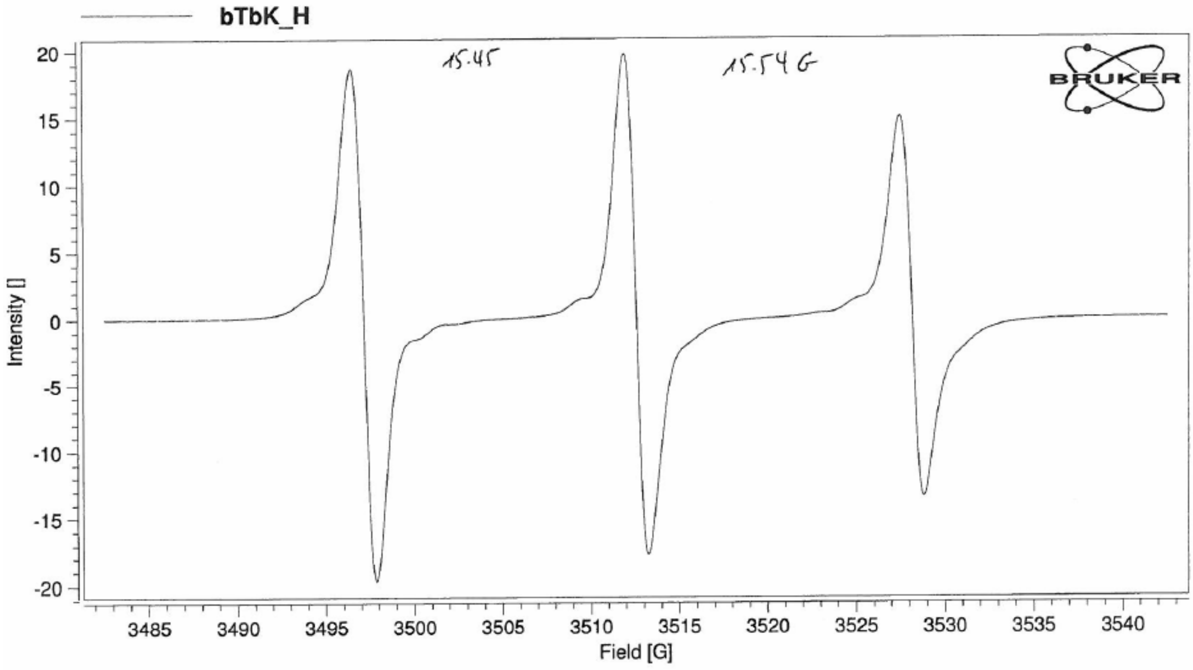
bTbK_H
15.45
15.54 G
BRUKER
Intensity []
Field [G]

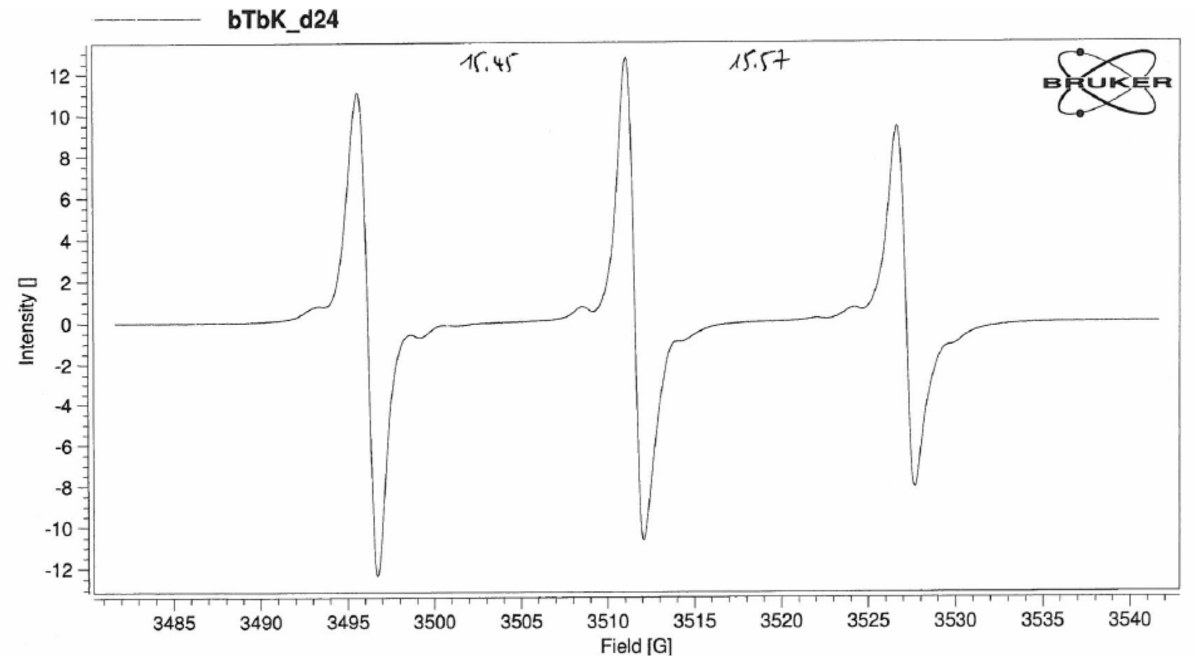
bTbK_d24
15.45
15.57
BRUKER
Intensity []
12
10
8
6
4
2
0
-2
-4
-6
-8
-10
-12
3485
3490
3495
3500
3505
3510
3515
3520
3525
3530
3535
3540
Field [G]

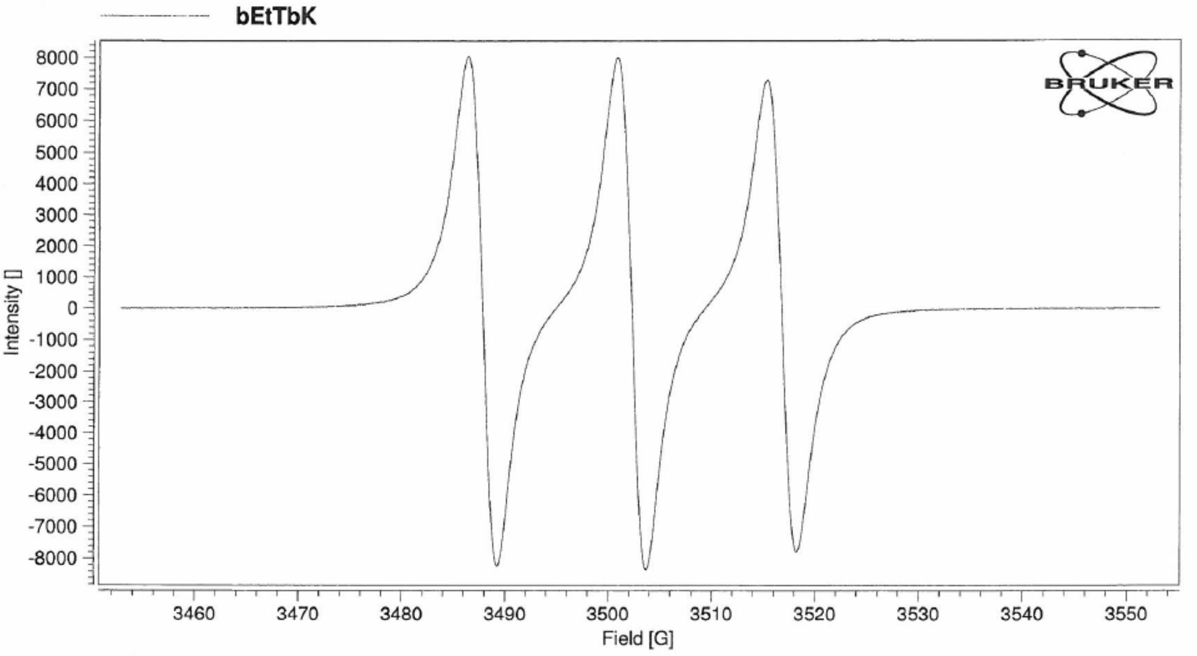
bEtTbK
BRUKER
Intensity []
8000
7000
6000
5000
4000
3000
2000
1000
0
-1000
-2000
-3000
-4000
-5000
-6000
-7000
-8000
3460
3470
3480
3490
3500
3510
3520
3530
3540
3550
Field [G]

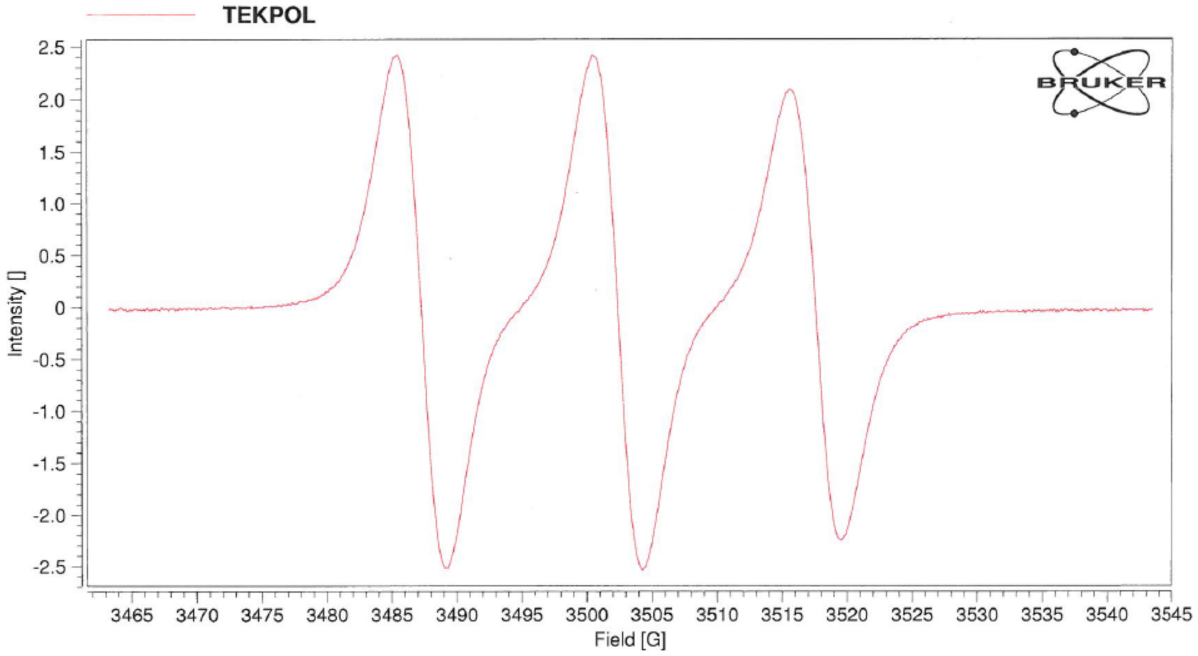
TEKPOL
BRUKER
Intensity []
Field [G]
2.5
2.0
1.5
1.0
0.5
0
-0.5
-1.0
-1.5
-2.0
-2.5
3465 3470 3475 3480 3485 3490 3495 3500 3505 3510 3515 3520 3525 3530 3535 3540 3545

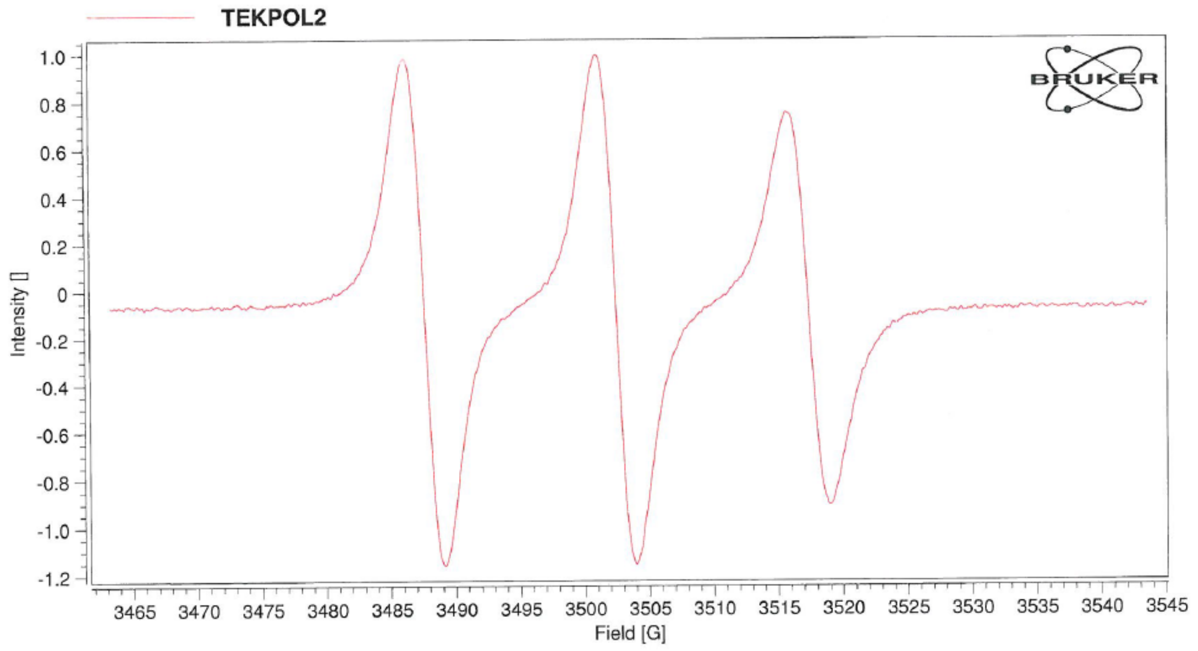
TEKPOL2
BRUKER
Intensity []
Field [G]
1.0
0.8
0.6
0.4
0.2
0
-0.2
-0.4
-0.6
-0.8
-1.0
-1.2
3465 3470 3475 3480 3485 3490 3495 3500 3505 3510 3515 3520 3525 3530 3535 3540 3545

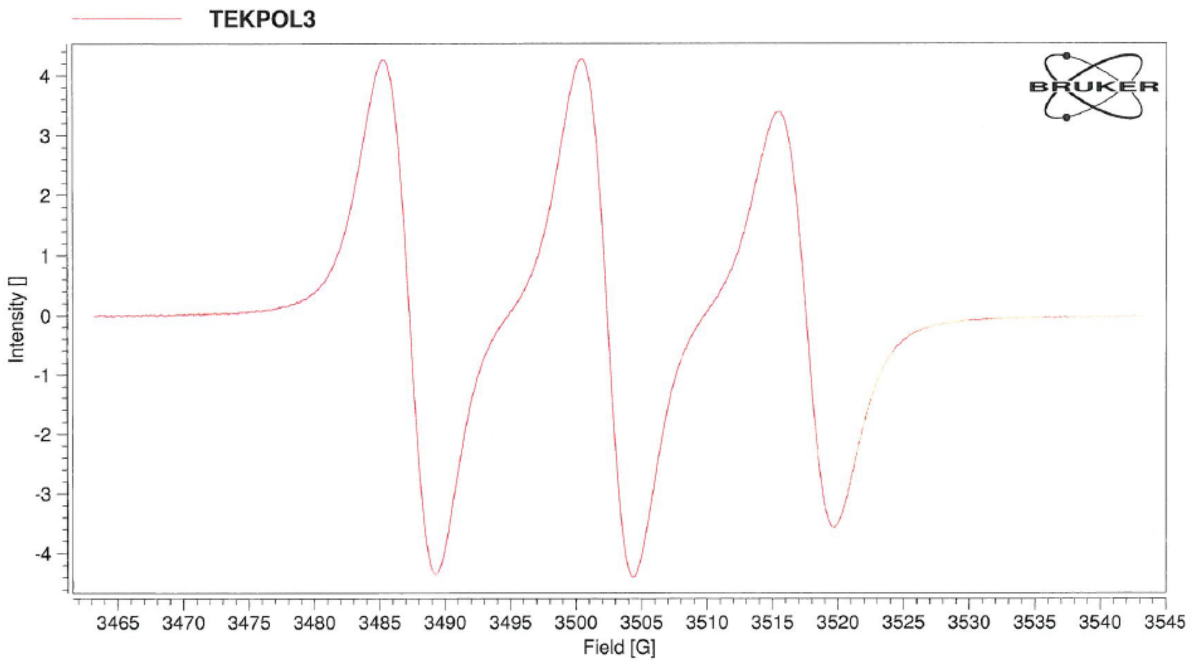

TEKPOL3
BRUKER
Intensity []
Field [G]

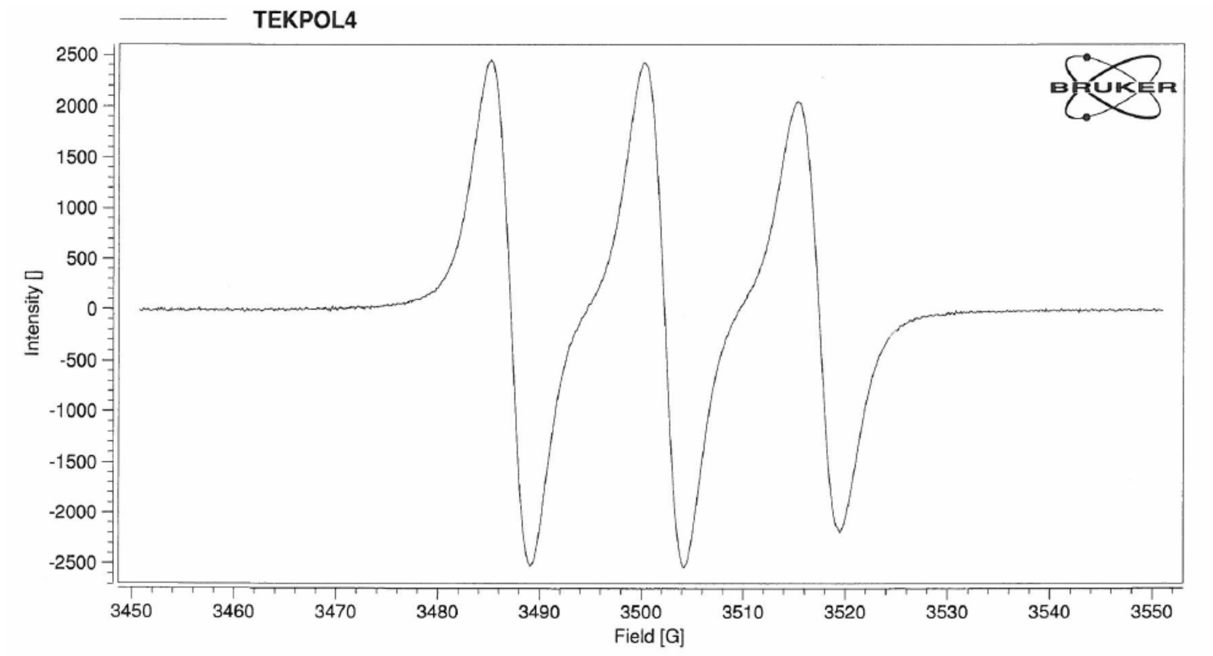

TEKPOL4
BRUKER
Intensity []
Field [G]

References

(1) Sakai, K. ; Yamada, K. ; Yamasaki, T. ; Kinoshit, Y. ; Mito, F. ; Utsumi, H. *Tetrahedron* **2010**, 66, 2311-2315.

(2) Suwal, S.; Senevirathne, C.; Garre, S.; Pflum, M.K.H. *Bioconjugate Chem*. **2012**, 23, 2386
(3) Lin, Y. J.; Teicher, B. A.; Halpern, H. J. *J. Labelled Comp. Rad.* **1990**, *28*, 621.

(4) Carrozza, P.; Ferri, G. *Eur. Pat. Appl*., **1996**, EP 729947 A1 19960904.

(5) Matsuki, Y.; Maly, T.; Ouari, O.; Karoui, H.; Le Moigne, F.; Rizzato, E.; Lyubenova, S.; Herzfeld, J.; Prisner, T.; Tordo, P.; Griffin, R. G. *Angew. Chem., Int. Ed.* **2009**, *48*, 4996.

(6) Zagdoun, A.; Casano, G.; Ouari, O.; Schwarzwalder, M.; Rossini,̈ A. J.; Aussenac, F.; M, Y.; G, J.; Coperet, C.; Lesage, A.; Tordo, P.;́ Emsley, L. *J. Am. Chem. Soc.* **2013**, *135*, 12790.

(7) Zagdoun, A.; Casano, G.; Ouari, O.; Lapadula, G.; Rossini, A. J.; Lelli, M.; Baffert, M.; Gajan, D.; Veyre, L.; Maas, W. E.; Rosay, M.; Weber, R. T.; Thieuleux, C.; Coperet, C.; Lesage, A.; Tordo, P.;́ Emsley, L. *J. Am. Chem. Soc.* **2012**, *134*, 2284.

(8) Sauvée, C.; Rosay, M.; Casano, G.; Aussenac, F.; Weber, R. T.;́ Ouari, O.; Tordo, P. *Angew. Chem., Int. Ed.* **2013**, *52*, 10858.

(9) Song, C.; Hu, K.-N.; Joo, C.-G.; Swager, T. M.; Griffin, R. G. *J. Am. Chem. Soc.* **2006**, *128*, 11385.

(10) Sauvée, C.; Casano, G. ; Ouari, O. ; Tordo, P. ; Aussenac, F. ; Rosay, M. *PCT Int. Appl.* **2014**, WO 2014167121 A1 20141016.

(11) Shiono, K.; Nagayama, H.; Kumai, H., *JP2010280629* (A).

(12) D. A. Neumayer, J. A. Belot, R. L. Feezel, C. Reedy, C. L. Stern, T. J. Marks, L. M. Liable-Sands, A. L. Rheingold, *Inorg. Chem***.** **1998**, *37*, 5625.

(13) Akai, S.; Ishida, S.; Hatanaka, K.; Ishii, T.; Harada, N.; Tsukada, H.; Oku, N. *Mol. Pharmaceutics* **2011**, *8*, 302.

(14) Williams, P.D.; McCauley, J.A.; Bennett, D. J.; Bungard, C.J.; Chang, L.; Chu, Xin-Jie; Dwyer, M. P.; Holloway, M.K.; Keertikar, K. M.; Loughran, H.M.; Manikowski, J.J.; Morriello, G. J.; Shen, Dong-M.; Sherer, E.C.; Schulz, J.; Waddell, S.T.; Wiscount, C.M.; Zorn, N.; Tummanapalli, S.; Sivalenka, V.; Hu, B.; Ji, T.; Zhong, B. *PCT Int. Appl.* **2015**, WO 2015013835 A1 20150205

(15) Balasubramanian, M.; D'Souza, A. *Tetrahedron*, **1968**, *24*, 5399.

(16) Davioud-Charvet, E.; Wenzel, I.N.; Müller, T.J.J.; Hanquet, G.; Lanfranchi, D.A.; Leroux, F.; Gendron, T. *PCT Int. Appl.* **2011**,
(17) Zhou, L.; Liu, X.; Ji, J.; Zhang, Y.; Hu, X.; Lin, L.; Feng, X. *J. Am. Chem. Soc.* **2012**, *134*, 17023.

(18) Johansson, M.; Thornqvist-Oltner, V.; Toftered , J.; Wensbo, D.; Dalence M. *WO 2010097410A1*
